# Genomic consequences of domestication of the Siamese fighting fish

**DOI:** 10.1126/sciadv.abm4950

**Published:** 2022-03-09

**Authors:** Young Mi Kwon, Nathan Vranken, Carla Hoge, Madison R. Lichak, Amy L. Norovich, Kerel X. Francis, Julia Camacho-Garcia, Iliana Bista, Jonathan Wood, Shane McCarthy, William Chow, Heok Hui Tan, Kerstin Howe, Sepalika Bandara, Johannes von Lintig, Lukas Rüber, Richard Durbin, Hannes Svardal, Andres Bendesky

**Affiliations:** 1 Zuckerman Mind Brain Behavior Institute, Columbia University, New York, NY, USA.; 2 Department of Ecology, Evolution and Environmental Biology, Columbia University, New York, NY, USA.; 3 Department of Biological Sciences, Columbia University, New York, NY, USA.; 4 Department of Biology, University of Antwerp, 2020 Antwerp, Belgium.; 5 Department of Biology, KU Leuven, 3000 Leuven, Belgium.; 6 Wellcome Sanger Institute, Cambridge, UK.; 7 Department of Genetics, University of Cambridge, Cambridge, UK.; 8 Lee Kong Chian Natural History Museum, National University of Singapore, Singapore, Singapore.; 9 Department of Pharmacology, Case Western Reserve University, Cleveland, OH, USA.; 10 Aquatic Ecology and Evolution, Institute of Ecology and Evolution, University of Bern, Bern 3012, Switzerland.; 11 Naturhistorisches Museum Bern, Bern 3005, Switzerland.; 12 Naturalis Biodiversity Center, 2333 Leiden, Netherlands.

## Abstract

Siamese fighting (betta) fish are among the most popular and morphologically diverse pet fish, but the genetic bases of their domestication and phenotypic diversification are largely unknown. We assembled de novo the genome of a wild *Betta splendens* and whole-genome sequenced 98 individuals across five closely related species. We find evidence of bidirectional hybridization between domesticated ornamental betta and other wild *Betta* species. We discover *dmrt1* as the main sex determination gene in ornamental betta and that it has lower penetrance in wild *B. splendens*. Furthermore, we find genes with signatures of recent, strong selection that have large effects on color in specific parts of the body or on the shape of individual fins and that most are unlinked. Our results demonstrate how simple genetic architectures paired with anatomical modularity can lead to vast phenotypic diversity generated during animal domestication and launch betta as a powerful new system for evolutionary genetics.

## Teaser

A Lego-like genetic logic for ornamental traits underlies the diversification of betta fish during domestication.

## INTRODUCTION

Domesticated animals have provided important insights into the genetic bases of a wide range of morphological, physiological, and behavioral traits. Because of their intimate relationship with people, domesticates have also furthered our understanding of human history and culture and of our interactions with other species (*1*). Genetic studies of animal domestication, however, have largely focused on mammals and birds (*1*, *2*), and only few genome-wide analyses of fish domestication have been performed (*3*–*5*).

Siamese fighting fish have been selectively bred for fighting in Southeast Asia for centuries, with reports dating back to as early as the 14th century A.D. in Thailand, making them one of the oldest fish domestications (*6*). Starting in the early 20th century, Siamese fighting fish also began to be bred for ornamental purposes, becoming one of the world’s most popular pet fish, commonly known as betta (*7*). Although it is generally presumed, on the basis of morphology and few genetic markers (*8*, *9*), that domesticated fighting fish derive mainly from *Betta splendens*, it has been suggested that other closely related species (collectively called the *B. splendens* species complex) may have contributed to modern varieties (*10*). Ornamental betta have been diversified from their short-finned ancestors into an astonishing array of fin morphologies, colors, and pigmentation patterns, providing a rich phenotypic repertoire for genetic analysis. This remarkable and long history of domestication for fighting, followed by breeding for ornamental purposes, combined with one of the smallest vertebrate genomes at only ~450 megabase pairs (Mb) (*11*–*13*), makes betta an appealing subject for evolutionary genetic studies of domestication. Here, we use a synergistic combination of population and quantitative genetic approaches to investigate the historical processes and molecular changes that lead to the domestication and phenotypic diversification of betta fish.

## RESULTS

### A wild *B. splendens* reference genome

We generated a high-quality reference genome assembly of wild *B. splendens* using long-read PacBio technology, optical mapping with BioNano, further scaffolding with 10X Genomics linked reads, polishing with Illumina short reads, and finishing with manual curation (*14*). We obtained a reference genome composed of 441 Mb, of which 98.6% is assigned to the 21 chromosomes expected from its karyotype (*15*). The contig N50 reached 2.50 Mb and the scaffold N50 20.13 Mb, meeting the standards set forth by the Vertebrate Genomes Project (*16*). To annotate the genome, we performed RNA sequencing from male and female brain, fin, liver, spleen, and gonad. This annotated reference genome is now the representative *B. splendens* reference in the National Center for Biotechnology Information (NCBI) (fBetSpl5.3, GCA_900634795.3).

To discover structural chromosomal rearrangements that may have arisen during domestication, we performed whole-genome alignments of our wild *B. splendens* reference to three ornamental betta references (*11*–*13*) and to *Anabas testudineus* (climbing perch) as an outgroup (*8*, *16*). Except for a possible large intrachromosomal rearrangement of chromosome 16 in ornamental betta, the genome was largely syntenic between wild *B. splendens* and ornamental betta (fig. S1 and note S1).

### Complex evolutionary relationships between *Betta* species

**Fig. 1.**
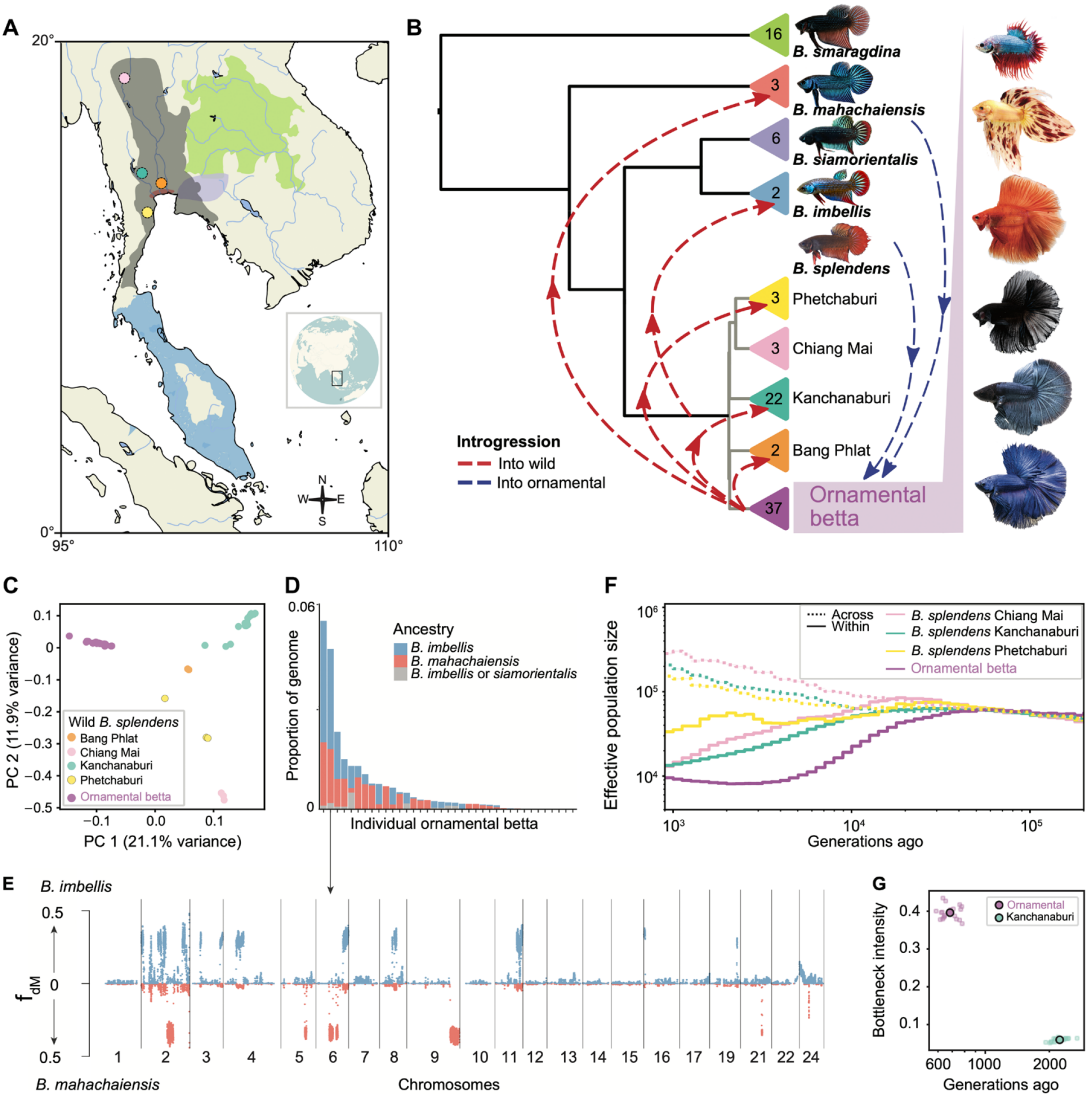
*Betta* phylogeny, gene flow, and demographic history. (**A**) Distribution ranges of *B. splendens* species and sampling locations. Dots denote sampling locations of *B. splendens* populations. Colors according to (B) and *B. splendens* in gray. (**B**) Species and population relatedness based on NJ of group pairwise genetic differences. Arrows denote introgression events involving specific samples. Triangles contain sample numbers. Photos show representative males of ornamental varieties; from top to bottom: crowntail plakat, dalmatian veiltail, orange doubletail, superblack halfmoon, steel halfmoon, and royal blue halfmoon. Image credits: Kasey Clark (*B. smaragdina*, *B. mahachaiensis*, *B. imbellis*, and ornamental betta), Frank Sriborirum (*B. siamorientalis*), and Madison Lichak, Columbia University (*B. splendens*). (**C**) Principal components analysis (PCA) of genetic variation among *B. splendens* samples. PCA including other species is shown in fig. S8. (**D**) Proportion of genome introgressed from non-*splendens* species in each ornamental individual based on f_dM_ and regional trees. (**E**) Genome-wide f_dM_ plot of ornamental betta Orn45 (p1, other ornamental bettas; p2, Orn45; p3, *B. mahachaiensis* or *imbellis*; outgroup, *B. compuncta*). (**F**) Effective population sizes as estimated by Relate within populations (solid lines) and between wild *B. splendens* populations and ornamental betta (dashed lines). (**G**) Inference of bottleneck timing and intensity of ornamental and wild *B. splendens* from Kanchanaburi using fastsimcoal2. Dots denote independent runs, and outlined dot denotes the average. Bottleneck intensity = (bottleneck duration)/(2*Ne bottleneck).

To determine the genetic origin of ornamental betta and understand their relationships with species of the *B. splendens* complex, we sequenced to ~15× coverage the whole genomes of (i) 37 ornamental betta from different sources, representing a diversity of ornamental traits (Fig. 1, A and B, and table S1); (ii) 58 wild-caught individuals, including representatives of all species of the *B. splendens* complex (except for *Betta stiktos*), and four populations of *B. splendens* from different parts of its natural range (Fig. 1A); and (iii) an outgroup (*Betta compuncta*). We aligned the sequencing reads to our *B. splendens* reference genome then called and filtered variants to generate a final set of 27.8 million phased biallelic single-nucleotide polymorphisms (SNPs).

We first assessed relationships across the wild species of the *B. splendens* complex by constructing neighbor-joining (NJ) and maximum likelihood–based phylogenies (Fig. 1B and fig. S3, A and B). We observed strong bootstrap support for *Betta smaragdina* as the outgroup to the other *B. splendens* complex species, with *Betta mahachaiensis* as the outgroup to the remaining species. *Betta imbellis* and *Betta siamorientalis* together form a sister clade to all wild *B. splendens* populations.

We then tested for evidence of evolutionary processes that violate tree-like species relationships, such as hybridization, by computing ABBA-BABA statistics [Patterson’s *D* and f4 admixture ratio (*17*)] for all triplets of individuals organized according to the phylogeny. This analysis revealed widespread patterns of excess allele sharing between nonsister species, suggesting that the speciation history of these groups was complex, involving either structured ancestral populations, cross-species gene flow, or both (Fig. 1B, fig. S4A, and note S2). Notably, two of three *B. mahachaiensis* samples and one of the two *B. imbellis* samples showed highly significant excess allele sharing with *B. splendens* populations compared to their conspecifics sampled from different locations, consistent with gene flow from *B. splendens* into particular populations of these species (fig. S4, A and B, and notes S2 and S3).

### Ornamental betta derive from *B. splendens* but have variable contributions from other species

Adding the ornamental betta samples to the phylogeny, we found that they cluster with *B. splendens* (Fig. 1B and fig. S3C). This result was also observed through principal components analysis (PCA), where ornamental betta showed no apparent loading on axes representing non-*splendens* species (fig. S8A). In both phylogenies and PCA, ornamentals form a clearly defined group distinct from all wild *B. splendens* populations (Fig. 1, B and C, and fig. S8B). These results indicate that ornamental betta are genetically most similar to *B. splendens*.

To test whether ornamental betta carry non-*splendens* ancestry, we computed all ABBA-BABA tests of the form *D* (ornamental except focal, focal ornamental; non-*splendens* species, outgroup) (fig. S5, G to J, and note S4). These tests revealed that 76% (28 of 37) of ornamental betta carry significant ancestry from non-*splendens* species, a pattern that is generally not seen for wild *B. splendens* individuals (fig. S5K). To examine the chromosomal distribution of non-*splendens* ancestry in these individuals, we computed regional ABBA-BABA statistics (f_dM_) (*18*–*20*) along their genomes and confirmed non-*splendens* ancestry in high-f_dM_ regions by constructing local gene trees (Fig. 1, D and E, and fig. S9). The analyses revealed that signals of excess allele sharing are driven by genomic tracts where one or, more rarely, both haplotypes of the focal sample clustered with *B. imbellis* or *B. mahachaiensis* (fig. S9). Cumulatively, these tracts encompass between 0 and 6% of the genomes of ornamental betta (Fig. 1D). The tracts are generally different among individuals, and none are shared by all individuals of particular ornamental phenotypes (red, blue, veiltail, and crowntail), suggesting that these ornamental phenotypes are not caused by alleles from non-*splendens* species (fig. S12). The ornamental sample with the second highest levels of introgression from other species is particularly interesting, since some of its chromosomes are a mosaic of alternating regions of *B. imbellis* and *B. mahachaiensis* ancestry, consistent with a natural or man-made hybrid of those species having been backcrossed into ornamental betta (Fig. 1E). Together, our analyses indicate that ornamental betta are clearly derived from *B. splendens*, yet most individuals have relatively recent contributions from *B. mahachaiensis* and *B. imbellis*.

### Ornamental betta introgression is widespread among wild *Betta*

The topology of relationships between wild *B. splendens* populations in the phylogenies changed after including ornamental bettas (fig. S3, A and C, and notes S4 and S5). To further investigate this, we computed ABBA-BABA statistics within the framework of the phylogeny including ornamentals and assessed each individual’s relationship with respect to the other species of the *B. splendens* species complex, as well as ornamentals (fig. S6A). Together, these analyses revealed strong evidence for ornamental betta ancestry in two of three wild *B. mahachaiensis* samples and in individuals from three of four populations of wild *B. splendens* (note S4). Investigating the signals along the genome, we found that for the two *B. mahachaiensis* samples, *mahachaiensis*-like and ornamental-like haplotypes alternate at near-chromosome scale, suggesting an ornamental ancestor only a few generations back (fig. S7B). Gene flow from ornamental betta into *B. mahachaiensis* is possibly facilitated by the close proximity of the Mahachai region to the Bangkok metropolis. Conversely, for wild *B. splendens* individuals with ornamental betta ancestry, the genome-wide signals of excess allele sharing with ornamentals were diffusely distributed along the chromosomes with only a few relatively short, clearly distinguishable ornamental haplotypes (fig. S7A), suggesting that there was enough time for introgressed haplotypes to be broken down by recombination. In summary, ornamental introgression into wild *Betta* seems to be geographically widespread and to have happened both long ago and very recently. This finding is perhaps related to the practice by breeders of releasing excess domesticated betta into the wild and may constitute a conservation threat to wild *Betta* populations.

### Demographic history and domestication of *B. splendens*

To infer the demographic history of wild and domesticated *B. splendens*, we performed coalescence-based demographic analysis. To date events, we first needed to know the germline mutation rate. To determine this rate, we sequenced an ornamental trio and a quartet to >30× coverage and found the mutation rate to be 3.75 × 10^−9^/base pair (bp) per generation [95% confidence interval (CI): 9.05 × 10^−10^ to 9.39 × 10^−9^]. This rate is similar to the rate previously inferred for cichlids (*21*) and approximately threefold lower than that of humans (*22*). Although the generation time of *B. splendens* in the wild is not known, betta in captivity are bred at around 7 months of age (*7*). Our demographic analyses using Relate (*23*), excluding genomic regions with introgression to avoid biasing coalescence time estimates, suggest that the lineages leading to present-day ornamental and wild populations began to split around 40,000 generations (~23,000 years) ago (Fig. 1F and fig. S10, A, B, and D).

Next, we queried our genomic data for signatures of a recent, strong population bottleneck, as would be expected if a small number of animals are taken from the wild and propagated in captivity in the process of domestication. Consistent with such a bottleneck, we observe elevated linkage disequilibrium (LD) in ornamental betta relative to wild *B. splendens* (fig. S11B). Since, in the absence of very large sample sizes, Markovian coalescence–based approaches such as Relate do not accurately estimate recent demographic changes, we used fastsimcoal2 (*24*), which uses the information in the site frequency spectrum. As we show by extensive simulations, fastsimcoal2 can accurately time bottlenecks of varying intensities as recent as 100 generations ago and is robust to the overall demographic history (fig. S10E and note S6). Using this method, we found evidence for a bottleneck in ornamental betta ~680 generations ago (~400 years; ~160 to 1670 years based on mutation rate 95% CI; Fig. 1G) with an average of 79% of the present-day sampled lineages finding a common ancestor during the bottleneck (note S6). Bottleneck duration and strength are generally difficult to disentangle (*25*), but, as an illustration, for a bottleneck duration of 20 generations, the estimated intensity would correspond to a reduction to only 25 breeding individuals. When performing the same analysis on wild *B. splendens*, we only detected a much older and weaker bottleneck ~2240 generations ago of only ~0.14× the intensity of the ornamental bottleneck. Thus, our results are consistent with a strong bottleneck in the ornamental lineage.

### Genetic signals of selection in ornamental betta

**Fig. 2.**
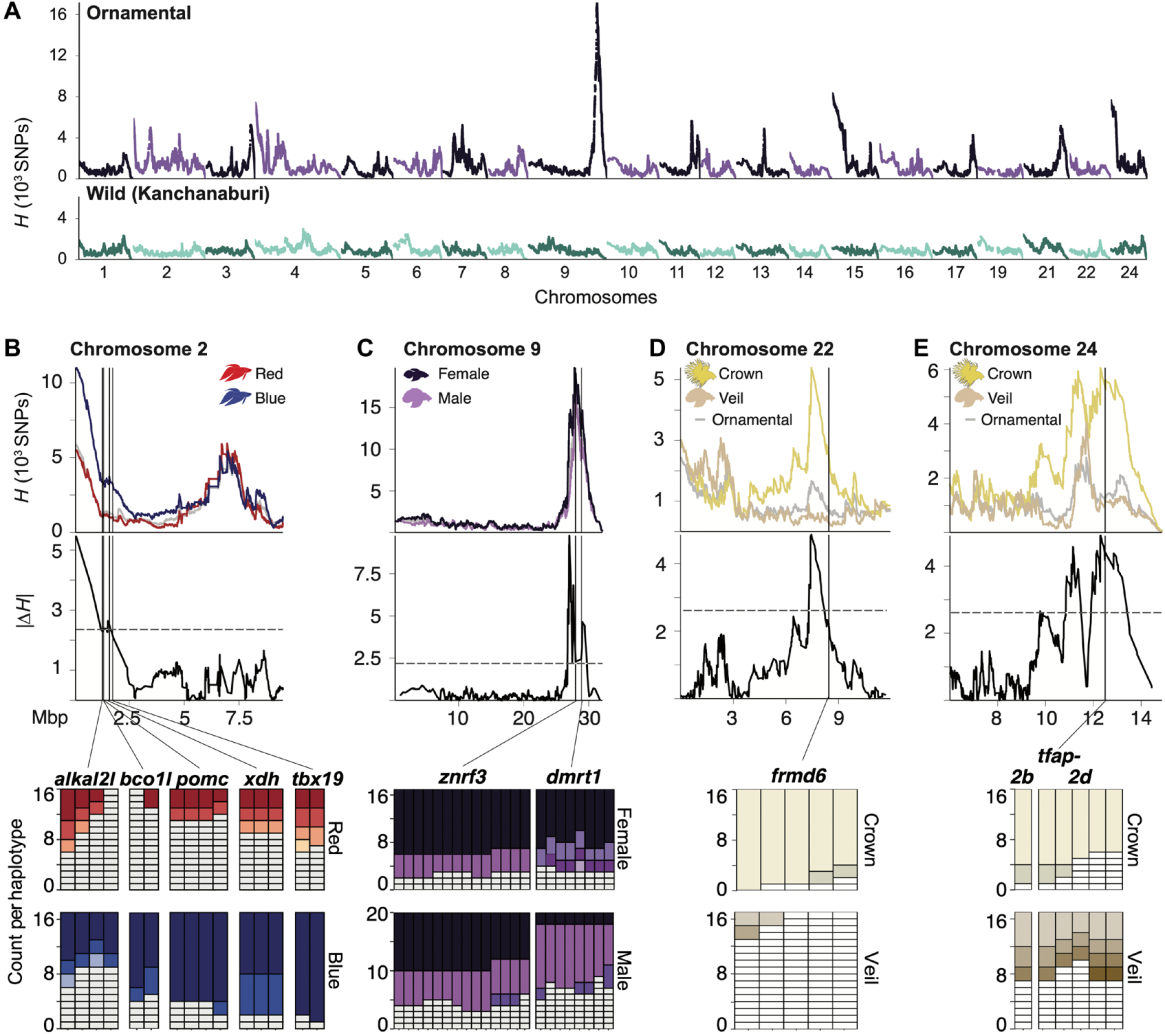
Genomic signals of selection in ornamental betta. (**A**) Genome-wide *H*-scan within ornamental betta (*n* = 37) and within wild *B. splendens* from Kanchanaburi (*n* = 24). (**B** to **E**) Top: *H*-scan close-up of ornamentals (gray line). Middle: Ornamentals separated by (B) color (red, 17; blue, 17), (C) sex (female, 17; male, 20), and (D and E) fin morphology (crown, 16; veil, 18). Gray dashed lines denote genome-wide threshold of significance (α = 0.05) for the absolute difference in *H* (|Δ*H*|) between ornamentals separated by color, sex, and fin type. (B to E) Bottom: Distribution of haplotypes in genes identified as outliers in both *H*-scan and G12 and statistically different between ornamentals separated by color, sex, and fin morphology; see Figs. 3 to 5. White denotes haplotypes observed in single fish.

Genetic variants that increase fitness in captivity or that are associated with phenotypic traits actively selected by breeders are expected to increase in frequency during domestication. To discover these loci with signatures of selective sweeps in ornamental betta, we searched for extended homozygosity tracts across 37 ornamental betta using *H*-scan, which can detect soft and hard selective sweeps and performs well in dogs, another domesticated species (*26*). We used two other tests to detect selected loci: G12, which looks for high-frequency haplotypes (*27*) and Tajima’s *D*, a frequency-based test (*28*). *H*-scan identified prominent peaks in multiple chromosomes (Fig. 2A), and these peaks coincided with G12 peaks and Tajima’s *D* values below zero, all supporting evidence of selective sweeps in ornamental betta (fig. S12). Equivalent selection scans using whole-genome sequencing of 24 wild *B. splendens* did not reveal clear signals, which is unlikely due to the smaller sample size, as *H*-scan and G12 peaks remained in a downsampled set of 24 ornamentals (fig. S13, A and B). Notably, selection peaks were not located in genomic regions with low recombination rates, suggesting that selection signals are unlikely to be driven by long haplotypes resulting from reduced recombination (fig. S13C). These results are consistent with footprints of selection in ornamental betta being related to the domestication process.

The majority (34 of 37) of the ornamental betta that we sequenced represent four of the most popular varieties along two phenotypic dimensions: color and fin morphology. The fish were royal blue (*n* = 17), solid red (*n* = 17), veiltail (*n* = 18), and crowntail (*n* = 16), represented by males (*n* = 20) and females (*n* = 17). Veiltails are characterized by large, flowing caudal fins, and crowntails have fins that are webbed between the rays (Fig. 1B). To determine whether the footprints of selection that we detected were driven by fish of a particular variety, we compared *H*-scan and haplotype frequencies across subsets of fish representing these traits (Fig. 2, B to E).

The most prominent selection peak shared across ornamentals but absent in wild *B. splendens* falls on chromosome 9 and is centered on *zinc and ring finger 3* (*znrf3*), which is required for the formation of fin rays in zebrafish (*29*). A peak close to *znrf3*, centered on *double-sex and mab-3–related transcription factor 1* (*dmrt1*), became apparent when comparing males to females (Fig. 2C). *dmrt1* is critical for gonad development in vertebrates and functions as the sex determination gene in several fish species (*30*–*32*), in *Xenopus laevis* frogs (*33*), and in birds (*34*), suggesting that *dmrt1* has a role in sex determination in betta.

A strong sweep in blue fish on chromosome 2 harbors multiple genes involved in pigmentation (Fig. 2B): *proopiomelanocortin* (*pomc*), which encodes alpha- and beta-melanocyte–stimulating hormones; *T-box transcription factor 19* (*tbx19*), which encodes a transcription factor expressed specifically in pituitary cells that will express *pomc* (*35*); *xanthine dehydrogenase* (*xdh*), which encodes an enzyme whose homologs synthesize yellow-red pteridine pigments (*36*); *ALK and LTK-ligand 2–like* (*alkal2l*), which encodes a cell signaling molecule important for the development of iridophores (*37*, *38*); and *beta-carotene oxygenase like-1* (*bco1l*), which encodes an enzyme whose homologs metabolize orange-red carotenoid pigments (*39*). These results suggest that one or more of these pigmentation genes were a target of selection by betta breeders. Two selection peaks, one on chromosome 22 and another on chromosome 24, were not detected when all ornamental fish were combined or in an analysis including only veiltail fish but were significant in the subset of crowntail fish (Fig. 2, D and E), suggesting their importance to crowntail fin morphology.

### The evolution of sex determination

**Fig. 3.**
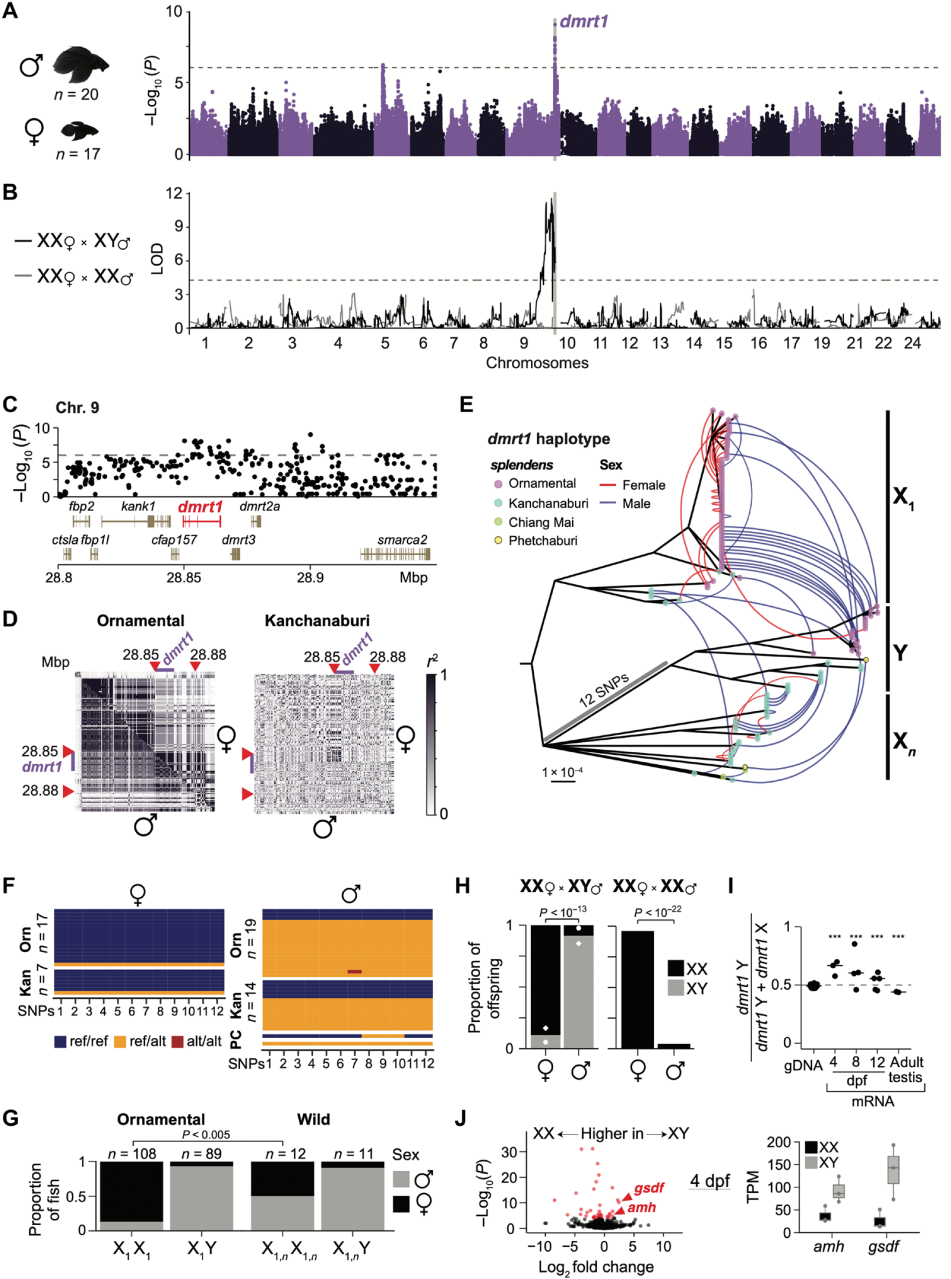
*dmrt1* is a sex determination gene in ornamental betta. (**A**) Manhattan plot for GWAS of gonadal sex in ornamental betta. Dashed line denotes the genome-wide significance threshold. (**B**) QTL mapping of two F_2_ intercrosses: *dmrt1*_X_1_X_1_ female × *dmrt1*_X_1_Y male (black line) and *dmrt1*_X_1_X_1_ female × *dmrt1*_X_1_X_1_ male (gray line). Dashed line denotes the genome-wide significance threshold (α = 0.05). (**C**) GWAS zoom-in of the *dmrt1* locus. (**D**) LD plot for chromosome 9 surrounding the *dmrt1* locus for ornamental betta and wild *B. splendens* from Kanchanaburi. Upper triangle, females; lower triangle, males. Red arrowheads denote the region of the *dmrt1* haplotype in (E). Purple bar denotes *dmrt1*. (**E**) Maximum likelihood phylogeny with ≥80% bootstrap support for the *dmrt1* haplotype across *B. splendens*, rooted by *B. siamorientalis*. Tips represent one of the two alleles of a sample, colored by population. Arches link the two alleles of each sample and are colored by sex. (**F**) Genotypes of males and females from ornamental betta and wild *B. splendens* across 12 SNPs present in all samples within the *dmrt1_*Y group. P, Phetchaburi; C, Chiang Mai. (**G**) Sex ratios for *dmrt1* haplotypes in ornamental betta and wild *B. splendens*. *P* value by Fisher’s exact test. (**H**) Average haplotype ratios across sex for offspring from two *dmrt1*_X_1_X_1_ female × dmrt1_X_1_Y male crosses (diamond, *n* = 101; circle, *n* = 112) and *dmrt1*_X_1_X_1_ male × *dmrt1*_X_1_X_1_ female cross (*n* = 100). *P* value by Fisher’s exact test. (**I**) Allele-specific expression of *dmrt1* across days post fertilization (dpf) for *dmrt1*_X_1_Y larvae from a *dmrt1*_X_1_X_1_ × *dmrt1*_X_1_Y cross. Dots denote individual offspring. ***P < 0.001 by binomial test. (**J**) Left: Differential mRNA expression between three *dmrt1*_X_1_Y and three *dmrt1*_X_1_X_1_ 4-dpf larvae. Red denotes expression differences where *P* < 10^−6^. Right: Transcripts per million (TPM) of two genes important for male sex development: antimullerian hormone (*amh*) and gonadal soma–derived factor (*gsdf*).

To test whether the locus containing *dmrt1*, which had evidence of a selective sweep in ornamental betta, is involved in sex determination, we performed a genome-wide association study (GWAS) using gonadal sex as the trait. We focused on ornamental betta since we had a large enough sample size (20 males and 17 females) to detect variants with large effect on sex. A ~30-kb region overlapping *dmrt1* was strongly associated with sex, with 16 of 17 females being homozygous at the most strongly associated SNPs, while 16 of 20 males were heterozygous (Fig. 3, A and C). We call “Y” the male-specific allele of *dmrt1* and “X” the allele present in both males and females. These results strongly implicate *dmrt1* as the sex determination gene in ornamental betta and indicate that males are the heterogametic sex.

The reference genome was generated from a wild male *B. splendens*, so genomic sequences present only in females or only in ornamental betta would not be represented in the SNPs that we used for GWAS. Only 0.5% of sequencing reads of individuals from both sexes could not be mapped to the reference genome (male versus female, *P* = 0.64), indicating that there are no major sex-specific regions that are absent from the reference (fig. S14A). To test whether smaller-scale sequence differences were associated with sex, we performed a GWAS independent of the reference genome using k-mers from the sequencing reads. We found k-mers significantly associated with sex, and when we assembled those k-mers into contigs, they corresponded to *dmrt1*, consistent with the results from SNP-based GWAS (fig. S14B). We also assessed copy number variations across the genome, and none were significantly associated with sex (fig. S14C). Although sex chromosomes often carry chromosomal rearrangements, we found no evidence of an inversion in X or Y (Fig. 3D and Materials and Methods). These results indicate that, at this level of detection, only a small genomic region <30 kb within otherwise nonsexually differentiated chromosomes (autosomes) distinguishes female and male ornamental betta.

Because *dmrt1* had a strong signal of a selective sweep in ornamental betta, we hypothesized that *dmrt1*’s role in sex determination evolved rapidly during domestication. To explore the relationship between *dmrt1* and sex in wild and ornamental betta, we first built a phylogenetic tree of the *dmrt1* locus defined as a ~30-kb LD block (Fig. 3E). Consistent with the selective sweep, all ornamental females, but only one wild female, had a particular haplotype we call X_1_. In line with selection at the *dmrt1* locus in ornamental betta occurring preferentially on the X, its nucleotide diversity (1.14 × 10^−4^/bp) was lower than on the Y (1.66 × 10^−4^/bp; Y:X ratio of 1.46). In contrast, in wild *B. splendens*, Y had slightly lower diversity (4.07 × 10^−4^/bp) than X (4.71 × 10^−4^/bp; Y:X ratio of 0.86), indicating that the reduced diversity on X in ornamental betta is unlikely caused by a higher mutation rate in males compared to females, which would contribute to excess diversity on Y. As they evolve, proto-sex chromosomes often stop recombining in the heterogametic sex, leading to reduced diversity in the chromosome specific to that sex (*40*, *41*). However, we found neither evidence of reduced recombination around *dmrt1* (fig. S13C) nor evidence of rearrangements (which can prevent recombination) involving this region (Fig. 3D, fig. S1, and Materials and Methods). This agrees with the lack of karyotypic differences between the sexes in ornamental betta (*15*). Furthermore, Y has higher diversity than X in ornamental betta, opposite from the expectation arising from suppression of recombination between these alleles, supporting a role for selection acting preferentially on X.

We further explored the association between *dmrt1* and sex in wild and ornamental betta. In wild *B. splendens*, 50% (6 of 12) of XX individuals were female, and 91% (10 of 11; binomial *P* = 0.00048) of XY individuals were male (Fig. 3, F and G). While this evidence suggests that *dmrt1*_Y promotes maleness in wild *B. splendens*, it is possible that multiple sex determination systems segregate in the wild, similar to what is seen in African cichlids (*42*). In contrast, in ornamental betta, 87% (94 of 108; the 17 fish in the GWAS plus 91 independent samples; binomial *P <* 10^−12^) of XX individuals were female, and 93% (83 of 89; binomial *P <* 10^−12^) of XY individuals were male (Fig. 3G). These results are consistent with a higher penetrance of XX in promoting female development in ornamental betta than in wild *B. splendens* (Fisher’s exact test, two-tailed *P* = 0.005) and suggest that this effect contributed to the selective sweep around *dmrt1*.

Since *dmrt1* XX-XY status was not perfectly related to gonadal sex, we searched for additional sex-linked loci that may have been missed by GWAS. To do so, we performed two quantitative trait locus (QTL) mapping experiments, one in a cross between an XX female and an XY male, and another between an XX female and an XX male. In the XX × XY cross, 52% of the offspring were female, and we detected a single sex-linked locus encompassing *dmrt1* (Fig. 3B). In the XX × XX cross, 90% of the offspring were female, and no locus was linked to sex (Fig. 3, B and H). In the XX × XY cross, 85% of the XX offspring were female, and 90% of the XY offspring were male (Fig. 3H). However, in the XX × XX cross, all offspring were XX, yet 10% of these fish developed as males (Fig. 3H), confirming the incomplete penetrance of the XX-XY locus in sex determination, as has been observed in *Oryzias latipes* (medaka fish) that also bear a *dmrt1* XX-XY sex determination system (*43*). In summary, these results confirm that the *dmrt1* locus is strongly linked to sex in ornamental betta but that XX and XY are neither necessary nor sufficient to determine a particular sex.

To determine whether the X and Y transcripts of *dmrt1* are differentially expressed during sex determination, we performed allele-specific expression analyses in XY ornamental larvae at several time points after fertilization. The results indicated that the *dmrt1* Y allele constitutes 65% of the *dmrt1* mRNA molecules at 4 days post fertilization (dpf) and that this allelic bias progressively decreases at 8 and 12 dpf, until it reverses in adult testis, where only 45% of the *dmrt1* transcripts originate from the Y allele (Fig. 3I). This timing of *dmrt1* XY allele–specific expression is consistent with that of sex determination, since we found that by 4 dpf, XX and XY larvae have started the process of sex differentiation: XY larvae express higher levels of *gonadal soma–derived factor* (*gsdf*), a teleost-specific gene essential for testis development (*44*), and higher levels of *antimullerian hormone* (*amh*), a gene that promotes vertebrate male development (Fig. 3J). Each of these genes is the sex determination locus in other fish species (*44*, *45*) and is in separate chromosomes from *dmrt1* in betta, indicating that their sex-specific expression is a response in *trans* to *dmrt1*. Thus, the variants that distinguish *dmrt1* X from Y are associated with higher expression of the *dmrt1* Y allele in a manner that is temporally linked to sex differentiation, further implicating *dmrt1* as the major sex determination gene in ornamental betta.

### Genetic bases of coloration in ornamental betta

**Fig. 4.**
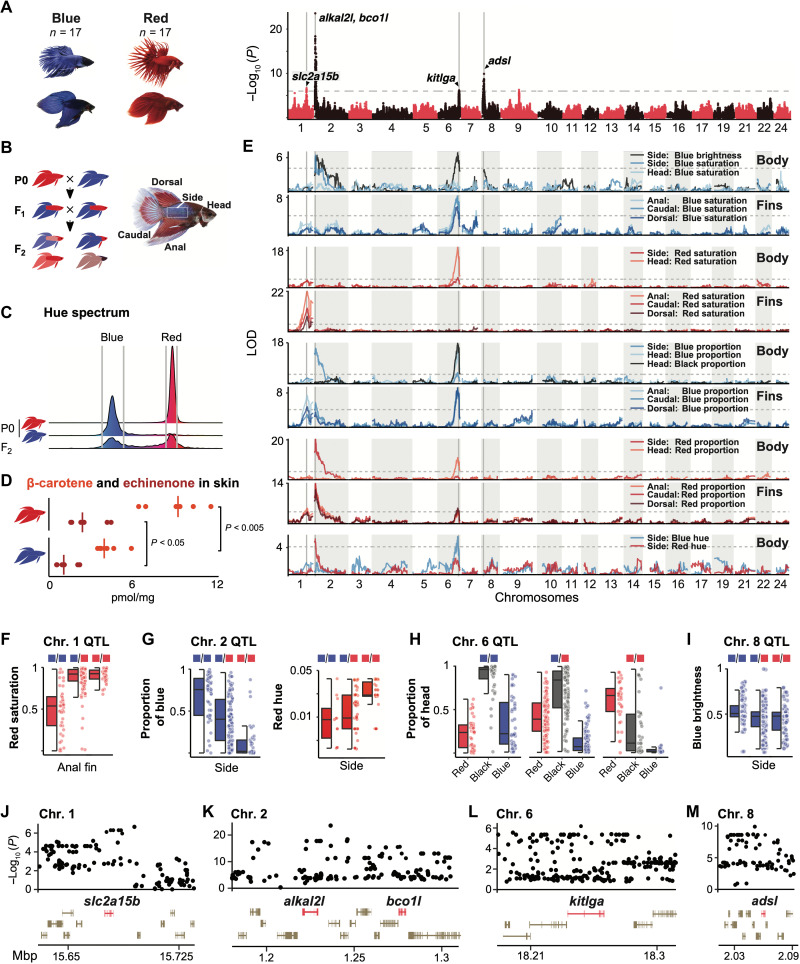
Genomic loci affecting coloration in ornamental betta. (**A**) Manhattan plot for GWAS of color between 17 blue and 17 red ornamental betta. Dashed line denotes the genome-wide significance threshold. (**B**) Schematic of the red × blue F_2_ intercross and photo of a hybrid annotated with the body parts analyzed. (**C**) Distribution of hues in the red and blue founder (P0) populations (red, *n* = 13; blue, *n* = 11) and F_2_ hybrids (*n* = 202). Gray vertical lines depict the hue intervals for red and blue assessed in the F_2_ population. (**D**) Concentration of β-carotene and echinenone in the skin of red and blue ornamental betta. *P* value by Mann-Whitney *U* test. (**E**) QTL mapping of color features across different body parts. Dashed lines denote the genome-wide significance threshold (α = 0.05). (**F** to **I**) Phenotypic distribution for QTL genotypes across the F_2_s (red and blue squares denote alleles inherited from red and blue P0s, respectively). (**J** to **M**) GWAS zoom-in across significant loci.

Ornamental betta breeders have generated a vast array of fish varieties (e.g., “royal blue”) that differ along multiple axes of coloration: hue, brightness, saturation, and the anatomical distribution of these features. To determine whether any of the genes we found to be under strong selection, as well as any others, contribute to coloration in ornamental betta, we performed a GWAS of the red (*n* = 17) and blue (*n* = 17) fish that were used for the selection scans (Fig. 4A and fig. S16A). Red and blue fish not only lie at opposite ends of the betta hue spectrum but also differ in their brightness and saturation (Fig. 4, A and C, and fig. S17). However, association mapping alone between pure red and pure blue fish, which are largely fixed for all these color features, cannot establish which of these features are affected by significant loci. Therefore, we also performed a QTL mapping experiment by generating a second-generation (F_2_) hybrid population of red-blue fish in which individual coloration components could segregate (Fig. 4B). In these 211 F_2_ hybrids, we measured the proportion of the anal, caudal, and dorsal fins; of the side of the body; and of the head that was red, blue, or very dark (which we refer to as black). We also measured the hue, brightness, and saturation of the red and blue areas on each body part and used these phenotypes for QTL mapping.

The strongest GWAS signal occurred between *augmentator-*α*2* (*alkal2l*) and *bco1l* on chromosome 2 (Fig. 4, A and K), a region with a large difference in selection sweep signal between blue and red fish (Fig. 2B). This GWAS peak was aligned with a QTL at which the allele swept in blue fish increased the proportion of blue and decreased the proportion of red on the fins and body of the hybrids (Fig. 4, E and G). This locus modulates blue saturation only on the body and not on the fins or the head (Fig. 4E). Consistent with genetic variation at the *alkal2l* locus mediating differences in blue saturation in the bodies between blue and red fish, we find that *alkal2l* mRNA is present at 210× higher levels (*P* = 0.004) in the skin of the body of blue fish compared to red fish but not significantly different in the fins (fig. S18A). *alkal2l* encodes a ligand of leukocyte tyrosine kinase (*37*), which, in zebrafish, is necessary for the development of iridophores, the chromatophores that generate refractive colors such as blue (*37*, *38*). Together, this suggests that genetic variation affecting this developmental cell signaling ligand affects the extent of ornamental blue coloration. *alkal2l* likely corresponds to the gene referred to by betta breeders as the *spread iridocyte* gene, hypothesized to increase the prevalence of iridescence throughout the body (*46*).

Notably, the *alkal2l*-*bco1l* locus also modulated the red hue of the red parts of the body (Fig. 4, E and G). This suggested that *bco1l*, which encodes a protein predicted to metabolize orange-red carotenoids and whose homologs modulate coloration in other animals (*47*, *48*), could also be involved in differences between red and blue fish. Through biochemical assays, we found that, as predicted by its sequence homology to other BCO1 proteins, BCO1L has 15,15′-dioxygenase activity that cleaves β-carotene into two molecules of all-trans retinal (fig. S19, A to D). Consistent with the QTL effect on red hue and BCO1L biochemical activity, we found that red fish have more β-carotene and echinenone in their skin than blue fish (Fig. 4D). One of the *bco1l* variants most strongly associated with red and blue coloration results in a change from threonine in red fish to isoleucine in blue fish (figs. S16B and S19, E and F). We did not detect differential biochemical activity of the two alleles in vitro, but it is possible that their activity or stability differs in vivo, as there are no significant differences in skin *bco1l* mRNA levels between red and blue fish (fig. S19H). Therefore, variation in the locus containing *alkal2l* and *bco1l* likely affects both blue and red coloration through these two genes located only ~50 kb apart. The tight linkage might explain why breeders struggle to make the “perfect” red fish without any iridescence.

The second strongest GWAS peak, on chromosome 8, mapped to *adenylosuccinate lyase* (*adsl*), and the strongest QTL at this locus was for the brightness of blue areas on the body (Fig. 4, E and I). *adsl* encodes an enzyme involved in the de novo synthesis of purines. Purines are the major components of the reflective platelets in fish skin iridophores that underlie iridescence, and these platelets differ in structure between blue and red betta fish (*49*). While the homologs of *adsl* have not been previously implicated in animal coloration, mutations in other genes in the de novo purine synthesis pathway cause iridophore defects in zebrafish (*50*). Consistent with a role for *adsl* in blue coloration, we found that *adsl* is expressed in the skin of blue fish in cells abutting melanophores, where betta iridophores are located (fig. S18B) (*49*).

The third strongest GWAS peak, on chromosome 1, mapped to *solute carrier family 2, member 15b* (*slc2a15b*), a gene necessary for the development of larval yellow xanthophores in medaka (*51*) but whose role in adult pigmentation was previously not described (Fig. 4, A and J). We found a QTL that overlaps *slc2a15b* that strongly affected the saturation of red areas in the fins but not of the body or the head (Fig. 4, E and F). Intense coloration on the fins relative to the body is a phenotype referred to by breeders as the “Cambodian” variety, and our results suggest that *slc2a15b* contributes to this phenotype.

The fourth strongest GWAS peak, on chromosome 6, mapped to *kit ligand* (*kitlga*), whose orthologs affect melanin pigmentation in other fish and in mammals (*52*) (Fig. 4, A and L). A QTL overlapping *kitlga* strongly modulated the proportion of black, blue, and red on the head and fins but less so on the body (Fig. 4, E and H). A black head, a phenotype we found that is linked to *kitlga*, is referred to by breeders as the *mask* trait (*7*). This QTL also modified the saturation of blue areas on the fins but not on the body and had minor effects on red saturation outside the head. Its comparatively stronger impact on blue saturation may be related to the tight histological association of iridophores and melanophores as a unit in betta skin (*49*).

Together, we found that red-blue variation in ornamental betta is linked to genetic polymorphisms near two genes encoding cell signaling ligands (*alkal2l* and *kitlga*), two enzymes (*bco1l*, which metabolizes pigments, and *adsl*, which produces material for reflective structures), and a membrane solute transporter (*slc2a15b*). Candidate genes that we identified might correspond to those inferred, but not molecularly identified, by betta geneticists beginning in the 1930s (*53*, *54*). Notably, all of these genes had anatomical specificity, and all but two were on separate chromosomes (Fig. 4A).

### Genetic bases of fin morphology in ornamental betta

**Fig. 5.**
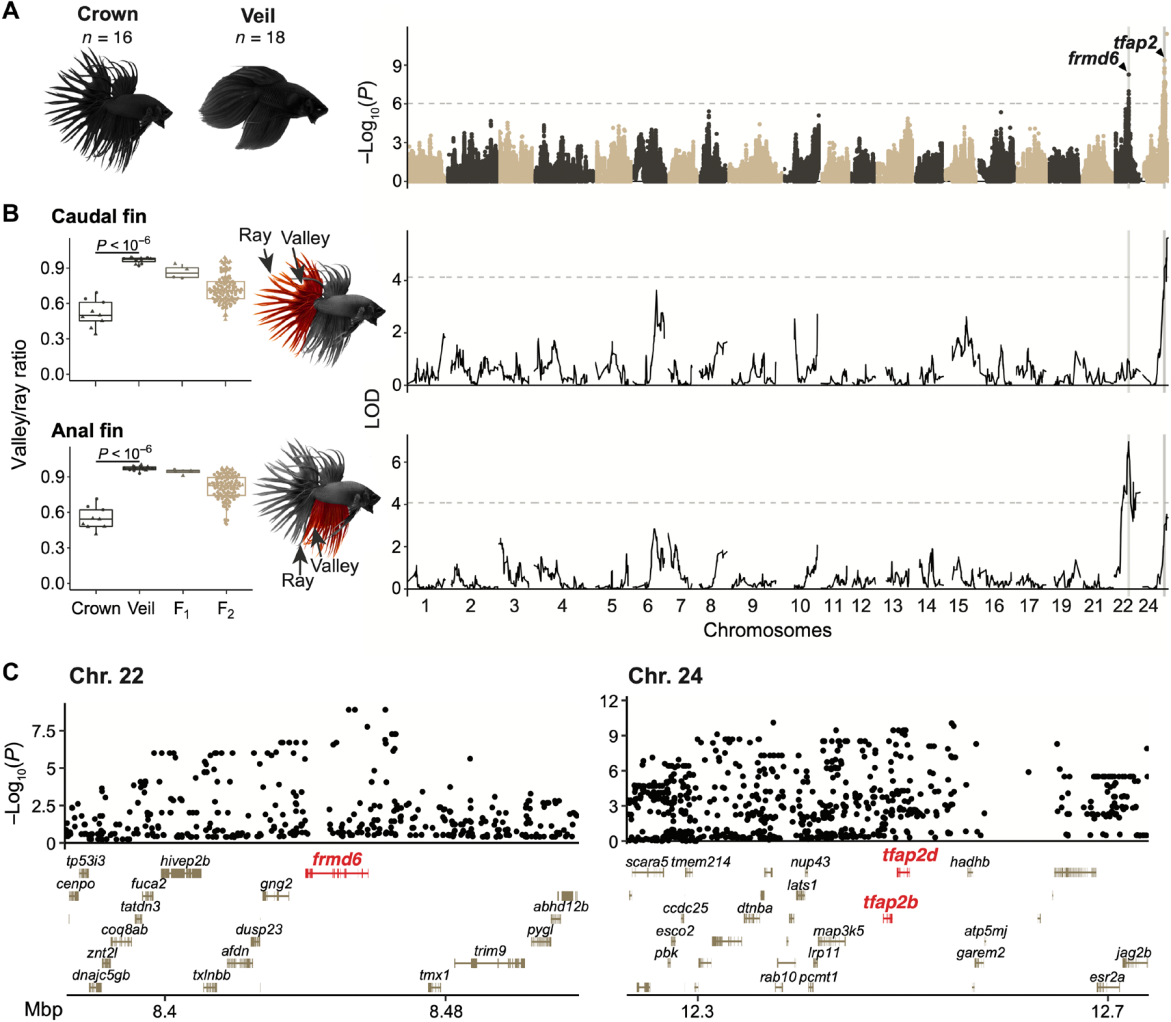
Genomic loci regulating fin morphology in ornamental betta. (**A**) Manhattan plot for GWAS of fin type between 18 veiltail and 16 crowntail ornamental betta. Dashed line denotes the genome-wide significance threshold. (**B**) Left: Valley/ray ratio in anal and caudal fins across crown (*n* = 9) and veil (*n* = 13) founder populations, F_1_ (*n* = 4), and F_2_ hybrids (*n* = 139). *P* value by Mann-Whitney *U* test. Right: QTL mapping of valley/ray ratios for caudal and anal fins. Dashed line denotes the genome-wide significance threshold (α = 0.05). (**C**) GWAS zoom-in across significant loci.

We found strong signals of selective sweeps in crowntail fish on chromosomes 22 and 24, suggesting that these regions could harbor variants associated with crown morphology (Fig. 2, D and E). To identify these variants within selective peaks and elsewhere throughout the genome, we performed a GWAS with the 18 veiltail and 16 crowntail fish used for the selection scans. We found two significant peaks, one on chromosome 22 and another on chromosome 24, overlapping the selection peaks (Fig. 5A), indicating that these regions are not only under selection but are the main loci contributing to differences between veiltail and crowntail fish.

To confirm the involvement of the GWAS loci in fin morphology, we performed a QTL mapping experiment in an F_2_ hybrid population from a cross between veil and crowntail fish (Fig. 5B). In agreement with the GWAS results, we found two significant QTLs, one on chromosome 22 and another on chromosome 24, that overlap the GWAS peaks. Unexpectedly, we found that the chromosome 22 locus is significantly linked only to anal fin webbing and not caudal fin webbing, whereas the chromosome 24 locus is linked to caudal fin webbing but not significantly linked to anal fin webbing (Fig. 5B). These complementary association and quantitative mapping experiments demonstrate that two loci are the primary determinants of veil-crown morphology and that webbing of different fins is under separate genetic control.

Examining the genes at the crown-veil GWAS peaks identified promising causal genes. The strongest association signal on chromosome 22 maps to *frmd6*, which encodes the protein willin that regulates tissue growth as part of the hippo pathway (Fig. 5, A and C, and fig. S20A) (*55*). The region of strongest association on chromosome 24 is larger and encompasses 22 genes (Fig. 5, A and C, and fig. S20A). Of these, *tfap2b* and *tfap2d*, which have evolutionarily ancient roles in ectodermal development (*56*), are prominent candidate genes. As with the variants that affect coloration, we also find evidence of anatomical modularity for the variants that affect fin morphology. These results also demonstrate that there is no single “crowntail gene,” as had been speculated by ornamental betta breeders (*7*).

## DISCUSSION

Using whole-genome sequencing of multiple *Betta* species, populations, and individuals, we take an important first step in unraveling the domestication history of betta fish. Our results suggest that betta were domesticated around 700 generations ago. While domesticated betta are largely derived from *B. splendens*, they carry genetic contributions from two other species that are also endemic to the Malay Peninsula: *B. imbellis* and *B. mahachaiensis*. None of the alleles derived from these other *Betta* species are present in all ornamental individuals, nor do they contribute to the regions under selection driving sex determination, coloration, or fin morphologies that we found here. These introgressed alleles, however, might contribute to other traits of domesticated betta or represent historical attempts by breeders to introduce new phenotypes into ornamental fish through hybridization.

The strongest genetic evidence of selection during domestication involves *dmrt1*, which we found is the sex determination gene in ornamental betta. Most females are XX, and most males are XY, settling a long-standing question in the field (*57*). A selective sweep of a *dmrt1_*X allele with increased penetrance may have been selected by breeders since it would lead to more predictable sex ratios in spawns. The lower penetrance of *dmrt1* for sex determination in wild *B. splendens* suggests that other sex determination mechanisms operate in the wild, similar to what is seen in zebrafish (*58*). In contrast to domesticated zebrafish, however, whose sex is not determined by a single locus, ornamental betta sex is predominantly determined by a single large-effect locus that maps to *dmrt1*.

In poeciliid fishes, such as guppies and swordtails, the sex determination locus is linked to multiple color genes that contribute to sexually dimorphic coloration and shape the genetics of female preference for male color traits (*59*). In contrast, the betta sex determination locus is only ~30 kb in size and is not linked to genes known to affect color. These results are consistent with coloration not being particularly sexually dimorphic in betta. Instead, we find that color and fin morphology in betta have a Lego-like logic, in which major-effect genes located on different chromosomes modulate color and fin morphology with unexpected anatomical specificity (table S2). Betta breeders are keenly aware of the mix-and-match possibilities of betta and leverage this feature to breed new fish varieties by combining different body, head, and fin colors with various fin morphologies.

Our results provide molecular entry points for further study of the developmental and evolutionary bases of change in morphology and sex determination. The genomic resources that we generated will also enable genetic studies into how centuries of artificial selection of betta for fighting purposes have shaped their aggression and other fighting-related traits. Together, our work elucidates the genomic consequences of the domestication of ornamental betta and helps establish this fish as a modern system for evolutionary genetic interrogation.

## MATERIALS AND METHODS

### Animal husbandry

Adult fish were housed individually in 1- to 1.4-liter tanks in a system with recirculating water with automated dosing using Instant Ocean sea salt and sodium bicarbonate to maintain a conductivity of 1.0 mS/cm and a pH of 7.0. Ten percent of the volume of the system is replaced daily with reverse osmosis–purified water. Water quality was monitored regularly for nitrates, nitrites, and ammonia. Water and room temperature are maintained at 28°C with a 14:10-hour light:dark cycle.

Larvae were grown from 5 to 14 dpf in 2.8-liter static tanks at a density of 30 larvae per tank and fed *Brachionus rotundiformis* rotifers. At 15 dpf, fry were moved to 37-liter grow-out tanks and fed two to four times daily with decapsulated newly hatched *Artemia nauplii*. Animal experimentation protocols were approved by the Columbia University Institutional Animal Care and Use Committee (AC-AAAT1482).

### Genome assembly

The fBetSpl5 assembly was created by assembling 48× PacBio CLR reads from SplBan0 with Falcon-unzip (*60*). Haplotigs representing retained duplication were removed using purge_haplotigs before scaffolding the assembly using Scaff10x (https://github.com/wtsi-hpag/Scaff10X) with 183× Illumina HiSeqX, 10X Chromium reads from a different individual (SplPhe0). Following scaffolding, the assembly was gap-filled with PBJelly (*61*) and polished with Arrow (https://github.com/PacificBiosciences/pbbioconda) using the long-reads data and then polished further using Freebayes (*62*) with 83× Illumina HiSeqX reads from SplBan0.

The resulting assembly was further improved with manual curation by building a gEVAL database and correcting mis-scaffolding by leveraging aligned Bionano consensus optical maps generated from SplPhe0 (*14*). Additional scaffolding was possible using haplotypic contig overlaps. The assembly was separated into chromosomes through alignment with the existing *B. splendens* assembly (GCA_003650155.1); these chromosomal units, including any discrepancies, were confirmed using the Bionano optical map data, presence of retained haplotypic contig overlaps, and general sequence contig integrity (expected read coverage, no read cliff). Chromosomes were named by synteny to medaka (*O. latipes*) assembly GCA_002234675.1 (fig. S1A and see the “Comparisons across reference assemblies” section in the Supplementary Materials). Curation resulted in 67 manual breaks, 92 manual joins, and the removal of 113 regions representing false duplications. The final assembly consists of 69 scaffolds totaling 441 Mb, with a scaffold N50 of 20.1 Mb. A percentage of 98.62% of the assembly has been assigned to the 21 chromosomes.

For RNA sequencing for genome annotation, we used fin, gonad, brain, liver, and gill from two adult wild *B. splendens*, SplChi0 and SplPhe30. Libraries of two different insert sizes (~350 and ~515 bp) were generated using the NEBNext Ultra Directional RNA Library Prep Kit for Illumina (E7420, New England Biolabs) and sequenced to an average coverage of 72 × 10^6^ reads (range: 33 × 10^6^ to 145 × 10^6^) with 2 × 150 bp reads in a NextSeq 500. Reads were used by NCBI to annotate the *B. splendens* genome in Annotation Release 101.

### Sample collection and resequencing

Sequencing libraries were generated from genomic DNA extracted from ethanol-preserved fin clips of wild samples and were sequenced on an Illumina platform, following Illumina’s polymerase chain reaction (PCR)–based library protocols. Whole-genome sequencing libraries for the ornamental samples were generated using the QIASeq FX DNA Library Kit (180473, Qiagen). Libraries were PCR-free with the exception of Orn30, Orn27, and Orn37, which were PCR-amplified in 6 cycles. Through receiver operating characteristic (ROC) curves of total variants called and the number of Mendelian errors in the trio and quartet sequenced to >30× coverage, we determined that 15× coverage was enough for our sample set and sequenced our ornamental *B. splendens* panel to ~15 ×.

Samples and the associated metadata included in the study are listed in table S1. Field sampling in Indonesia was conducted according to research permits 1/TKPIPA/FRP/SM/I/2011 and 3/TKPIPA/FRP/SM/III/2012 for L.R. Fieldwork in Peninsular Malaysia and Sarawak was conducted under permits issued by the Economic Planning Unit, Prime Minister’s Department, Malaysia (UPE 40/200/19/2417 and UPE 40/200/19/2534) and the Forest Department Sarawak (NCCD.970.4.4[V]-43).

### Alignment, variant detection, filtering, and phasing

We aligned DNA sequencing reads to the fBetSpl5.3 NCBI genome using bwa-mem2 (*63*) and marked duplicate reads using picard v2.0.1 (http://broadinstitute.github.io/picard/). We identified filter criteria for variants based on Mendelian errors from a trio and quartet using two variant callers: bcftools mpileup v1.11 (*64*) and GATK4 v4.1.4 (*65*). We assumed that de novo mutations are rare and that most Mendelian errors are sequencing artifacts and thus represent false positives. On the basis of ROC analysis, bcftools appeared to call more variants at similar false-positive rates compared to GATK. We determined that filtering out variants with mapping quality (MQ) of <50 and genotypes with genotype quality (GQ) of <30, depth (DP) of <4, and where the allele depth (AD) support for genotype was less than two reads provided the least number of Mendelian errors with the highest number of non-Mendelian errors through ROC of mpileup variant calls. We also excluded variants that are adjacent to each other within 3 bp. To increase the confidence of heterozygous genotypes, we further filtered out heterozygous genotypes in which the variant allele fraction (alt depth/DP; VAF) showed an allelic imbalance: VAF < 0.25 and VAF > 0.75.

For short (<100-bp) indel analysis, we used GATK4 for calling indels as it uses local realignment around indels and filtered with more stringent criteria including requiring indels to have GQ > 50, fraction missing < 0.2, and are present in more than one individual. These variants were required to be concordant with Mendelian expectations if present in either the trio or quartet, leaving 228,066 indels. For structural variant analysis (structural rearrangements, indels >100 bp), we used smoove (https://github.com/brentp/smoove), filtering out genotypes with GQ < 50 and for which deletions carried a DP > 0.7× of the flanking region (duphold flank fold-change, DHFFC > 0.7). We additionally filtered out duplications that were <1.3× relative to other similar GC-content bins across the genome (duphold bin fold-change, DHBFC < 1.3) and inversions/translocations with DP < 4. We manually inspected these variants through Integrated Genomics Viewer (*66*). We performed chi-square analysis corrected for false discovery rate to assess the association of variants near regions identified by genome-wide association and QTL analysis, and none were more associated than SNPs through analogous chi-square statistics.

#### Regions filter

We calculated read counts for each bam file across fBetSpl5.3 segmented into 1000-bp windows with a 500-bp slide. For each sample, we log_2_-normalized the read counts of each window by the median read count across the genome. For pop-genome analysis, we removed regions where read counts were either >2.5 SD from the median within a sample or where the variance of the log_2_-normalized read counts across the bam files was >2.5 SD from the median variance across the genome across all bam files. For association mapping analyses in ornamental betta, we applied the latter filter only to the ornamental betta samples to prevent removal of regions that were lost or gained in other species/populations but are stably diploid in ornamental betta.

To further filter out regions with poor mappability, we used SNPable (https://github.com/lh3/misc) with –l 200 and –r 0.5. Most regions (98.0%) that were masked through SNPable were also removed on the basis of our read-coverage filter (table S4).

#### Phasing

Using the filters described above, along with a fraction missing threshold of 0.2, we phased the genotypes in a two-step method. We performed read-based phasing of the quartet and trios with the pedigree phasing mode of WhatsHap v0.18 (*67*). We included the phase set generated by WhatsHap into ShapeIt4 v4.1.3 (*68*) to phase all samples with the recommended error rate (--use-PS 0.0001) and settings for sequencing data (--sequencing). To increase phasing accuracy, we increased the number of mcmc iterations “--mcmc-iterations 10b,1p,1b,1p,1b,1p,1b,1p,10m” and the number of conditioning haplotypes “--pbwt-depth 8.”

### Species phylogenies

On the basis of the filtered phased biallelic SNP callset, we calculated pairwise differences across individuals using plink v1.90p (*69*) with the option --distance square both for the whole genome and in 100-kb windows. NJ trees were calculated with the nj function of Python’s skbio package v0.5.6 (http://scikit-bio.org/). Block bootstrap support was determined by randomly subsampling window-pairwise differences with replacement and testing how often a node of the whole genome tree was supported in 1000 random replicates. Maximum likelihood trees were generated using IQ-TREE v2.0.3 (*70*) with model HKY+F+R2 based on ModelFinder and then bootstrapped (-B).

### Gene flow analyses

#### ABBA-BABA analysis

We computed ABBA-BABA tests for all triplets of samples with Dsuite v0.4 r38 (*19*), defining each individual as a separate population and running Dsuite Dtrios with default parameters. Unless otherwise stated, we used the *B. compuncta* sample as the outgroup. All f4 ratio plots show f4(p1,p2,p3,outgroup) for f4(p1, p2, p3, outgroup) > 0 and -f4(p2, p1, p3, outgroup) otherwise.

#### f_dM_ analysis in ornamental betta

Windowed f_dM_ was computed across the genome with Dsuite Dinvestigate using a window size of 100 SNPs with a step of 25 SNPs for each of the triplets of samples that showed signs of introgression with the ABBA-BABA tests. For each triplet, we smoothed the f_dM_ along the chromosome using a MAD winsorization (*71*), so that a single window outlier inconsistent with the f_dM_ values observed in adjacent windows would not affect downstream segmentation. We then applied a piecewise segmentation across the chromosomes and calculated the mean f_dM_ of each segment. Across all sample triplets, we observed a clear bimodal distribution in f_dM_ values of these segments with one mode at 0.01 and another above a 0.2 valley (fig. S9C). We thus filtered for segments with f_dM_ > 0.2 and merged adjacent remaining segments that were within 5 kb of each other to determine the start and end points of the introgressed regions. Local gene trees were constructed for each of these high-f_dM_ genomic regions using IQ-TREE across the haplotypes of the ornamental bettas and other *B. splendens* species complex samples that did not show recent introgression through the ABBA-BABA tests. For each region, we determined the ancestry of each haplotype for the ornamental sample (p2) with high f_dM_ by calculating the average branch length distance of the sample’s haplotype to the haplotypes of other samples for each species. If the average distance of the p2 sample’s haplotype to the haplotypes of a non-*splendens* species was shorter than the average distance to other *B. splendens* haplotypes and less than 2 SD away from the average distance between *B. splendens* haplotypes, we determined that the haplotype likely shared ancestry with that non-*splendens* species. We further validated ancestry assignments by visual inspection of the trees, all of which showed the topology inferred by these analyses (fig. S9, D and E).

### Demographic inference

We used Relate v1.1.7 (*23*) to infer the demographic histories of the wild *B. splendens* populations and ornamental betta. We generated an ancestral sequence reconstruction with IQ-TREE v2.0.3 using the phased variant call set from *B. compuncta*, *B. smaragdina*, *B. mahachaiensis* (only Mah0 sample because it had no evidence of introgression from *B. splendens*), and wild *B. splendens*. We did not include ornamental betta as many samples have introgressed regions from other species. We constructed trees for each chromosome using the HKY+F+R2 model with 1000 bootstraps (-bnni). Tree topologies supported the species tree (Fig. 1B and fig. S3A) and did not differ across chromosomes. We then used IQ-TREE (-asr) to infer the ancestral state sequence at the node supporting the split between Mah0 and *splendens*. We assigned the allele with the highest posterior probability as the ancestral state at each biallelic variant position. Positions where no nucleotide had a posterior probability above 0.9 (which occurred in 0.001 of positions) were assigned the International Union of Pure and Applied Chemistry ambiguity code “N” as the ancestral state.

We used a constant recombination rate based on the genetic length of each chromosome (as determined from the genetic map of an F_2_ intercross; see the “QTL analysis of color” section below). The genome-wide average rate was 6.6 centimorgan (cM)/Mb. We ran Relate using our point estimate for the de novo mutation rate and the upper and lower CI boundaries. We assumed a diploid starting effective population size of 91,300 based on the nucleotide diversity of 0.00137 of wild *B. splendens* from Kanchanaburi and the point estimate of the mutation rate. Inferred demographies were similar when using a starting population size of 37,000 or of 378,000, based on the CI from the mutation rate (fig. S10D). We excluded the following samples with signs of recent introgression or that are the offspring of the quartet or trio: Orn29, Orn21, Orn5, Orn38, Orn39, SplKan8, SplPhe2, SplBan0, and SplBan1. We further excluded all genomic regions with evidence of non–*B. splendens* introgression in the included samples (amounting to 11% of the genome) and found negligible differences in the demographies inferred by Relate (fig. S10B). We ran the Relate EstimatePopulationSize algorithm for one iteration to avoid introducing biases generated from the assumption that our samples belong to one panmictic population. We performed jackknife resampling of chromosomes and observed negligible variation when removing individual chromosomes. We also performed 100 runs of 5-kb block bootstrap resampling across chromosome 1 to assess the variability of inferred demographics (fig. S10C).

### Bottleneck inference

We extracted the site frequency spectrum of genomic regions without evidence for recent admixture as described in the “Gene flow analyses” section for ornamental and Kanchanaburi samples from the filtered, phased, and biallelic SNP VCF file. Strongly admixed individuals Orn5, Orn21, and SplKan8, as well as the offspring from trios, were removed from the analysis. This yielded an accessible genome of 300 Mb and a total of 0.704 million and 1.02 million SNPs for ornamental and Kanchanaburi samples, respectively. The site frequency spectrum was polarized using the ancestral state inferred as described in the “Demographic inference” section.

We used the site frequency spectra as input to fastsimcoal2 (fsc2 v2.7.0.2) (*24*) inferring the time and intensity of a single instantaneous bottleneck (*25*) within the last 3000 generations and an otherwise constant effective population size using the instbot feature. We focused on such a simple model to specifically look for evidence for a recent, strong domestication bottleneck rather than trying to fit the full, undoubtedly complicated population history (which is explored using Relate in the “Demographic inference” section).

To check that such a simple model can accurately time a relatively recent bottleneck in a variety of “background” demographic histories, we used msprime v1.0.1 (*72*) to simulate bottlenecks of different strengths and timings assuming different demographic histories. We simulated 35 diploid ornamental genomes (same as our real sample size) consisting of 21 chromosomes of length 14 Mb (the average *B. splendens* chromosome length). First, we assumed constant effective population sizes of 10,000 or 100,000 individuals. Second, we ran simulations following the inferred Relate demographic history for times older than 1000 generations ago. Since, given our sample size, Relate results for more recent times are less reliable (fig. S10B), we assumed linear growth toward the inferred long-term effective population size of 72,000. On top of these demographic models, we added bottlenecks of different timings and intensities. We added a single instantaneous bottleneck in the past 100, 250, 500, 1000, and 2000 generations ago of varying bottleneck intensities (0.2, 0.4, 0.6, and 0.8; msprime strength = 2Ne*fsc2 intensity) and a demography without a bottleneck. A constant recombination rate of 6.6 cM/Mb and mutation rate of 3.75 × 10^−9^/bp per generation based on our point estimate of the de novo mutation rate were applied. For each parameter combination, we ran five independent replicates of the fsc2 instbot model and one “null model” in which the bottleneck intensity was fixed to zero (fig. S10E).

### Diversity statistics

We calculated genome-wide nucleotide diversity (pi) in ornamental and wild *B. splendens*, accounting for accessible sites after filtering using the *B. splendens* regions filter and filtering out regions with evidence of introgression from the f_dM_ analysis. We removed from the analysis the following samples from the wild populations because they had signs of introgression: SplKan8, SplPhe2, SplBan0, and SplBan1. Nucleotide diversity was calculated per site using the python package scikit-allel (https://github.com/cggh/scikit-allel), and the sum is divided by the total accessible (invariant and variant) sites remaining after filtering. CIs were calculated on the basis of jackknife resampling of chromosomes. We calculated windowed nucleotide diversity in 10-kb windows with 100-bp slide across the genome (fig. S12). We removed windows where 3 kb or less of the window was accessible.

### LD analysis

We assessed LD using plink v1.90, calculating the *r*^2^ value of SNPs with a minor allele frequency (MAF) of 0.2 (in either ornamental betta or wild *B. splendens* from Kanchanaburi) and --ld-window-kb 999. To assess LD decay, we generated bins that incrementally increased by 3 bp from 0 to 999 kb and calculated the mean *r*^2^ value of SNPs where the distance between the SNPs fell within each bin. Calculations of interchromosomal *r*^2^ value after thinning SNPs that were within 100 kb of each other within a chromosome (--bp-space 100000) served as the baseline using --r2 interchrom and --ld-window-r2 0.

### Selection scans

We performed three types of genome-wide scans to identify signatures of selection: (i) *H*-scan (*26*) to detect extended homozygosity tracts, (ii) G12 and G2/G1 scans with 200 SNP windows (*73*) to assess haplotype frequency spectra across the genome and to identify hard and soft sweeps across populations, and (iii) Tajima’s *D* in 10-kb windows with 100-bp slide across the genome using scikit-allel. To determine whether differences in *H* between groups of fish (red and blue, crowntail and veiltail, and male and female) were greater than expected by chance, we performed permutation testing. As neighboring loci may share high *H*-scan values due to linkage, representing the same putative sweep (*74*), we clumped loci that were part of the same LD block. For each chromosome, we pooled the *H* values of the compared groups and then randomly sampled from this pool to assign scan values across the chromosome for each group. We calculated the absolute difference in *H* values between the two groups across regions of the chromosomes. We performed 1000 permutations for each chromosome and then calculated the 99% percentile (α = 0.01) across the distribution of differences of all permutations across chromosomes, which served as our genome-wide threshold.

### Genome-wide association analysis

Genome-wide association analysis for sex, color, and fin morphology across ornamental betta was performed using GEMMA version 0.98.1, with a linear mixed model that incorporated the kinship/relatedness matrix to account for population structure (*75*); see Q-Q plots in fig. S15. We removed variants with MAF < 0.05, fraction missing < 0.2, and *r*^2^ > 0.8. We performed genome-wide association using biallelic variants for sex (confirmed by inspection of gonads after euthanasia) across 37 ornamentals (females, 17; males, 20). For color, we performed genome-wide association across 34 ornamentals (red, 17; blue, 17). For fin morphology, we performed genome-wide association across 34 ornamentals (veil, 18; crown, 16).

We considered haplotype blocks to represent the number of independent statistical tests. We calculated the number of haplotype blocks using plink --blocks with a maximum kilobase window of 999 and MAF of 0.05. We identified 53,844 haploblocks in ornamental betta. Thus, for a Bonferroni-corrected *P* value of 0.05, we set the significance threshold in GWAS as −log_10_(0.05/53844) = 6.03.

We also performed a k-mer association analysis using HAWK v1.3 (*76*) for sex in ornamental betta. We assembled the k-mers into contigs using ABYSS v2.0 (*77*) and mapped these sequences to the fBetSpl5.3 genome using bwa-mem. All significantly associated k-mers could be successfully mapped to the reference.

### QTL analysis of sex

We performed an F_2_ intercross between a male (*dmrt1_XY*) and a female (*dmrt1_XX*) ornamental splendens. We crossed the F_1_ (*dmrt1_XY*) males and F_1_ (*dmrt1_XX*) females to generate 211 F_2_ progeny. We sexed each F_2_ based on the presence of an ovipositor and confirmed after euthanasia by the presence of ovaries or testis. We generated Tn5-tagmented whole-genome sequencing libraries (*78*) and sequenced the samples to a depth of ~0.05× in a NextSeq 500/550 (75 cycles). We generated a panel of SNPs where the two founders differed in their genotype. In addition to the filters described in the “Alignment, variant detection, filtering, and phasing” section, we filtered out variants with DP greater than or less than 1.5 SD of the median DP on a per-sample basis and removed variants that were within 150 bp from each other (read length). We excluded SNPs that were private to a founder and were not present across the ornamentals in the GWAS cohort. We also excluded variants with non-Mendelian segregation in our trio and quartet. For the F_2_s, we counted the allele read depth for each SNP using alleleCount (https://github.com/cancerit/alleleCount) with base quality > 20 and MQ > 35.

To quality-prune the SNPs used for imputation, we excluded SNPs where fewer than 10% of individuals had read coverage. We then performed imputation using AncestryHMM v0.94 (*79*) for an F_2_ intercross design. We removed SNPs where the genotype likelihood was less than 90% across more than 50% of the samples. We additionally removed SNPs where the difference of the posterior probability of the SNP relative to the preceding SNP was less than 10%. Using the remaining 15,572 SNPs, we performed QTL analysis for sex using Haley-Knott regression in R/qtl (*80*). We performed nonparametric interval mapping and determined significance thresholds by 1000 permutations of the data using scanone.

To investigate whether a secondary sex determination system exists for males carrying the *dmrt1_XX* haplotype, we also performed an F_2_ intercross between a male (*dmrt1_XX*) and female (*dmrt1_XX*) ornamental betta, resulting in 100 F_2_s. None of the founders of these crosses were close relatives of each other. Following the same filtering criteria, we performed QTL analysis for sex (confirmed gonadally) on 8561 SNPs. To minimize environmental biases, we raised the *dmrt1*_*XY* male × *dmrt1*_*XX* female and *dmrt1*_*XX* male × *dmrt1*_*XX* female crosses at the same time using the same housing, water, and temperature conditions.

### XY haplotype analysis

To generate a haplotype tree of the *dmrt1* locus, we used the filtered, phased, and biallelic SNP variants within the LD block of chromosome 9 spanning 28,850,248 to 28,884,157 bp. We included both invariant and variant sites within the region. We excluded the following samples due to signs of recent gene flow, due to being the offspring of the quartet or trio, or due to being sexually immature: Orn29, Orn5, Orn38, Orn39, SplPhe1, SplPhe2, SplBan0, SplBan1, SplChi1, SplChi2, and SplKan6. We used IQ-TREE v2.0.3 (*70*) with the recommended model “HKY+F+R2” with 1000 bootstraps, optimizing the bootstrap tree with nearest-neighbor interchange (-bnni). We collapsed branches with bootstrap support of <80% and used this tree to infer ancestral sequence at each node and tip of the tree (-asr).

### Genotyping

#### Sequencing-based genotyping

To validate the sex GWAS results, we generated an independent panel unrelated to the GWAS sample set consisting of 161 unrelated ornamentals (females, 83; males, 78). We performed a local chi-square analysis of the regions carrying the highest associated GWAS SNPs for sex in the ornamentals to select the SNPs most associated with sex and present in the Y branch. We PCR-amplified and Illumina-indexed products spanning these SNPs (tables S3 and S4) and sequenced the amplicons using NextSeq 500/550. We removed aligned reads that had an MQ of less than 10 and then performed bcftools mpileup with -C 50. We removed genotypes with DP < 50, GQ < 30, and where the SNP VAF was between 0.2 and 0.3 or 0.7 and 0.8 from further analysis.

#### Restriction fragment length polymorphism–based genotyping for sex

We developed a diagnostic restriction fragment length polymorphism (RFLP)–based assay for genotyping *dmrt1_XX* versus *dmrt1_XY*. We identified an SNP with *r*^2^ > 0.8 with the top 0.5% of the highest associated GWAS SNPs for sex that overlapped the restriction enzyme site for MluCI. We PCR-amplified the region and overnight digested the amplicons with MluCI (R0538L, NEB) (tables S3 and S4). The PCR fragments were then visualized on a 2.5% agarose gel (fig. S21). To confirm the accuracy of the RFLP assay, we Sanger-sequenced the undigested PCR amplicons of five *dmrt1_XY* samples and five *dmrt1_XX* samples and found perfect concordance.

### QTL analysis of color

We generated an intercross between a red (*dmrt1_XY*) male and a blue (*dmrt1_XX*) female ornamental betta, crossed two pairs of F_1_ hybrids, and generated 211 F_2_ progeny (one of the crosses used in QTL mapping of sex). We genotyped F_2_s as described in the “QTL analysis of sex” section and then performed Haley-Knott regression in R/qtl.

#### Imaging

We photographed both sides and top of sexually mature F_2_s in a plastic tank inside a photo studio tent lined with white light-emitting diodes using a Canon EOS RP camera with a Canon Macro lens EF 100 mm. There were minimal light distortions when assessed with a polarized lens. At the start of each photo session, we calibrated the camera using the white balance (CT24-23-1424) provided on the 24ColorCard from CameraTrax.com. Each image included the color checker card.

#### Color processing analysis

We used a custom python script to automatically identify the color matrix and calibrated each picture based on the reference color values on the color checker card with a pipeline built on python packages, rawpy, colour, and PlantCV. We segmented images using a recursive-convolutional neural net model across 122 images that were annotated and trained in MATLAB 2019b. Images that were poorly segmented were manually segmented with ImageJ for downstream analysis. Downstream analysis was performed using a custom R script based on imager (https://dahtah.github.io/imager/). Color analysis was based on the hue-saturation-value color space, using the hue range [0,0.045], [0.98,1] for red and [0.6,0.728] for blue. These ranges maximized the difference between the red (*n* = 17) and blue (*n* = 17) ornamental betta through iterative subtractive binning. We similarly determined the range [0 to 0.3] to assess blackness based on the value axis along the color space.

### QTL analysis of fin morphology

We generated an F_2_ intercross consisting of 139 F_2_ progeny from a veiltail (*dmrt1_XX*) male with a crowntail (*dmrt1_XX*) female ornamental betta (one of the crosses used in QTL mapping of sex; the additional 39 F_2_s were not raised concomitantly with the rest and excluded from analyses of sex determination to minimize environmental confounders). We pinned sexually mature euthanized fish to a sylgard-coated petri dish with the fins expanded and photographed them as described in the “QTL analysis of color” section. Using Fiji, we annotated the length of the ray and webbings between the primary rays measured from the base of the tail and. We genotyped F_2_s as described in the “QTL analysis of sex” section and performed Haley-Knott regression using R/qtl.

## Supplementary Materials

### This PDF file includes:

Supplementary Notes S1 to S6 Supplementary Methods Figs. S1 to S21 Tables S2 to S4 References

## Notes

View/request a protocol for this paper from *Bio-protocol*.
