# Supplementary material for: Genomic consequences of domestication of the Siamese fighting fish
Source: Sci Adv. 2022 Mar 9;8(10):eabm4950. doi: 10.1126/sciadv.abm4950 (PMC8906746; doi:10.1126/sciadv.abm4950)
Supplement: Supplementary file 1 — Supplementary Notes S1 to S6 Supplementary Methods Figs. S1 to S21 Tables S2 to S4 References [file sciadv.abm4950_sm.pdf]

Science Advances
AAAS

Supplementary Materials for

Genomic consequences of domestication of the Siamese fighting fish

Young Mi Kwon, Nathan Vranken, Carla Hoge, Madison R. Lichak, Amy L. Norovich, Kerel X. Francis, Julia Camacho-Garcia, Iliana Bista, Jonathan Wood, Shane McCarthy, William Chow, Heok Hui Tan, Kerstin Howe, Sepalika Bandara, Johannes von Lintig, Lukas Rüber, Richard Durbin*, Hannes Svardal*, Andres Bendesky*

*Corresponding author. Email: rd109@cam.ac.uk (R.D.); hannes.svardal@uantwerpen.be (H.S.); a.bendesky@columbia.edu (A.B.)

Published 9 March 2022, *Sci. Adv.* **8**, eabm4950 (2022)
DOI: 10.1126/sciadv.abm4950

The PDF file includes:

Supplementary Notes S1 to S6
Supplementary Methods
Figs. S1 to S21
Legend for table S1
Tables S2 to S4
References

Other Supplementary Material for this manuscript includes the following:

Table S1

Supplementary Notes

1. Synteny between wild *B. splendens* and ornamental betta

To discover structural rearrangements that may have arisen during domestication, we performed whole genome alignments using three ornamental betta references (*11–13*) and our wild *B. splendens* reference. An ornamental genome assembled using HiC is largely syntenic with wild *B. splendens (11)* (fig. S1B). The largest differences are a translocation of a portion of chromosome 14 in the wild genome to chromosome 12 in ornamental and a large intrachromosomal rearrangement of chromosome 16, with breakpoints between 5,230,426-5,270,524 and between 15,297,273-15,388,532. The translocation of wild chromosome 14 was not observed in the comparison to the other two ornamental references, which were assembled using Oxford Nanopore sequencing (*12*) (fig. S1C) or Illumina short reads (*13*) (fig. S1D), suggesting this translocation is not present in all ornamental betta, or reflects an incorrect assembly of the ornamental HiC reference. In contrast, the rearrangement of chromosome 16 was also observed in the ornamental Nanopore reference and possibly in the Illumina reference (fig. S1C,D). More evidence for a translocation in chromosome 16 is observed in the patterns of recombination of an experimental ornamental cross (see Methods: Quantitative Trait Locus analysis), mapped to the wild fBetSpl5.3 reference, in which a portion in the beginning of the chromosome appears linked to the end of the chromosome (fig. S1F). This rearrangement may have occurred in the lineage that gave rise to these domesticated betta, or is specific to the wild *B. splendens* used for the fBetSpl5.3 reference or its lineage. To distinguish between these possibilities, we aligned the wild *B. splendens* genome to *Anabas testudineus (16)*, an anabantoid fish that is an outgroup to the *Betta* genus, and found that their chromosomes 16 were largely syntenic (fig. S1E). Thus, the most parsimonious explanation is that the chromosome 16 rearrangement occurred during domestication. The Nanopore assembly has many putative rearrangements compared to wild *B. splendens*, but these are also observed when aligning the Nanopore reference to the HiC ornamental reference, suggesting that the Nanopore assembly is not as well assembled. These results indicate that the genomes of ornamental and wild *B. splendens* are largely syntenic, except for a potential large rearrangement of a single chromosome.

2. Evolutionary relationships between species of the *B. splendens* complex

There are strong and highly significant signals of excess allele sharing among most species of the *B. splendens* species complex (fig. S4A). These signals are particularly striking for *B. mahachaiensis*, where excess allele sharing with other groups varies strongly among the three samples. For example, while Mah2 and Mah1 show very strong excess allele sharing with wild *B. splendens* samples compared to *B. siamorientalis* or *B. imbellis* (median f4-admixture ratios f4(*B. siamorientalis*, *B. splendens*; *B. mahachaiensis*, *B. compuncta*) = 28.5% and 19.8%, respectively; block jackknifing $P<10^{-300}$) (fig. S4A), Mah0 shows the opposite pattern (median f4-admixture ratio f4(*B. splendens, B. siamorientalis*; *B. mahachaiensis*, *B. compuncta*) = 5.6%; block jackknifing $P<10^{-300}$) (fig. S4A). This pattern is highly suggestive of varying levels of introgression into these *B. mahachaiensis* samples.

For *B. imbellis*, sample Imb1 from the western Malay Peninsula was ~4.7% closer to *B. splendens* than was Imb0 from the eastern Malay Peninsula (median f4(Imb0, Imb1; *B. splendens*, *B. compuncta*)=0.047, block jackknifing $P<10^{-300}$) (fig. S4B). This difference between Imb0 and Imb1 was equally strong across all sampled *B. splendens* populations (fig. S4C), suggesting genetic introgression into *B. imbellis* populations of the western Malay Peninsula from a *B. splendens* lineage ancestral to, or separate from, the populations that we sampled.

3. Evolutionary relationships among *B. splendens* populations

Investigating the relationships between wild *B. splendens* populations, pairwise genetic differences, patterns of derived allele sharing (BBAA patterns from ABBA-BABA analysis) and phylogenetic clustering, give a consistent picture with the populations from Chiang Mai and Phetchaburi clearly closest, Kanchanaburi a sister group to them and Bang Phlat as an outgroup (fig. S3; 5A). Strong signals of excess allele sharing also suggest a complex divergence history. Most strikingly, while Kanchanaburi show moderate, mostly non-significant excess allele sharing with Bang Phlat compared to two Phetchaburi samples, the pattern is reversed for the third Phetchaburi sample, SplPhe2, which shows highly significant excess allele sharing with Bang Phlat compared to Kanchanaburi (median f4(Kanchanaburi, SplPhe2, Bang Phlat, outgroup) = 4.5%, median $P=2x10^{-7}$, fig. S5B). This observation suggests significant amounts of genetic material from a population related to Bang Phlat in SplPhe2.

Furthermore, individuals from Kanchanaburi are significantly closer to Bang Phlat relative to individuals from Chiang Mai (median f4(Chiang Mai, Kanchanaburi; Bang Phlat, outgroup)= 1.9%, median $P=0.003$, fig. S5B). However, comparisons involving SplKan8 are outliers in this statistic, showing a much stronger signal (median f4(Chiang Mai, SplKan8; Bang Phlat, Outgroup)= 4.5%, median $P=10^{-8}$; fig. S5B).

Finally, individuals from Phetchaburi tend to be closer to individuals from Kanchanaburi compared to individuals from Chiang Mai (median f4(Chiang Mai, Phetchaburi; Kanchanaburi, outgroup)= 1.6%, median $P=0.003$), and individuals from Kanchanaburi are closer to Bang Phlat relative to Chiang Mai (fig. S5B). This trend tends to be more extreme for comparisons involving SplPhe2 (median f4(Chiang Mai, SplPhe2; Kanchanaburi, outgroup)= 2.2%, median $P=0.0007$) and clearly is most extreme for comparisons involving both SplPhe2 and SplKan8 (median f4(Chiang Mai, SplPhe2; Kanchanaburi, Outgroup)= 4.7%, median $P=10^{-9}$, fig. S5B). Together, these observations suggest some shared ancestry between SplPhe2, SplKan8 and the two Bang Phlat samples. In note S4, we show that this pattern is explained by ornamental betta ancestry in these samples, likely due to introgression.

4. Excess allele sharing of ornamental betta with non-*splendens* species

We tested for excess allele sharing between ornamental betta and non-*splendens* with ABBA-BABA tests of the form *D*(wild *splendens*, ornamental; non-*splendens* species, outgroup). The outcome of these tests varied substantially by the population of origin of the wild *B. splendens*

considered, pointing to a complex divergence history within *B. splendens*. This variation made these tests ill-suited to draw definite conclusions on non-*splendens* contributions to ornamental betta.

To address this issue, we focused on comparisons of the form *D*(ornamental except focal, focal ornamental; non-*splendens* species, outgroup) (fig. S5G-J). We removed *B. mahachaiensis* individuals Mah1 and Mah2 from the analysis as they carry ornamental betta introgression (fig. S5L), which could bias inferences about introgression into ornamental betta. The few genomic regions of excess allele sharing of ornamental betta with *B. siamorientalis* and *B. smaragdina* correspond to gene trees where a haplotype of the focal sample clusters with *B. imbellis* and *B. mahachaiensis*, respectively, suggesting that these regions are less phylogenetically distinct and suggest these signals also correspond to introgression from *B. imbellis* and *B. mahachaiensis*. Indeed, excess allele sharing with *B. siamorientalis* is expected under *B. imbellis* introgression, given their sister-species relationship, and *B. smaragdina* excess allele sharing in samples with *B. mahachaiensis* introgression can be explained by allele sharing between *B. mahachaiensis* and *B. smaragdina* suggestive of gene flow between these species (fig. S4A).

To test whether the excess allele sharing of some ornamental betta with non-*splendens* species is also observed for wild *B. splendens*, we performed the same analysis as in fig. S5G-J for wild *B. splendens*. We computed for each focal wild *B. splendens* sample, *D*(same wild population as focal except focal, focal, non-*splendens* species, outgroup) and calculated the number of significant tests (i.e., below Bonferroni corrected $P<0.01$, corresponding to a z-score of 5.49) (fig. S5K). We removed two wild splendens outlier individuals SplKan8 and SplPhe2 which carry ornamental betta ancestry (fig. S6A). We found that wild *B. splendens* generally show no significant excess allele sharing with non-*splendens* species compared to other samples of the same population. The only clear exception to this are the two Bang Phlat samples. SplBan1 is consistently closer to other species than SplBan0 in all comparisons involving SplBan0 and SplBan1 as p1 and p2, respectively (fig. S6B,C). Since our reference assembly is based on SplBan0, we hypothesized that the observed pattern could be caused by the fact that sequencing reads for which the outgroup and the reference genome differ (corresponding to BXXA patterns) are less likely to align for relatively a divergent outgroup, leading to an excess of ABBA compared to BABA patterns. To test this we recomputed ABBA-BABA tests using the more closely related *B. smaragdina* as the outgroup instead of *B. compuncta*. With *B. smaragdina* as an outgroup, there is no differential allele sharing of SplBan0 and SplBan1 with other groups (fig. S6C, right panel), while the excess allele sharing results of ornamental samples still hold up (fig. S5G-J). In conclusion, significant excess allele sharing with non-splendens species is generally not observed in wild *B. splendens* populations but it is common among ornamental betta, suggesting that many of them carry significant (but generally small amounts) of non-*splendens* introgression.

5. Ornamental betta introgression is widespread among wild *Betta*

One striking observation in the pairwise-distance based NJ trees presented above is that, despite high bootstrap support, the topology of relationships among wild *B. splendens* populations changes once ornamental samples are added (fig. S3A,C). In both trees, the samples from Chiang

Mai and Phetchaburi are clearly closest to each other. However, without the ornamental samples, these populations form a sister-group to samples from Kanchanaburi with samples from Bang Phlat as a further outgroup. Conversely, in the tree including ornamentals, Chiang Mai and Phetchaburi cluster with ornamentals and Kanchanaburi and Bang Phlat together form a sister group to this. Furthermore, in the tree including ornamentals, one Phetchaburi sample changes its placement, now forming an outgroup to Chiang Mai and other Phetchaburi samples.

To further investigate this, we computed all ABBA-BABA statistics consistent with the phylogeny (including ornamentals) (fig. S3C). This analysis revealed that the above-mentioned Phetchaburi sample, SplPhe2, carries a substantial amount of ornamental betta ancestry (median f4(Chiang Mai, SplPhe2; ornamental, outgroup) ~22%, (fig. S6A). The two other Phetchaburi samples also have highly significant, but much weaker excess allele sharing with ornamental betta (median f4(Chiang Mai, other Phetchaburi; ornamental, outgroup) ~4%), suggesting that these samples might also have some ornamental betta introgression. Investigating the patterns of excess allele sharing along the chromosomes using f_{dM} revealed that these patterns are widely distributed across chromosomes, without any apparent large outlier regions (fig. S7A). This suggests that ornamental introgression into the Phetchaburi population is not recent, leaving time for recombination to break down haplotypes.

Furthermore, Bang Phlat individuals are significantly closer to ornamental betta relative to Kanchanaburi samples (median f4 ratio = 4.0%, median $P=10^{-4}$; fig. S6A,B), consistent with genetic exchange between Bang Phlat individuals and a lineage related to our ornamental betta samples. Because Bang Phlat is located within Bangkok, ornamental introgression into the Bang Phlat population is plausible. An exception to the above pattern are comparisons involving SplKan8, which is closer to ornamentals compared to Bang Phlat (median f4 ratio = 3.2%, median P=0.004) and compared to other Kanchanaburi samples (median f4 ratio = 7.0%, median $P=10^{-12}$). The distribution of this signal along the genome is noisy, but on several large genomic regions of several 100 kilobases can be identified, pointing to more recent introgression (fig. S7A). Thus, in three of the four wild *B. splendens* populations sampled, we observe evidence of introgression from ornamental betta. The relatively short introgressed segments suggest this introgression happened many generations ago.

6. Coalescent during bottleneck

The *fastsimcoal2* (*fsc2*) *instbot* model is able to robustly infer the timing of a simulated bottleneck with good accuracy for a variety of demographic models and parameter combinations (fig. S10E). The only exception to this is the case of the oldest bottleneck (2,000 generations ago) in the Relate-demography-based model, where the bottleneck time is underestimated. Although model comparisons based on relative likelihood values such as likelihood ratio tests or the Akaike information criterion are not appropriate given the composite nature of the computed likelihoods, we note that models with bottlenecks generally yielded higher absolute likelihoods compared to the null models except for combinations of recent bottlenecks of low intensity and small effective population size (T_{BOT}<=250, intensity<=0.2, Ne=10,000). Furthermore, inference on simulations without a bottleneck (intensity=0) yielded very small estimates of bottleneck

intensity (<0.0027). Taken together, these results suggest that the employed model can accurately detect and time recent bottlenecks for a variety of demographic parameters.

We ran 20 replicates each of the *fsc2 instbot* model described above on the ornamental betta and wild *B. splendens* samples from Kanchanaburi, which yielded average log-likelihoods of -3.41×10^6 and -4.42×10^6, respectively, both higher than the log-likelihoods in the respective null models with bottleneck intensity of zero (log-likelihood ratios 1.03×10^5 and 0.14×10^5, respectively). The parameter estimates of the *fsc2* runs are shown in Fig. 1G.

To assess whether a given bottleneck intensity inferred corresponds to an appreciable bottleneck, we checked whether given the sample size we expect to see an appreciable amount of lineages to coalesce during the bottleneck. The probability of no coalescent during a time interval t, given an effective size N, and a sample of n lineages is given by

$$e^{-t\binom{n}{2}/(2N)}$$

where $\binom{n}{2} = (n * (n-1)/2)$ is the number of pairs of lineages, and $\frac{1}{2N}$ is the coalescent rate for each pair. Hence for a bottleneck with intensity $I_{bot} := \frac{t_{bot}}{2N_{bot}}$, we can write the probability of no coalescent during the bottleneck as

$$e^{-I_{bot}\binom{n}{2}}.$$

Given these probabilities, the expected time to the next coalescent event given an effective population size N and a number of lineages n is given as

$$t_{coal}(n, N) = \frac{4N}{n(n-1)}$$

Hence, in order to get an estimate of the number of coalescent events expected to happen during a bottleneck we need solve the equations

$$\sum_{i=n}^{n'} t_{coal}(i, N_{before}) = T_{bot}$$

$$\sum_{i=n'}^{n''} t_{coal}(i, N_{bot}) = t_{bot}$$

where n are the number of sampled chromosomes (2*number of samples), T_{bot} is the time of the bottleneck (in generations before present), N_{before} is the assumed constant population size more recent than the start of the bottleneck, and t_{bot} and N_{bot} are the length and effective population size of the bottleneck, respectively. We note that the solution of these equations only depends on the ratio of t_{bot} and N_{bot} (which is the bottleneck intensity I_{bot}) and not on their absolute values. Solving these equations numerically yields the expected number of lineages coalescing during the bottleneck, n' - n''.

For the parameters inferred in the *fsc2 instbot* model we obtained that for ornamental Betta 55 out of 70 lineages (79%) are expected coalesce during the inferred bottleneck 680 generations ago, while for *B. splendens* Kanchanaburi 14 out of 42 lineages (33%) coalesce during the inferred bottleneck ~2240 generation ago. Conversely, we confirmed that no coalescences are expected to occur (n' - n'' = 0) in bottlenecks that were inferred from simulations that did not feature a bottleneck (simulated intensity = 0).

Supplementary methods

Species distribution

Approximate distribution ranges in Fig. 1 are based on the IUCN red list, except for *B. siamorientalis*, where an approximate range was drawn from ref. (*82*). The range for *B. splendens* was extended to the north based on records in ref. (*83*) and our own sampling locations.

Transposon Element library generation

We used the genomes of fAnaTes1.2 and fBetSpl5.3 to generate de novo TE consensus libraries using RepeatModeler2 using options LTR_retriever and LTR_harvest (*84*). This combined new library of de novo elements was used to annotate the genomes of fBetSpl5.3, Bspl.v1.2018.1, ASM365015v1, Betta_splendens_chrs.v1, and fAnaTes1.2, with RepeatMasker version open-4.0.9 (fig. S2).

Comparisons across reference assemblies

For synteny analyses, we used nucmer 4.0.0beta2 (*85*) on the following reference assemblies: *Oryzias latipes*: GCA_002234675.1; male wild *B. splendens*: GCF_900634795.3 (fBetSpl5.3); female ornamental *B. splendens* based on Oxford Nanopore (*dmrt1_XX*): GCA_013403625.1 (Bspl.v1.2018) (*12*); male ornamental *B. splendens* based on HiC (*dmrt1_XY*): GCA_003650155.1 (ASM365015v1) (*11*); ornamental *B. splendens* based on Illumina short reads: Betta_splendens_chrs.v1 (*13*); *Anabas testudineus*: GCA_900324465.2 (fAnaTes1.2) (*16*). We used mplotter (*86*) with '-filter' to generate dotplots of the nucmer output.

We searched for evidence of rearrangements involving *dmrt1* in the ornamental X. The long-molecule Oxford Nanopore reads from the ornamental female (*12*) did not contain any "chimeric" reads relative to the wild fBetSpl5.3 that would indicate an inversion.

Copy number calling and segmentation

We calculated and $\log_2$-normalized read counts in 1,000-bp intervals with a 500 bp slide across the nuclear genome. We called copy number states (CN0-CN8) implementing a Poisson logit-linked model (*87*). To assess more focal duplications such as gene-level duplications, we calculated the average read depth across each gene normalized to the length of the gene taken from the fBetSpl5.3 NCBI genome annotation.

Principal component analysis and population structure

We used plink v1.90p (*69*) with the options --double-id --allow-extra-chr to convert the filtered phased biallelic SNP vcf file to binary plink format. We used this file for PCA analyses in plink (option --pca) on (i) all samples except the outgroup individual Com0 and (ii) only *B. splendens* and ornamental betta samples in each case using a minor allele frequency cutoff of 1% (--maf 0.01).

After LD pruning using plink v1.90 (–indep-pairwise 50 10 0.2), we assessed population structure with Admixture v1.3.1 (*88*) using K=1-3 as number of clusters.

Estimation of the de novo mutation rate

To estimate the per generation mutation rate, we sequenced an ornamental trio and an ornamental quartet (two offspring to one set of parents) family at >30× coverage and used this in conjunction with the sequence data from the other 37 ornamental betta. We called and filtered variants as described in section **Alignment, variant detection, filtering, and phasing**. We also used the additional more stringent filtering criteria (similar to ref. (*21*)) to identify putative de novo mutations while reducing false positives. We removed sites with MQ0F>0.15, at which the average depth of coverage (DP) across samples was 1.65× below or above the genome-wide average (*89*), or that had a homozygous genotype for the non-reference allele. We excluded sites at which any ornamental (other than the offspring) were heterozygous, and sites with DP<10 in individuals of the trio or quartet. We also removed sites at which parents had one or more reads supporting the non-reference allele or at which the offspring had no read support of the alternate allele in either the forward or reverse strand.

Genotypes that were heterozygous in the offspring but homozygous for the reference allele in the parents were considered putative de novo mutations. The offspring of the trio had 5 de novo mutations, while the offspring of the quartet had 2 and 0 de novo mutations. We visualized each of these putative mutations in IGV for potential errors in alignment such as inclusion in reads near an indel, or with suspiciously high or low coverage compared to neighboring regions. We then calculated the accessible genome to use as the denominator in the mutation rate calculation by counting the number of bases remaining after the filters above, which yielded 311.9, 310.5, and 311.3 Mb for each trio. We multiplied the accessible genome by two because the mutation could have occurred in the paternal or maternal line and obtained a de novo mutation rate of 3.75×10^{-9} mutations per base pair per generation by dividing the 7 observed mutations by the total accessible genome. We estimated the 95% confidence interval using the exact method (*90*), which assumes the number of de novo mutations as a Poisson single value with a chi-square distribution (*21*).

Larvae RNA sequencing and allele-specific expression

We crossed a *dmrt1_XY* male to a *dmrt1_XX* female, genotyped by both RFLP and sequencing. We extracted genomic DNA and RNA of the fry at 4, 8, and 12 days post fertilization (dpf) using Zymo Microprep Kit. We generated RNAseq libraries for 3 *dmrt1_XY* and 3 *dmrt1_XX* 4-dpf larvae using NEBNext Ultra Directional II RNA Library Prep Kit for Illumina (E7760, NEB) and sequenced using NextSeq 500/550 (75 cycles). We aligned the reads using STAR2 (*91*) and counted reads with RSEM (*92*). Analysis of differential expression was done using DeSeq2 (*93*) implemented in R.

For targeted allele-specific expression analysis, we designed primers that spanned between exon 4 and exon 5 of *dmrt1* that included Tn5-ME adaptors, and flanked an X-Y variant on exon 5 (chromosome_9: 28864161) (table S3). We generated cDNA using LunaScript RT (E3010, NEB), and amplified the target region using Q5® High-Fidelity DNA Polymerase (M0491, NEB). We Illumina-indexed and sequenced the product using NextSeq 500/550 (75 cycles). We additionally performed sequence-based genotyping of heterozygous SNPs including this site across the 4, 8, and 12 dpf larvae (table S3). The number of alternate allele reads to total reads across the heterozygous sites fell within the variance of a normal binomial distribution for all sequence-based genotyping SNPs, suggesting that technical noise was minimal. We

performed a binomial test to assess potential allele-specific expression (ASE) at each dpf across larvae, implemented in R.

Carotenoids extraction

Frozen fish skin samples were ground with a mortar and a pestle precooled on dry ice. After weighing, the powder was homogenized in 250 µL phosphate-buffered saline. 250 µL of methanol was added and vortexed. After addition of 500 µL acetone, the suspension was vortexed and kept for 5 minutes on ice. 200 µL of diethyl ether and 400 µL hexane were added to extract carotenoids. Phase separation was achieved by centrifugation at 3,000x g for one minute. The upper organic layer was transferred to a new tube and the extraction repeated with 500 µL hexane. The combined organic phases were vacuum dried in a Speedvac (Eppendorf, Hauppauge, NY) and the debris dissolved in 200 µL hexane:ethyl acetate (70:30 v/v) for high-performance liquid chromatography (HPLC) analysis.

HPLC analysis

HPLC performed on a 1200 Agilent equipped with a diode array detector and normal-phase Zorbax silica column (4.6 mm ID x 150 mm with 5 µm packing; Agilent) as previously described (*94*). Chromatographic separation was achieved with isocratic flow of hexane:ethyl acetate (90:10 v/v) for β-carotene and retinoid separation and of hexane:ethyl acetate (70:30 v/v) for carotenoids, respectively. The flow rate was 1.4 mL/min. The system was scaled with known amounts of authentic standards.

Identification of echinenone

We compared the retention time and spectral characteristics of beta-beta-carotene-4,4'-dione (canthaxanthin) with an unknown peak (X) in the fish extracts. The absorption maximum X at 458 nm showed a bathochromic shift of ~9 nm when compared with the absorption maximum (467 nm) of canthaxanthin (fig. S19A). The addition of the second ketone group in canthaxanthin increases the carotenoid's polyene chain conjugation length, accompanied by a spectral shift towards the absorption of longer wavelength light. The absorption maximum agrees with the reported absorption maximum of echinenone in hexane as reported in the carotenoid database (http://carotenoiddb.jp). Further, peak X's retention time was shifted towards an earlier time point, being consistent with the loss of an oxo-group in one of the terminal β-ionone rings of canthaxanthin. Together with the characteristic shape of the absorption curve of peak X, this information strongly indicates that peak X is most likely echinenone.

Biochemical activity of β-carotene oxygenase I-like

We analyzed the enzymatic activity of the red-allele and blue-allele β-carotene oxygenase 1-like (BCO1L) protein by co-expressing the recombinant MBP-BCO1L fusion proteins in a β-carotene producing *E. coli* strain. This method has been previously used to characterize carotenoid cleavage enzymes from several species (*95*). The presence of β-carotene and various retinal-oxime diastereomers in the lipid extracts of these bacteria that express BCO1L display β-carotene 15-15'-dioxygenase activity (fig. S19C). We confirmed enzymatic activity with protein crude extracts after lysing the bacteria. The red-allele BCO1L displayed activity in these assays but the blue-allele BCO1L did not. We then enriched for the red-allele BCO1L by affinity chromatography using an established protocol for MBP-fused carotenoid cleavage dioxygenases

(*94*). The purified protein maintained its catalytic activity and converted β-carotene into all-*trans*-retinal similar to the control human BCO1 protein (fig. S19D).

We modelled the structures of red and blue allele BCO1L proteins using the recently determined *Candidatus Nitrosotalea devanaterra* (*Nd*CCD) structure as template (*96*) (fig. S19G). The Thr>Ile mutation is located at the surface of the protein and far away from the enzymes' active site (non-polar substrate tunnel). Therefore, the mutation most likely does not affect the substrate binding and/or catalytic activity of the enzyme. Notably, the recombinant enzymes expressed in *E.coli,* particularly the blue allele of BCOL1, showed relatively poor solubility when expressed in *E coli* and the majority of the protein was found in the insoluble protein fraction (inclusion bodies). We cannot predict whether this behavior in *E. coli* reflects in the natural cellular environment of the proteins. However, our analyses suggest that the polymorphism affects protein solubility and stability rather than catalytic activity of the enzymes.

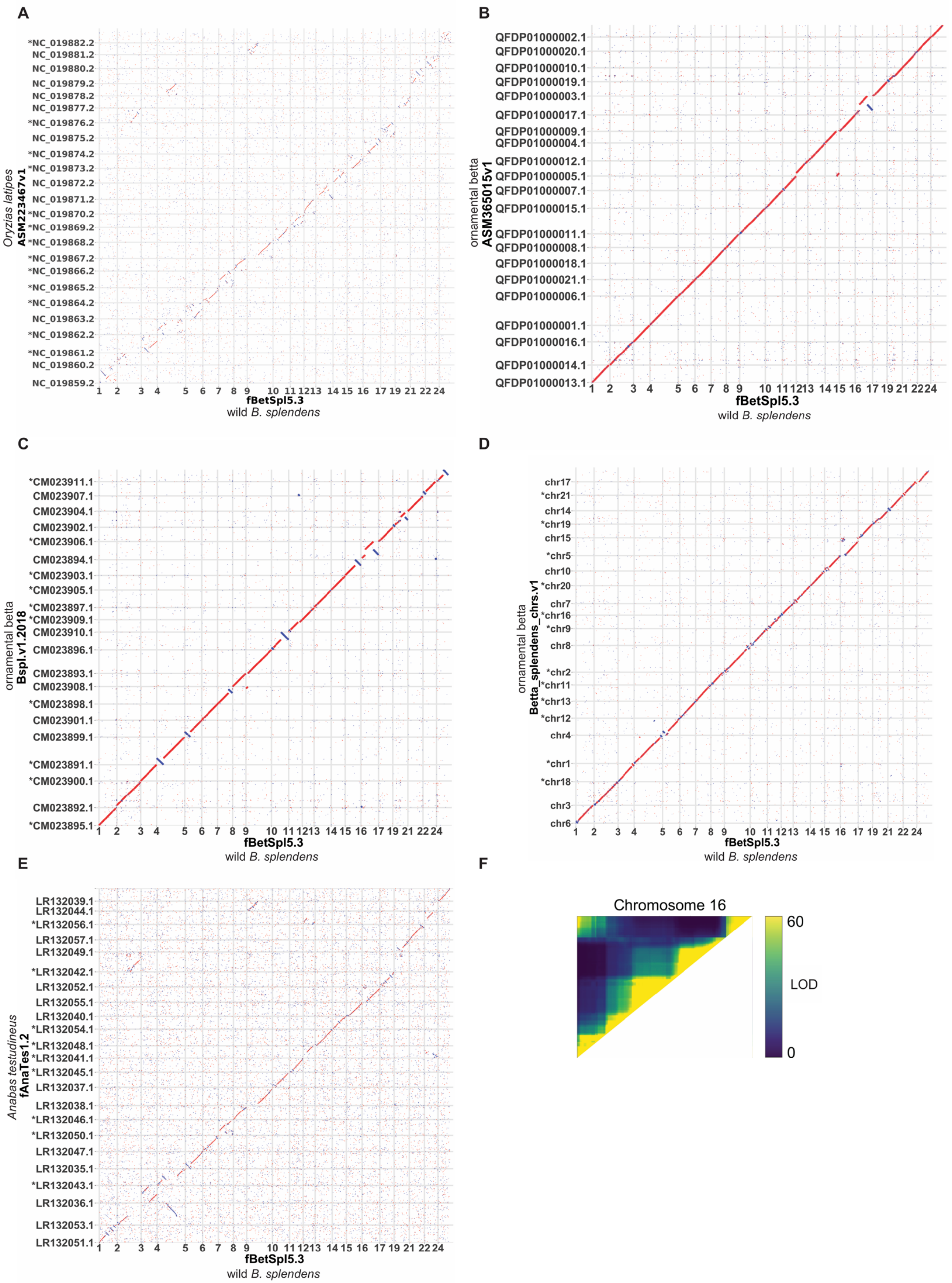
A
Oryzias latipes
ASM223467v1
*NC_019882.2
NC_019881.2
NC_019880.2
NC_019879.2
NC_019878.2
NC_019877.2
*NC_019876.2
NC_019875.2
*NC_019874.2
*NC_019873.2
NC_019872.2
NC_019871.2
*NC_019870.2
*NC_019869.2
*NC_019868.2
*NC_019867.2
*NC_019866.2
*NC_019865.2
*NC_019864.2
NC_019863.2
*NC_019862.2
*NC_019861.2
NC_019860.2
NC_019859.2
1 2 3 4 5 6 7 8 9 10 11 1213 14 15 16 17 19 21 22 24
fBetSpl5.3
wild B. splendens
B
ornamental betta
ASM365015v1
QFDP01000002.1
QFDP01000020.1
QFDP01000010.1
QFDP01000019.1
QFDP01000003.1
QFDP01000017.1
QFDP01000009.1
QFDP01000004.1
QFDP01000012.1
QFDP01000005.1
QFDP01000007.1
QFDP01000015.1
QFDP01000011.1
QFDP01000008.1
QFDP01000018.1
QFDP01000021.1
QFDP01000006.1
QFDP01000001.1
QFDP01000016.1
QFDP01000014.1
QFDP01000013.1
1 2 3 4 5 6 7 8 9 10 11 1213 14 15 16 17 19 21 22 24
fBetSpl5.3
wild B. splendens
C
ornamental betta
Bspl.v1.2018
*CM023911.1
CM023907.1
CM023904.1
CM023902.1
*CM023906.1
CM023894.1
*CM023903.1
*CM023905.1
*CM023897.1
*CM023909.1
CM023910.1
CM023896.1
CM023893.1
CM023908.1
*CM023898.1
CM023901.1
CM023899.1
*CM023891.1
*CM023900.1
CM023892.1
*CM023895.1
1 2 3 4 5 6 7 8 9 10 11 1213 14 15 16 17 19 21 22 24
fBetSpl5.3
wild B. splendens
D
ornamental betta
Betta_splendens_chrs.v1
chr17
*chr21
chr14
*chr19
chr15
*chr5
chr10
*chr20
chr7
*chr16
*chr9
chr8
*chr2
*chr11
*chr13
*chr12
chr4
*chr1
*chr18
chr3
chr6
1 2 3 4 5 6 7 8 9 10 11 1213 14 15 16 17 19 21 22 24
fBetSpl5.3
wild B. splendens
E
Anabas testudineus
fAnaTes1.2
LR132039.1
LR132044.1
*LR132056.1
LR132057.1
LR132049.1
*LR132042.1
LR132052.1
LR132055.1
LR132040.1
*LR132054.1
*LR132048.1
*LR132041.1
*LR132045.1
LR132037.1
LR132038.1
*LR132046.1
*LR132050.1
LR132047.1
LR132035.1
*LR132043.1
LR132036.1
LR132053.1
LR132051.1
1 2 3 4 5 6 7 8 9 10 11 1213 14 15 16 17 19 21 22 24
fBetSpl5.3
wild B. splendens
F
Chromosome 16
60
LOD
0

Fig. S1. Cross reference genome comparisons

A, Synteny plot of wild *B. splendens* fBetSpl5.3 vs. medaka ASM223467v1 reference genome (*81*). **B,** Synteny plot of fBetSpl5.3 vs. ornamental betta ASM365015v1 reference genome (*11*). **C,** Synteny plot of fBetSpl5.3 vs. ornamental betta Bspl.v1.2018 reference genome (*12*). **D,** Synteny plot of fBetSpl5.3 vs. ornamental betta Betta_splendens_chrs.v1 reference genome (*13*). **E,** Synteny plot of fBetSpl5.3 vs. *Anabas testudineus* fAnaTes1.2 reference genome (*16*). **A-E,** Unplaced contigs were removed in the synteny plots. Asterisks indicate chromosomes with an opposite orientation from fBetSpl5.3. **F,** LOD scores of genetic map for chromosome 16 of an F2 intercross mapped against fBetSpl5.3 reference genome shows the left part of the chromosome is linked to the right part, consistent with an inversion in ornamental relative to the fBetSpl5.3 wild *B. splendens* reference genome.

A

Percent of Genome Masked

	Reference genomes				
	**fBetSpl5.3**	**Bspl.v1.2018**	**ASM365015v1**	**Betta_splendens chrs.v1**	**fAnaTes1.2**
Long Terminal Repeat (LTR)	4.82	4.55	2.79	2.41	1.039
Short Interspersed Nuclear Element (SINE)	0.07	0.07	0.07	0.07	0.147
Long Interspersed Nuclear Element (LINE)	4.87	4.86	3.08	2.55	3.636
Rolling Circular transposon (RC)	0.24	0.15	0.05	0.04	0.045
DNA	2.95	2.83	2.3	2.13	4.666
Unknown	6.46	7.39	4.22	3.88	4.824
Other	3.15	3.17	2.27	1.76	2.26
**TOTAL**	22.57	23.03	14.79	12.83	16.62

B

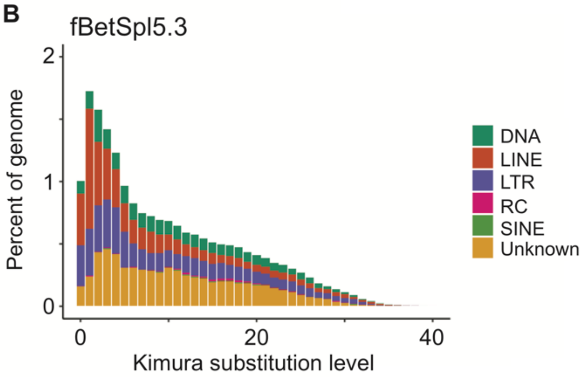

C

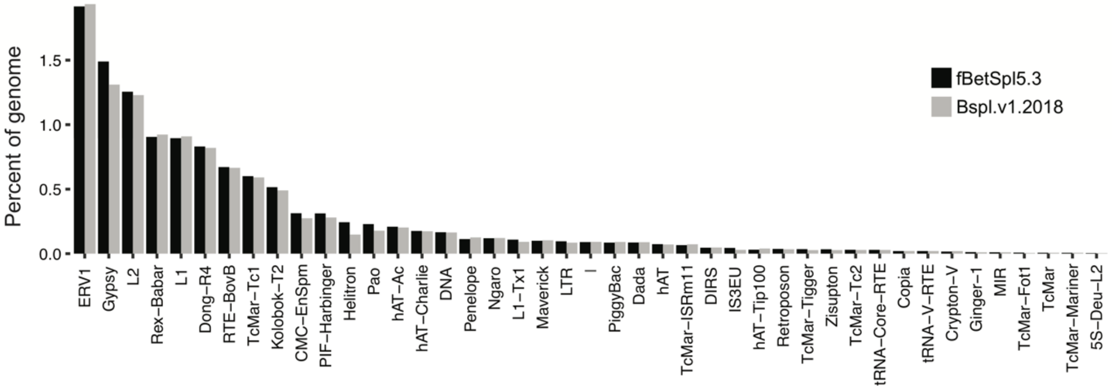

Fig. S2. Reference genomes masking

A, Genome masked across wild *B. splendens* and ornamental betta reference genomes: fBetSpl5.3 (wild *B. splendens*; this paper); Bspl.v1.2018 (*11*), Oxford nanopore read assembly polished with ASM365015v1 (*12*), and short read sequencing (*13*). **B,** Kimura-based sequence divergence across transposable element families in fBetSpl5.3. **C,** Annotation of transposons across fBetSpl5.3 and Bspl.v1.2018.

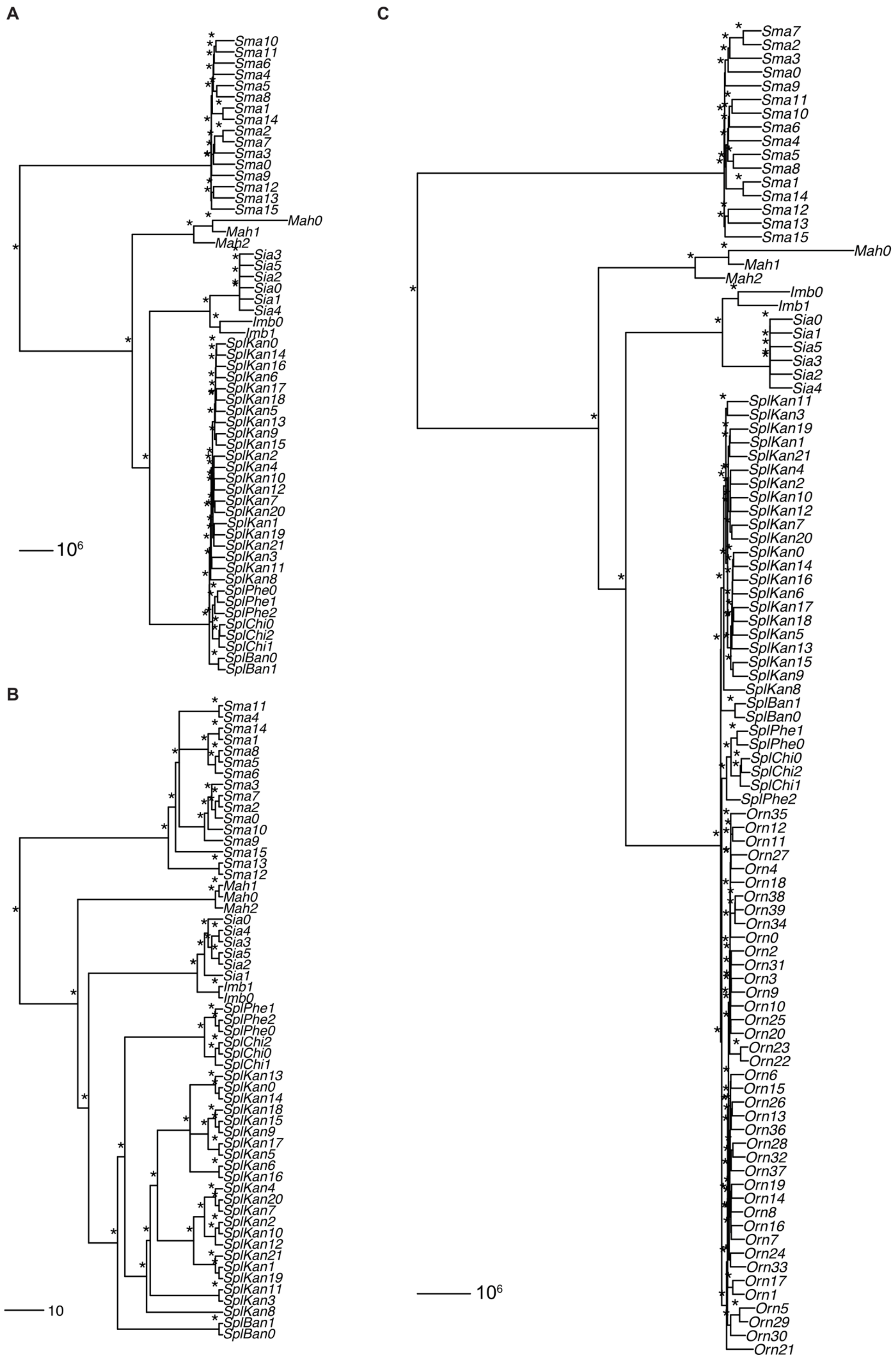
A
Sma10
Sma11
Sma6
Sma4
Sma5
Sma8
Sma1
Sma14
Sma2
Sma7
Sma3
Sma0
Sma9
Sma12
Sma13
Sma15
Mah0
Mah1
Mah2
Sia3
Sia5
Sia2
Sia0
Sia1
Sia4
Imb0
Imb1
SplKan0
SplKan14
SplKan16
SplKan6
SplKan17
SplKan18
SplKan5
SplKan13
SplKan9
SplKan15
SplKan2
SplKan4
SplKan10
SplKan12
SplKan7
SplKan20
SplKan1
SplKan19
SplKan21
SplKan3
SplKan11
SplKan8
SplPhe0
SplPhe1
SplPhe2
SplChi0
SplChi2
SplChi1
SplBan0
SplBan1
10^6
B
Sma11
Sma4
Sma14
Sma1
Sma8
Sma5
Sma6
Sma3
Sma7
Sma2
Sma0
Sma10
Sma9
Sma15
Sma13
Sma12
Mah1
Mah0
Mah2
Sia0
Sia4
Sia3
Sia5
Sia2
Sia1
Imb1
Imb0
SplPhe1
SplPhe2
SplPhe0
SplChi2
SplChi0
SplChi1
SplKan13
SplKan0
SplKan14
SplKan18
SplKan15
SplKan9
SplKan17
SplKan5
SplKan6
SplKan16
SplKan4
SplKan20
SplKan7
SplKan2
SplKan10
SplKan12
SplKan21
SplKan1
SplKan19
SplKan11
SplKan3
SplKan8
SplBan1
SplBan0
10
C
Sma7
Sma2
Sma3
Sma0
Sma9
Sma11
Sma10
Sma6
Sma4
Sma5
Sma8
Sma1
Sma14
Sma12
Sma13
Sma15
Mah0
Mah1
Mah2
Imb0
Imb1
Sia0
Sia1
Sia5
Sia3
Sia2
Sia4
SplKan11
SplKan3
SplKan19
SplKan1
SplKan21
SplKan4
SplKan2
SplKan10
SplKan12
SplKan7
SplKan20
SplKan0
SplKan14
SplKan16
SplKan6
SplKan17
SplKan18
SplKan5
SplKan13
SplKan15
SplKan9
SplKan8
SplBan1
SplBan0
SplPhe1
SplPhe0
SplChi0
SplChi2
SplChi1
SplPhe2
Orn35
Orn12
Orn11
Orn27
Orn4
Orn18
Orn38
Orn39
Orn34
Orn0
Orn2
Orn31
Orn3
Orn9
Orn10
Orn25
Orn20
Orn23
Orn22
Orn6
Orn15
Orn26
Orn13
Orn36
Orn28
Orn32
Orn37
Orn19
Orn14
Orn8
Orn16
Orn7
Orn24
Orn33
Orn17
Orn1
Orn5
Orn29
Orn30
Orn21
10^6

Fig. S3. Phylogeny of *Betta splendens* species-complex.

A, Neighbor-joining phylogeny based on whole-genome pairwise genetic differences of biallelic SNPs across species of the *Betta splendens* complex. Asterisks denote block bootstrap support values greater than 990/1000 (99%). Block bootstrap is based on pairwise differences computed across 4,361 windows of 100 kb. The tree was rooted with the outgroup *B. compuncta*. **B,** Maximum-likelihood phylogeny based on variant SNPs across the genome excluding ornamental betta. **C,** Neighbor-joining phylogeny based on bi-allelic SNPs across species of the *Betta splendens* complex including ornamental betta. Scale indicates differences in bp (**A,C**) and percent (**B**).

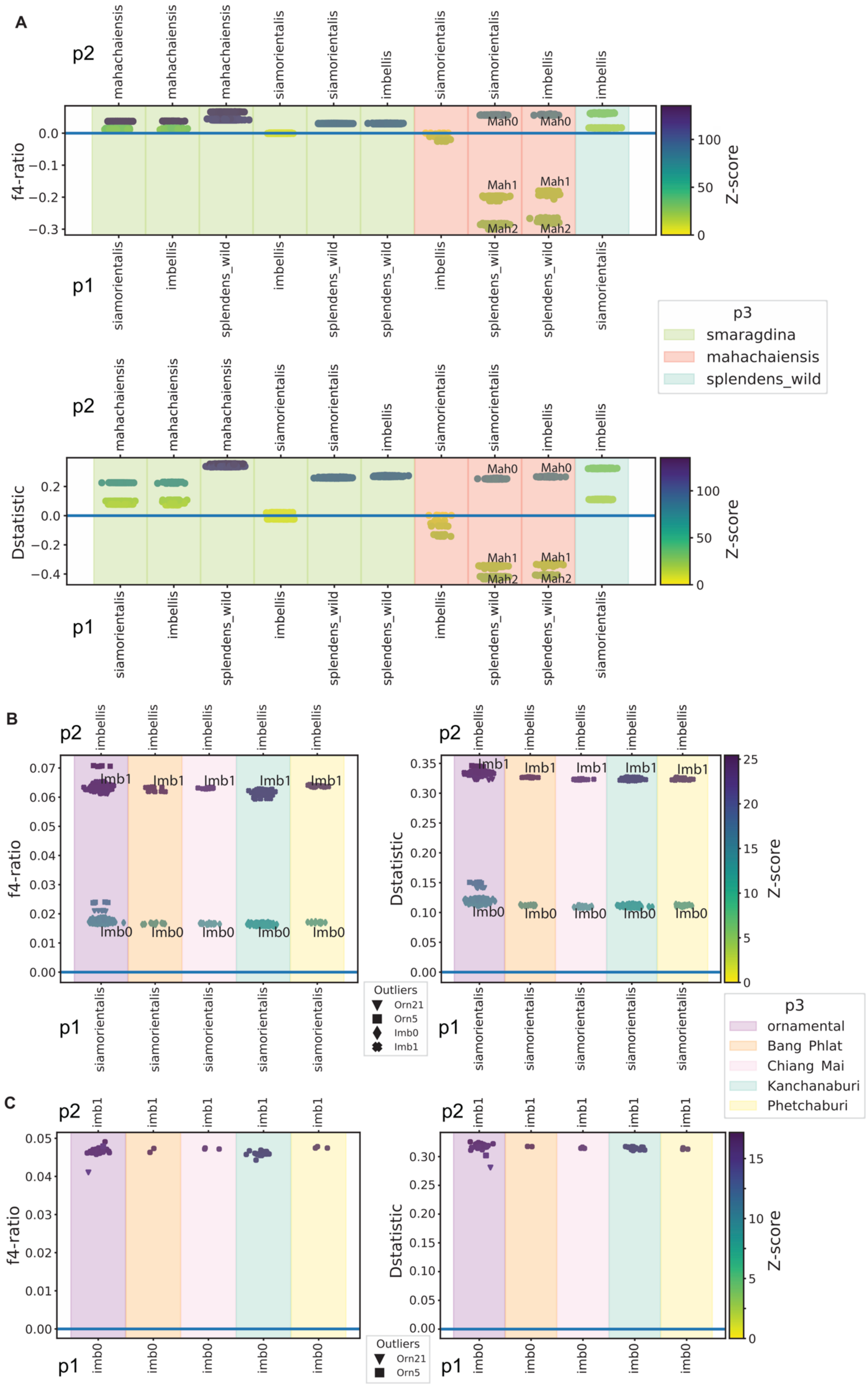
A
B
C
p1
p2
p3
f4-ratio
Dstatistic
Z-score
smaragdina
mahachaiensis
splendens_wild
ornamental
Bang Phlat
Chiang Mai
Kanchanaburi
Phetchaburi
Outliers
Orn21
Orn5
Imb0
Imb1
Mah0
Mah1
Mah2
siamorientalis
imbellis
imb0
imb1

Fig. S4. Excess allele sharing across *B. splendens* species complex and ornamental betta.

A, Individual f4 admixture ratio tests and Patterson's *D* tests between species of the *Betta splendens* species complex. p1, p2, p3 individuals are taken from different wild *Betta* species so that ((p1, p2), p3) is consistent with the genome-wide phylogeny in fig. S3a. All f4 admixture ratio and *D* tests follow the order, f4 or *D* (p1, p2, p3, *B. compuncta*) (**A-C**). Color of dots indicate block-jackknife significance as Z-score; panel background colors indicate p3 (**A-C**). **B,** Excess allele sharing among *B. siamorientalis* and *imbellis* with each of the wild *B. splendens* populations and with ornamental betta. f4 admixture ratio and *D* tests with the order: p1=*B. siamorientalis,* p2=*B. imbellis*, and p3=*B. splendens* population (Bang Phlat, Chiang Mai, Kanchanaburi, Phetchaburi) or ornamental betta. **C,** Excess allele sharing among the two *B. imbellis* samples with wild *B. splendens* and ornamental betta. f4 ratio and *D* tests with the order: p1=Imb0 (*B. imbellis* individual), p2=Imb1 (*B. imbellis* individual), and p3=*B. splendens* population (Bang Phlat, Chiang Mai, Kanchanaburi, Phetchaburi) or ornamental betta.

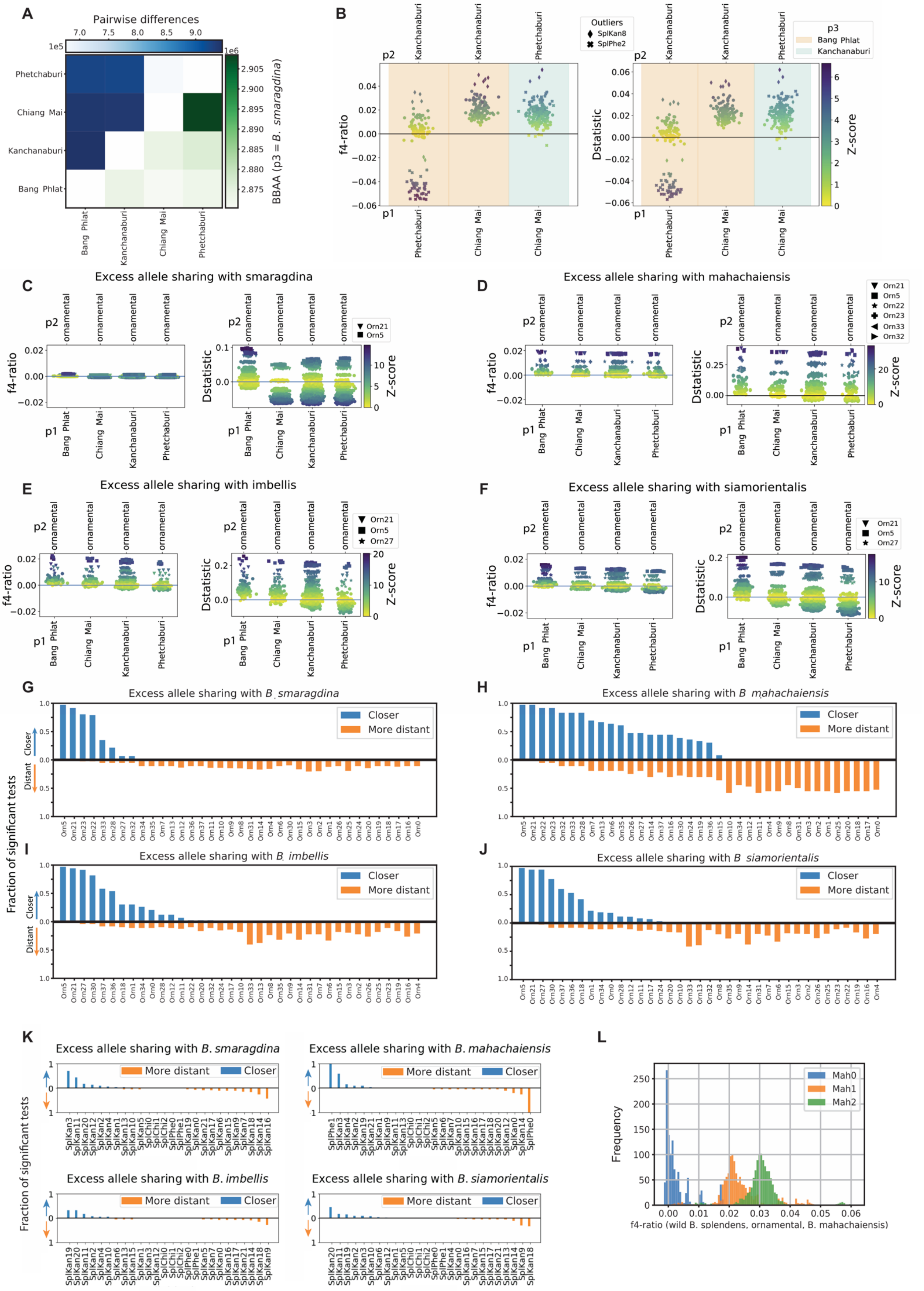

A
Pairwise differences
7.0
7.5
8.0
8.5
9.0
1e5
1e6
Phetchaburi
Chiang Mai
Kanchanaburi
Bang Phlat
2.905
2.900
2.895
2.890
2.885
2.880
2.875
BBAA (p3 = B. smaragdina)
B
Outliers
SplKan8
SplPhe2
p3
Bang Phlat
Kanchanaburi
p2
p1
f4-ratio
Dstatistic
Z-score
C
Excess allele sharing with smaragdina
D
Excess allele sharing with mahachaiensis
E
Excess allele sharing with imbellis
F
Excess allele sharing with siamorientalis
ornamental
Orn21
Orn5
Orn22
Orn23
Orn33
Orn32
Orn27
G
Excess allele sharing with B. smaragdina
H
Excess allele sharing with B. mahachaiensis
I
Excess allele sharing with B. imbellis
J
Excess allele sharing with B. siamorientalis
Closer
More distant
Distant
Fraction of significant tests
K
Excess allele sharing with B. smaragdina
Excess allele sharing with B. mahachaiensis
Excess allele sharing with B. imbellis
Excess allele sharing with B. siamorientalis
L
Mah0
Mah1
Mah2
Frequency
f4-ratio (wild B. splendens, ornamental, B. mahachaiensis)

Fig. S5. Excess allele sharing between *B. splendens* and ornamental betta.

A, Pairwise genetic differences (above diagonal) and BBAA counts (below diagonal) between wild *B. splendens* populations. BBAA counts are based on Dsuite output where p3=*B. smaragdina*. **B,** f4-ratio and *D* tests between individuals of wild *B. splendens* populations. Color of dots indicate block-jackknife significance as z-score of each test. Panel background colors indicate p3; ornamental individuals that are outliers, with high-average f4 or *D* are shown in different shapes (**C-F).** Individual f4-ratio and *D* tests between wild *B. splendens* populations and ornamentals relative to *smaragdina* (**C**), *mahachaiensis* (**D**), *imbellis* (**E**), and *siamorientalis* (**F**). **G-J,** Proportion of ABBA-BABA tests *D*(ornamentals except focal, focal ornamental; non-*splendens* species, *compuncta*), where a focal ornamental individual is significantly closer to a non-splendens species compared to other ornamental individuals (D>0 significant, Closer, blue) and fraction where a focal ornamental individual is significantly more distant from a non-*splendens* species compared to other ornamental individuals (D<0 significant, More distant, orange). Non-*splendens* species are *smaragdina* (**G**), *mahachaiensis* (**H**), *imbellis* (**I**), and *siamorientalis* (**J**). **K,** Proportion of ABBA-BABA tests *D*(wild population same as focal, wild focal; non-*splendens* species, *compuncta*) that are significantly positive (Closer, blue) and negative (More distant, orange). Non-*splendens* species are *smaragdina*, *mahachaiensis*, *imbellis*, and *siamorientalis* (ordered top left to bottom right). **G-K,** A z-score of 5.49 is used as significance cutoff corresponding to a Bonferroni-corrected *P*=0.01. **l,** Excess allele sharing between *B. mahachaiensis* samples (Mah0, Mah1, Mah2) and ornamental betta relative to wild *B. splendens*. Individual f4-ratio tests with p1=ornamental betta, p2=wild *B. splendens,* and p3=Mah0 (blue), Mah1 (orange), or Mah2 (green). Note that for (**D**), (**H**), and (**I**) only Mah0 was used in the comparisons but not Mah1 and Mah2.

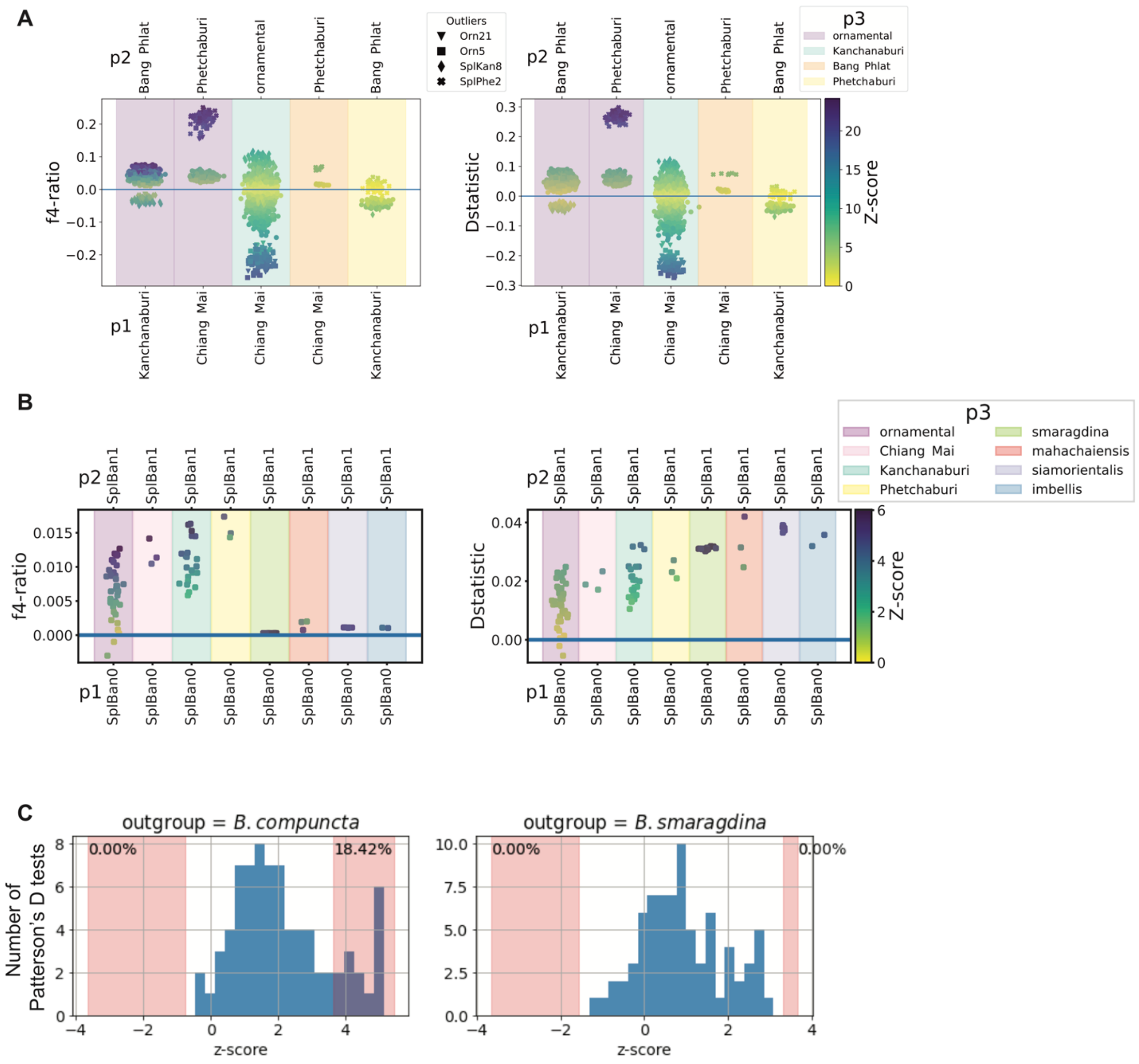
A
Outliers
Orn21
Orn5
SplKan8
SplPhe2
p2
Bang Phlat
Phetchaburi
ornamental
Phetchaburi
Bang Phlat
f4-ratio
Dstatistic
p1
Kanchanaburi
Chiang Mai
Chiang Mai
Chiang Mai
Kanchanaburi
p3
ornamental
Kanchanaburi
Bang Phlat
Phetchaburi
Z-score
B
p3
ornamental
Chiang Mai
Kanchanaburi
Phetchaburi
smaragdina
mahachaiensis
siamorientalis
imbellis
p2
SplBan1
f4-ratio
Dstatistic
p1
SplBan0
Z-score
C
outgroup = B. compuncta
outgroup = B. smaragdina
Number of Patterson's D tests
0.00%
18.42%
0.00%
0.00%
z-score

Fig. S6. Excess allele sharing between *B. splendens* populations and ornamental betta.

A, Excess allele sharing between wild *B. splendens* populations and ornamental betta. Individual f4 (left) or *D* (right) (p1, p2, p3, *B. compuncta*) test, where p1, p2, p3 individuals are taken from different *B. splendens* populations and ornamental betta so that ((p1, p2), p3) is consistent with the genome-wide phylogeny in fig. S3A. Outlier individuals are represented by different markers. Color of markers indicates block-jackknife significance as z-score; panel background colors indicate p3 (**A,B**). **B**, Excess allele sharing of SplBan1 relative to SplBan0 with individuals of ornamental betta, other wild *B. splendens* populations and non-*splendens* species. **C**, Distribution of z-scores of individual Patterson's D tests of the form D(SplBan0, SplBan1; p3 , outgroup), where p3 represents each individual sample except SplBan0, SplBan1 and samples from *B. smaragdina*. The outgroup is *B. compuncta* in the left panel and *B. smaragdina* in the right panel. Pink shaded areas correspond to z-scores of Bonferroni-corrected *P*-values < 0.01. Percentages correspond to the percentage of comparisons in which SplBan0 (left) and SplBan1(right) individuals are significantly closer to other individuals.

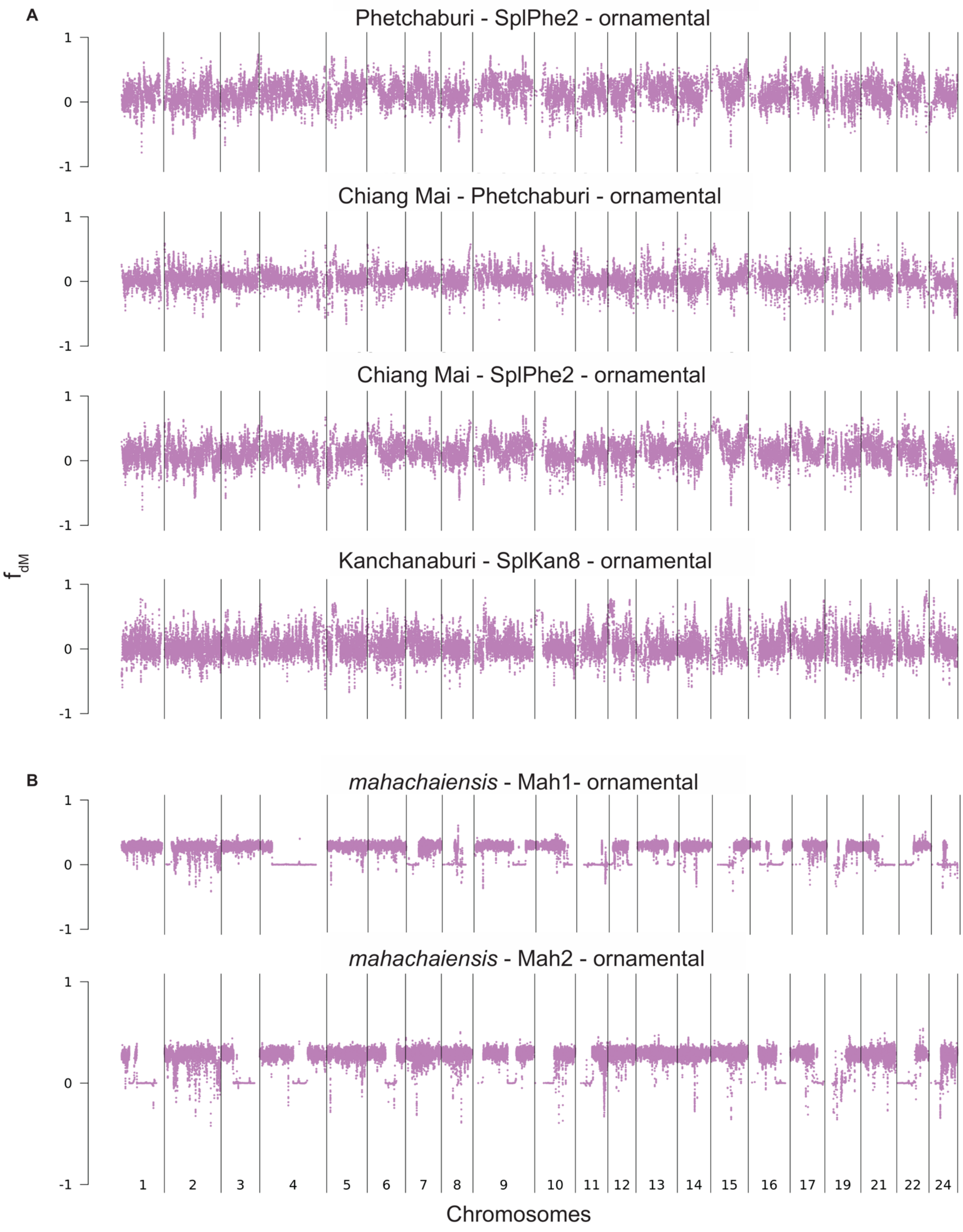
A
Phetchaburi - SplPhe2 - ornamental
Chiang Mai - Phetchaburi - ornamental
Chiang Mai - SplPhe2 - ornamental
Kanchanaburi - SplKan8 - ornamental
B
mahachaiensis - Mah1- ornamental
mahachaiensis - Mah2 - ornamental
fdM
1
0
-1
1 2 3 4 5 6 7 8 9 10 11 12 13 14 15 16 17 19 21 22 24
Chromosomes

Fig. S7. Genome-wide f_{dM} for wild *B. splendens* and non-*splendens* to ornamental betta. Genome-wide f_{dM} plots with ordering specified on the title in the format (p1 - p2 - p3). The outgroup p4 is *B. compuncta*. Each point represents 100 SNPs. Positive f_{dM} values measure excess allele sharing between p2 and p3 (*20*), **A,** wild *B. splendens* individuals (p2) with ornamental introgression. **B,** *B. mahachaiensis* samples with ornamental introgression.

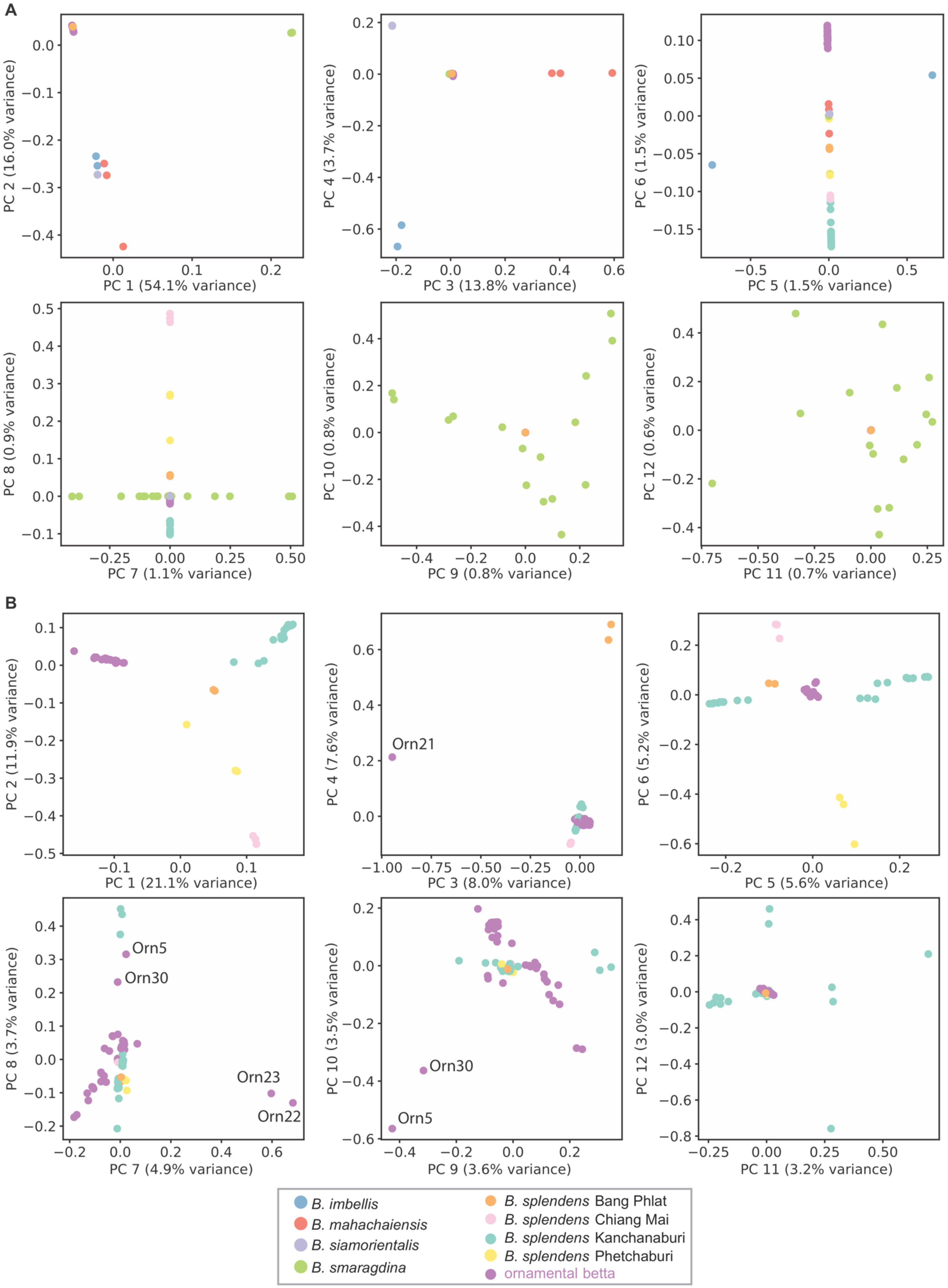

A
B
PC 1 (54.1% variance)
PC 2 (16.0% variance)
PC 3 (13.8% variance)
PC 4 (3.7% variance)
PC 5 (1.5% variance)
PC 6 (1.5% variance)
PC 7 (1.1% variance)
PC 8 (0.9% variance)
PC 9 (0.8% variance)
PC 10 (0.8% variance)
PC 11 (0.7% variance)
PC 12 (0.6% variance)
PC 1 (21.1% variance)
PC 2 (11.9% variance)
PC 3 (8.0% variance)
PC 4 (7.6% variance)
Orn21
PC 5 (5.6% variance)
PC 6 (5.2% variance)
PC 7 (4.9% variance)
PC 8 (3.7% variance)
Orn5
Orn30
Orn23
Orn22
PC 9 (3.6% variance)
PC 10 (3.5% variance)
Orn30
Orn5
PC 11 (3.2% variance)
PC 12 (3.0% variance)
B. imbellis
B. mahachaiensis
B. siamorientalis
B. smaragdina
B. splendens Bang Phlat
B. splendens Chiang Mai
B. splendens Kanchanaburi
B. splendens Phetchaburi
ornamental betta

Fig. S8. Population structure across species of *B. splendens* species complex and populations of *B. splendens*.

A, Principal component analysis of genetic variation across all samples of the *B. splendens* species complex including ornamental betta. **B**, Principal component analysis of *B. splendens* samples including ornamental betta.

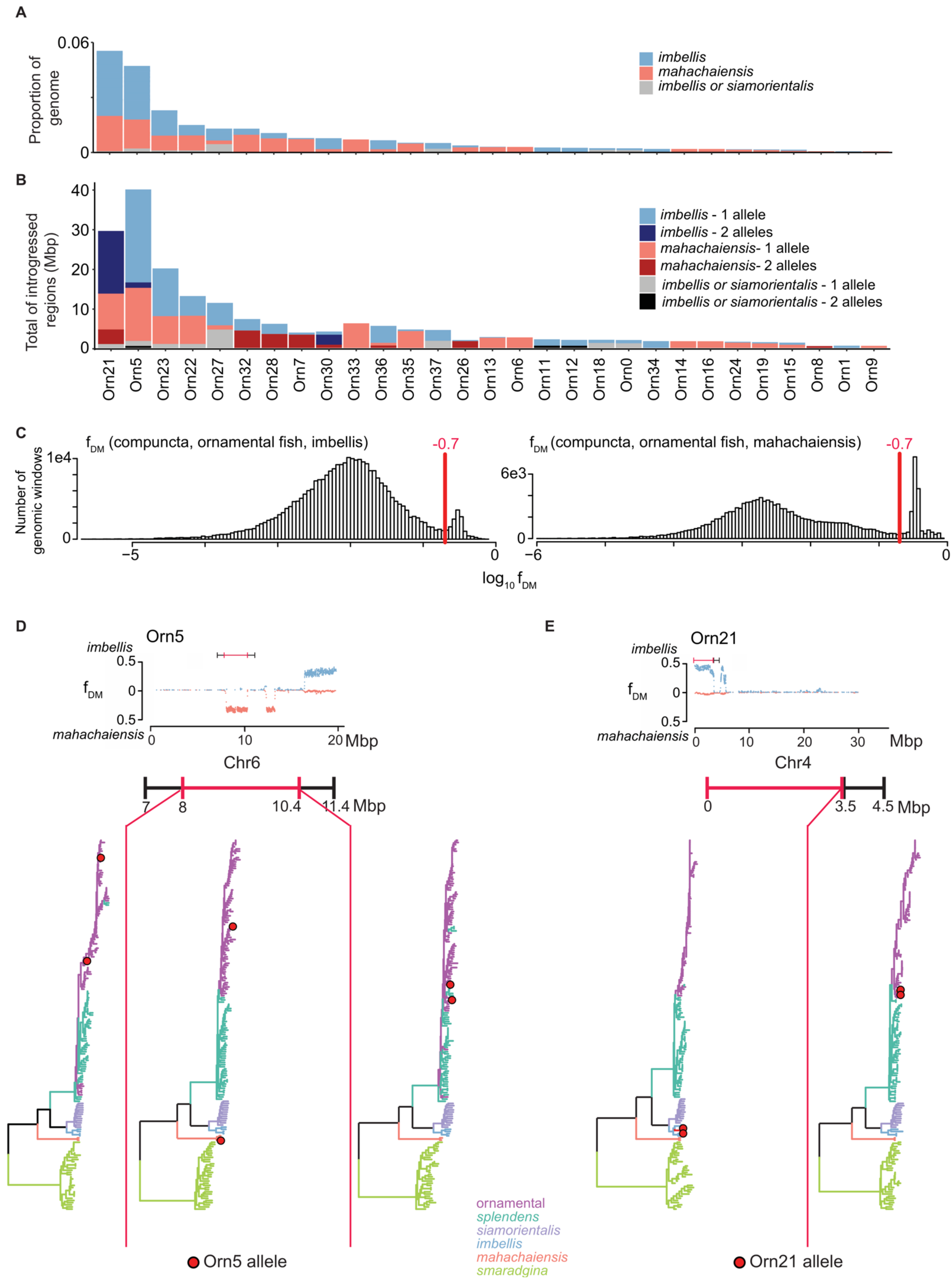

A
Proportion of genome
0.06
0
imbellis
mahachaiensis
imbellis or siamorientalis
B
Total of introgressed regions (Mbp)
40
30
20
10
0
imbellis - 1 allele
imbellis - 2 alleles
mahachaiensis- 1 allele
mahachaiensis- 2 alleles
imbellis or siamorientalis - 1 allele
imbellis or siamorientalis - 2 alleles
Orn21 Orn5 Orn23 Orn22 Orn27 Orn32 Orn28 Orn7 Orn30 Orn33 Orn36 Orn35 Orn37 Orn26 Orn13 Orn6 Orn11 Orn12 Orn18 Orn0 Orn34 Orn14 Orn16 Orn24 Orn19 Orn15 Orn8 Orn1 Orn9
C
Number of genomic windows
f DM (compuncta, ornamental fish, imbellis)
-0.7
1e4
0
−5
0
f DM (compuncta, ornamental fish, mahachaiensis)
-0.7
6e3
0
−6
0
log10 f DM
D
Orn5
imbellis
0.5
f DM
0
0.5
mahachaiensis
0
10
20
Mbp
Chr6
7
8
10.4
11.4 Mbp
E
Orn21
imbellis
0.5
f DM
0
0.5
mahachaiensis
0
10
20
30
Mbp
Chr4
0
3.5
4.5 Mbp
ornamental
splendens
siamorientalis
imbellis
mahachaiensis
smaradgina
Orn5 allele
Orn21 allele

Fig. S9. Gene flow from non-*splendens* into ornamental betta.

A, Proportion of the diploid genome with introgression per ornamental betta, color coded by ancestry. Grey shading indicates ancestry which could not be assigned exclusively to either *B. siamorientalis* or *B. imbellis*. **B**, Sum of length of introgressed regions. Lighter shading indicates regions where introgression occurs on a single allele; darker, both alleles. Ordering of ornamental betta is based on decreasing proportion of introgressed genome, and is the same for both panels. **C**, f_{dM} distributions of 100 SNP genomic windows across all ornamental fish with respect to *compuncta* (p1) and *imbellis* (p3) or *mahachaiensis* (p3). **D,E**, Chromosomal f_{dM} plots of Orn5 and Orn21 with local regional trees that span a region of high f_{dM} to *mahachaiensis* or *imbellis,* and 100-kb regions directly before and after. Tips corresponding to the alleles of the focal ornamental individuals (Orn5 or Orn21) are marked by red circles. Branches and tips are color coded by species.

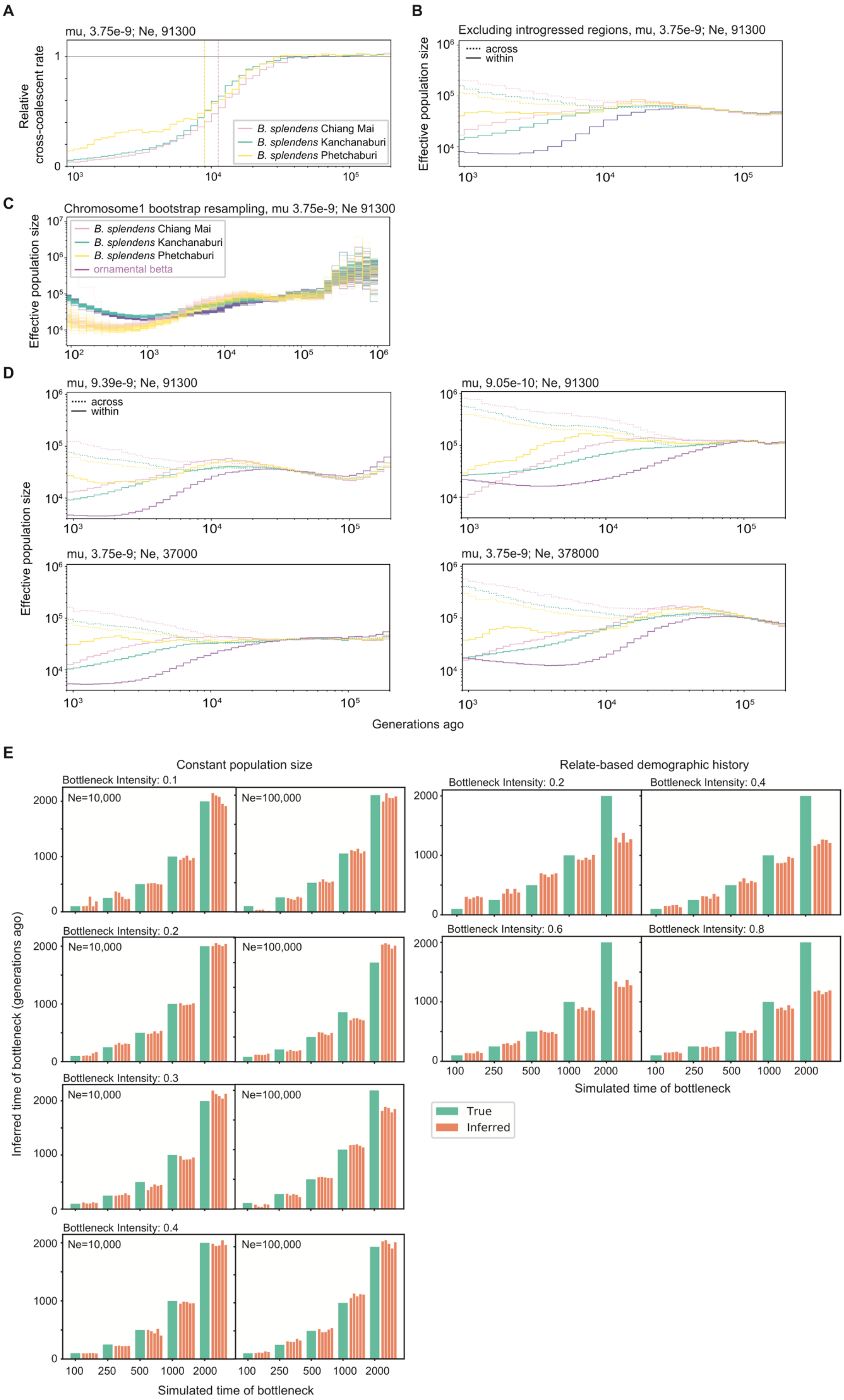
A
mu, 3.75e-9; Ne, 91300
Relative cross-coalescent rate
B. splendens Chiang Mai
B. splendens Kanchanaburi
B. splendens Phetchaburi
B
Excluding introgressed regions, mu, 3.75e-9; Ne, 91300
across
within
Effective population size
C
Chromosome1 bootstrap resampling, mu 3.75e-9; Ne 91300
B. splendens Chiang Mai
B. splendens Kanchanaburi
B. splendens Phetchaburi
ornamental betta
Effective population size
D
mu, 9.39e-9; Ne, 91300
across
within
mu, 9.05e-10; Ne, 91300
mu, 3.75e-9; Ne, 37000
mu, 3.75e-9; Ne, 378000
Effective population size
Generations ago
E
Constant population size
Relate-based demographic history
Bottleneck Intensity: 0.1
Bottleneck Intensity: 0.2
Bottleneck Intensity: 0.3
Bottleneck Intensity: 0.4
Bottleneck Intensity: 0.6
Bottleneck Intensity: 0.8
Ne=10,000
Ne=100,000
Inferred time of bottleneck (generations ago)
Simulated time of bottleneck
True
Inferred

Fig. S10. Demographic inferences.

A, Relative cross-coalescent rates (RCCR) between wild *B. splendens* populations and ornamental betta, using Relate. Vertical lines denote the time at the 50% RCCR. **B,** Relate demographies of wild *B. splendens* populations and ornamental betta after excluding genomic regions with evidence of introgression from non-*B. splendens* species. Within population demographies are denoted by solid lines. Across-population effective population sizes between wild populations and ornamental betta are denoted by dotted lines. **C,** Relate demographies of 100 runs of bootstrap resampling of 5-kb blocks across Chromosome 1. Lines denote independent runs for a population. **D**, Demographic history inference in ornamental betta and wild *B. splendens* populations using Relate under different parameters. Upper left panel: demography using the upper confidence interval for the mutation rate (9.39×10^{-9} mutations per bp per generation); upper right panel: lower confidence interval (9.05×10^{-10}). Lower left panels: demography using the starting effective population size (37,000) based on upper confidence interval for the mutation rate (9.39×10^{-9} mutations per bp per generation); lower right panel: demography using the starting effective population size (378,000) based on lower confidence interval (9.05×10^{-10}). **E,** *fastsimcoal2* inferences based on demographic histories simulated in msprime with (left) constant effective population size and (right) the Relate-inferred demography. Bottlenecks of various intensities and timings were simulated on top of these demographies. See Methods for more details.

A

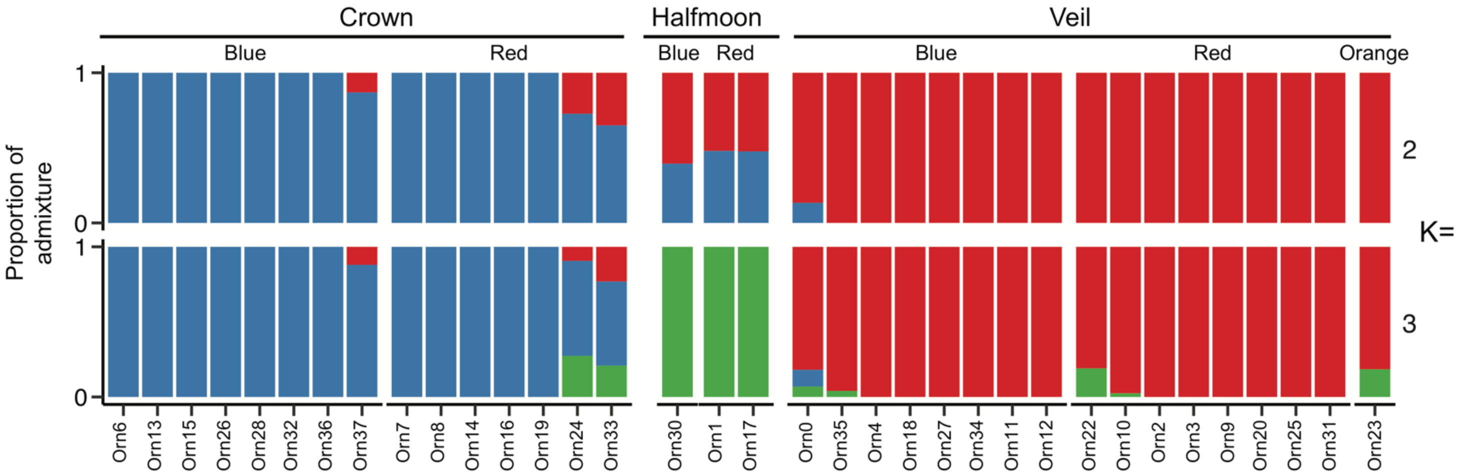

B

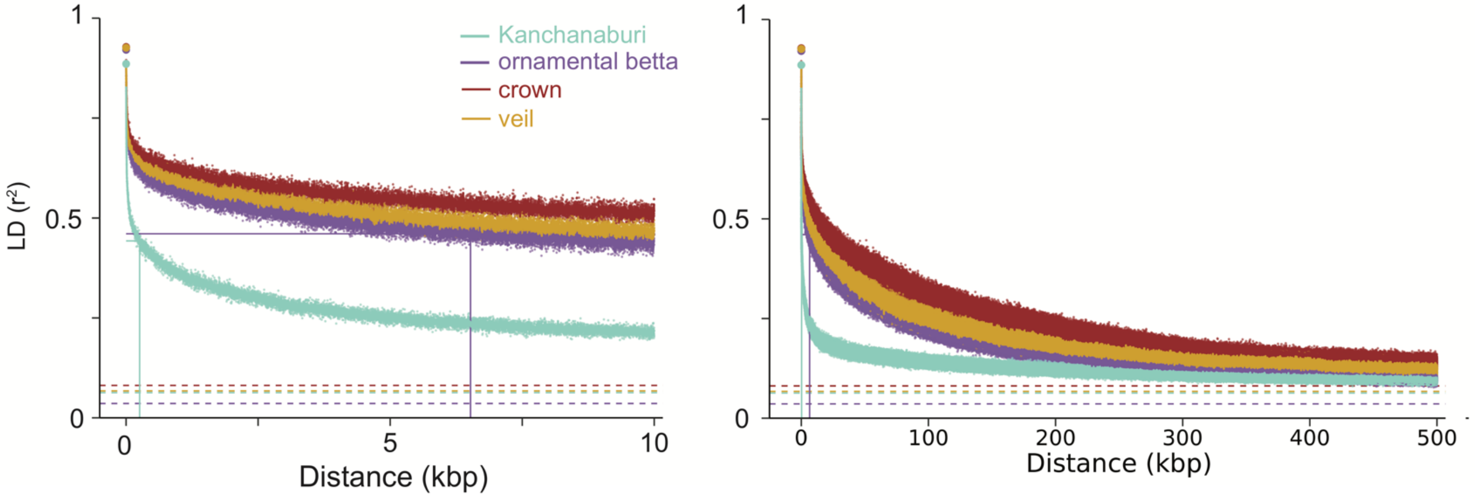

C

		nucleotide diversity	
		all sites	excluding introgressed sites
Ornamental	crown	0.00105, CI: ±1.6 x 10^{-10}	0.000907, CI: ±9.6 x 10^{-11}
	veil	0.00107, CI: ±1.0 x 10^{-10}	0.00101, CI: ±7.5 x 10^{-11}
	all	0.00113, CI: ±1.2 x 10^{-10}	0.00102, CI: ±8.2 x 10^{-11}
Kanchanaburi	all	0.00137, CI: ±6.9 x10^{-11}	0.00138, CI: ±8.9 x 10^{-11}

Fig. S11. Population structure, linkage disequilibrium decay and nucleotide diversity.

A, Admixture plot of ornamentals across K (2,3) clusters. **B**, Linkage disequilibrium decay of ornamental and wild *B. splendens* from Kanchanaburi. Half-max for ornamentals: 6.1 kb and for wild: 256 bp. Horizontal dashed lines denote interchromosomal r^2 (ornamental: 0.038; crown: 0.081; veil: 0.067; wild: 0.063). **C**, Nucleotide diversity in ornamental betta and wild *B. splendens* from Kanchanaburi. Diversity in ornamental betta calculated for all 37 individuals combined and split into the 16 crowntail and 18 veiltail fish and also calculated after sites with evidence of introgression are excluded. Confidence Intervals (CI, calculated based on jackknife resampling of chromosomes).

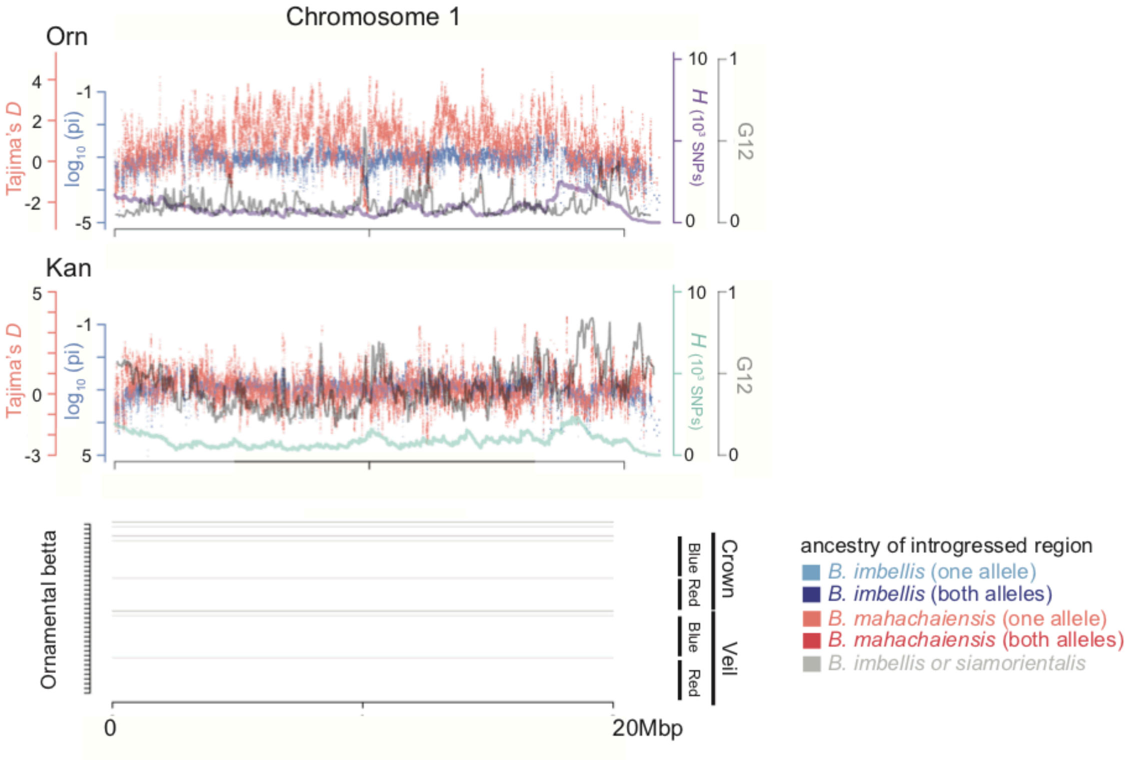
Chromosome 1
Orn
Tajima's D
log10 (pi)
H (10³ SNPs)
G12
Kan
Ornamental betta
Crown
Veil
Blue
Red
ancestry of introgressed region
B. imbellis (one allele)
B. imbellis (both alleles)
B. mahachaiensis (one allele)
B. mahachaiensis (both alleles)
B. imbellis or siamorientalis
0
20Mbp

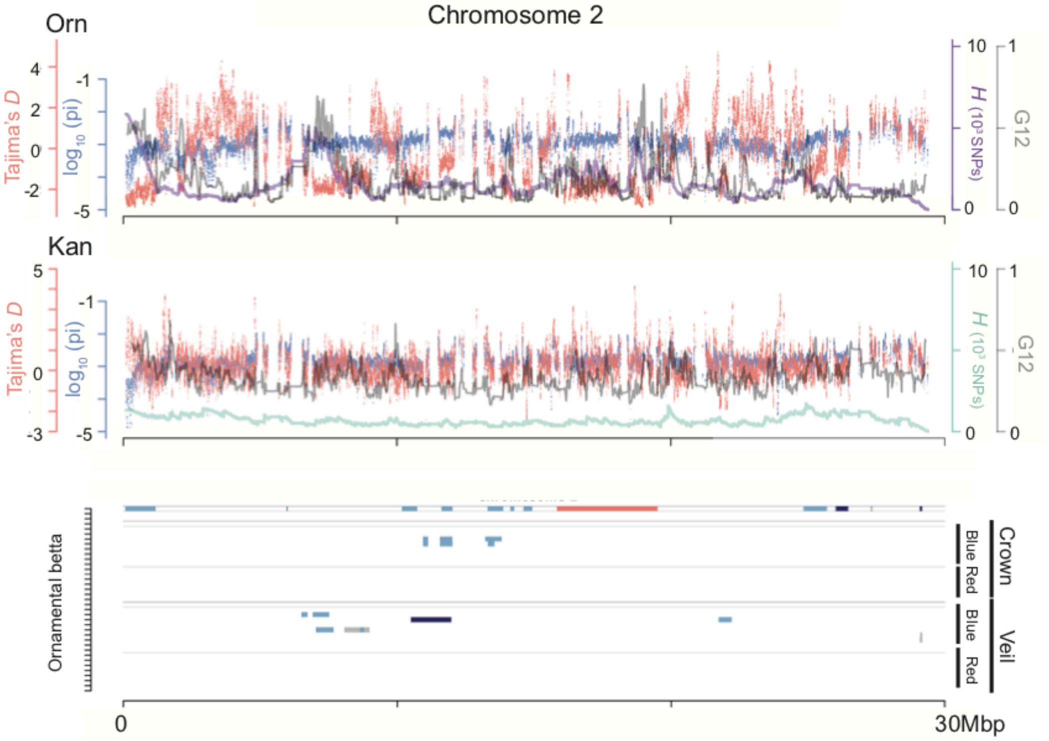
Chromosome 2
Orn
Tajima's D
log10 (pi)
H (10³ SNPs)
G12
Kan
Ornamental betta
Crown
Veil
Blue
Red
0
30Mbp

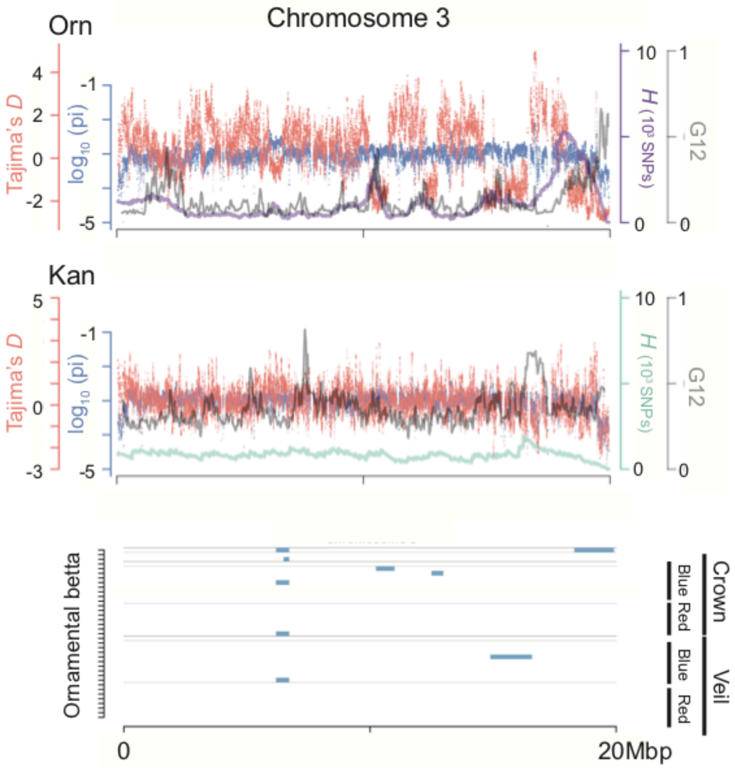
Chromosome 3
Orn
Tajima's D
log10 (pi)
H (10³ SNPs)
G12
Kan
Ornamental betta
Crown
Veil
Blue
Red
0
20Mbp

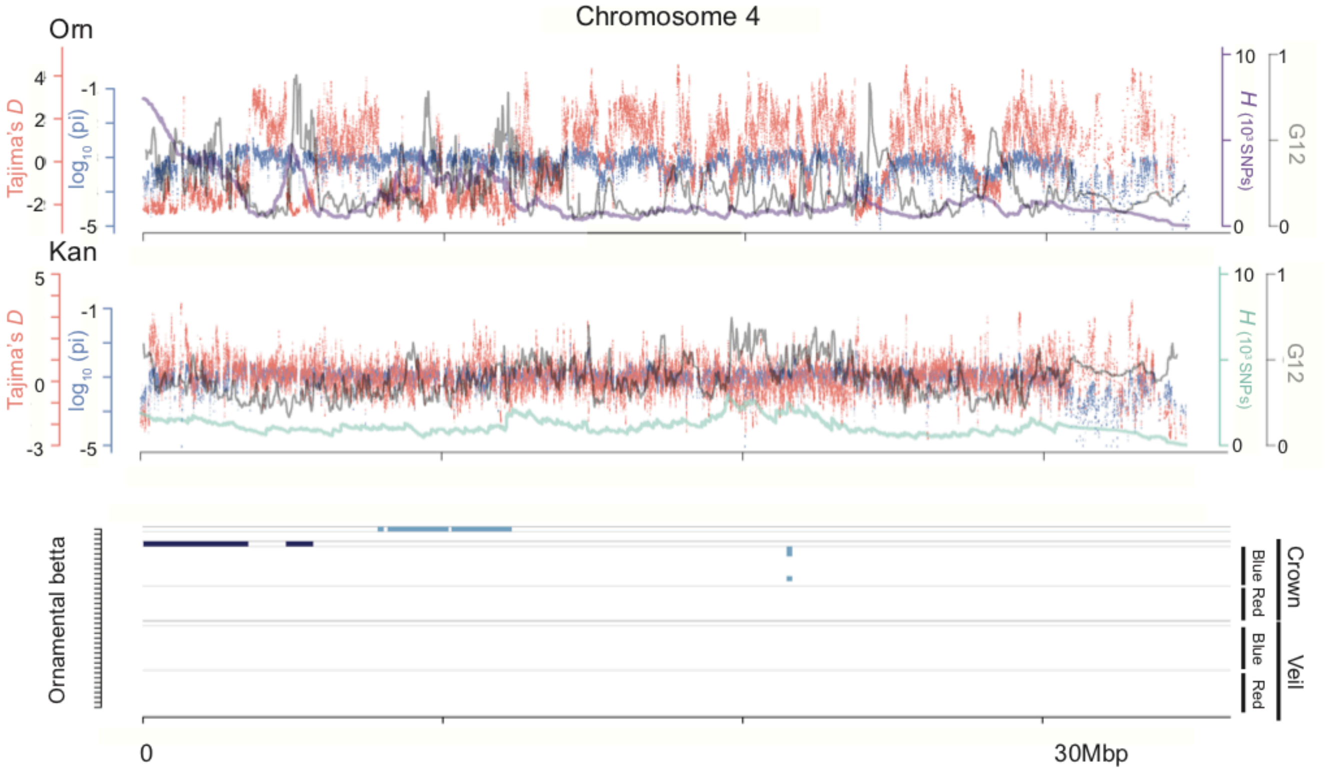
Chromosome 4
Orn
Tajima's D
log10 (pi)
H (10³ SNPs)
G12
Kan
Ornamental betta
Crown
Veil
Blue
Red
0
30Mbp

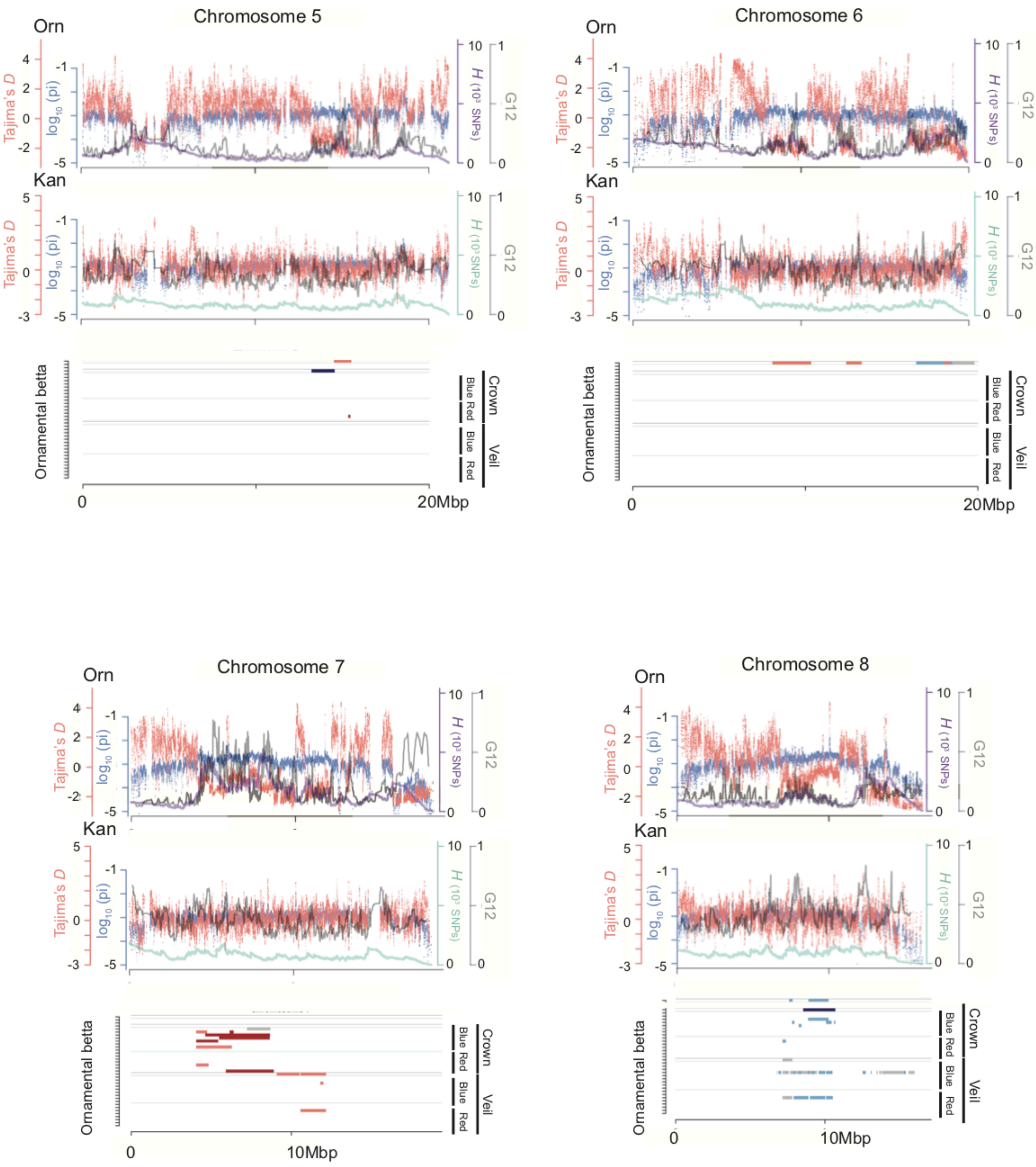
Chromosome 5
Orn
Kan
Tajima's D
log10 (pi)
H (10³ SNPs)
G12
Ornamental betta
Crown
Veil
Blue
Red
0
20Mbp
Chromosome 6
Orn
Kan
Tajima's D
log10 (pi)
H (10³ SNPs)
G12
Ornamental betta
Crown
Veil
Blue
Red
0
20Mbp
Chromosome 7
Orn
Kan
Tajima's D
log10 (pi)
H (10³ SNPs)
G12
Ornamental betta
Crown
Veil
Blue
Red
0
10Mbp
Chromosome 8
Orn
Kan
Tajima's D
log10 (pi)
H (10³ SNPs)
G12
Ornamental betta
Crown
Veil
Blue
Red
0
10Mbp

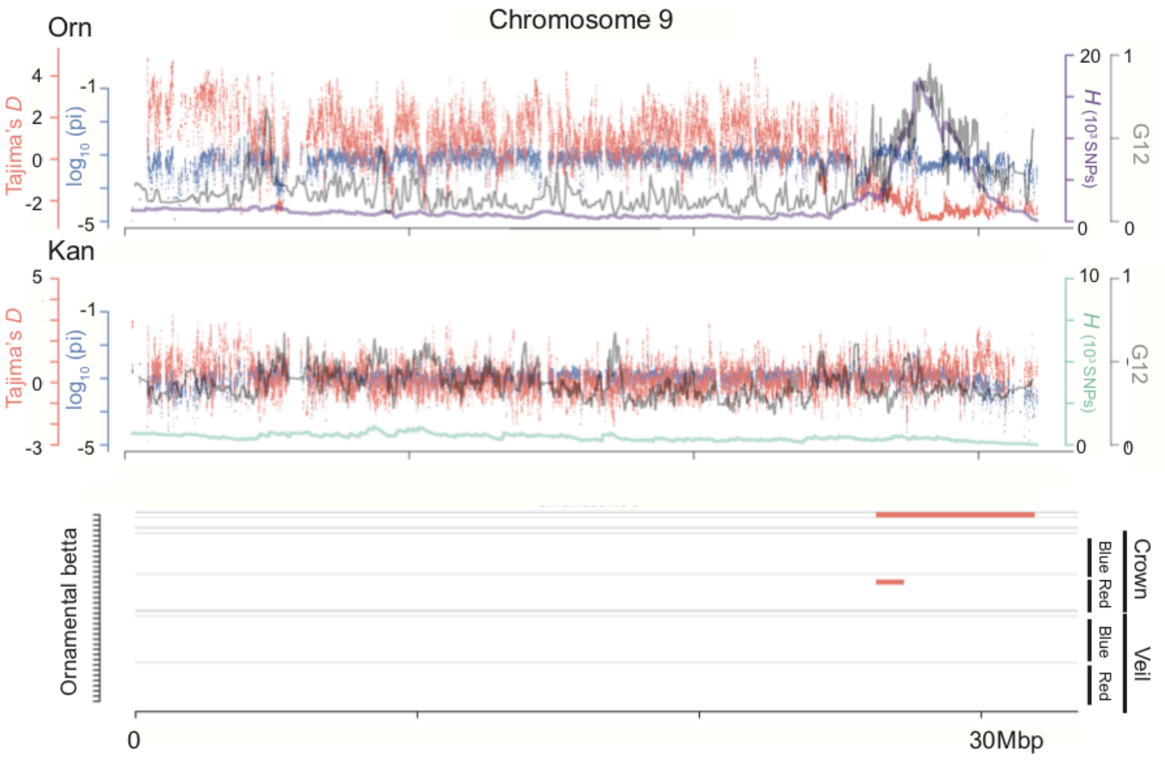
Chromosome 9
Orn
Kan
Tajima's D
log10 (pi)
H (10³SNPs)
G12
Ornamental betta
Crown
Veil
Blue
Red
0
30Mbp

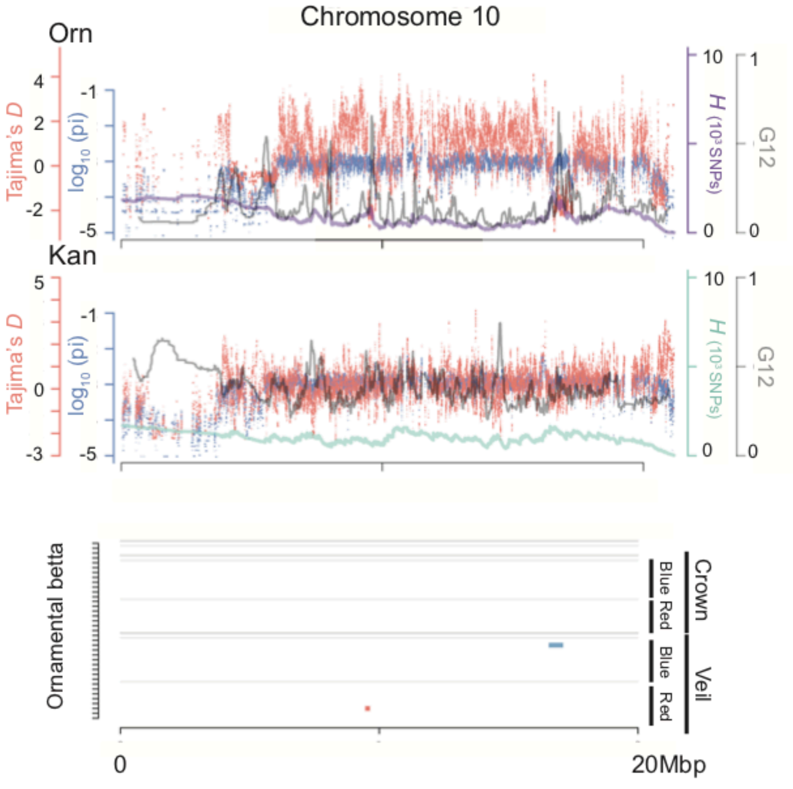
Chromosome 10
Orn
Kan
Tajima's D
log10 (pi)
H (10³SNPs)
G12
Ornamental betta
Crown
Veil
Blue
Red
0
20Mbp

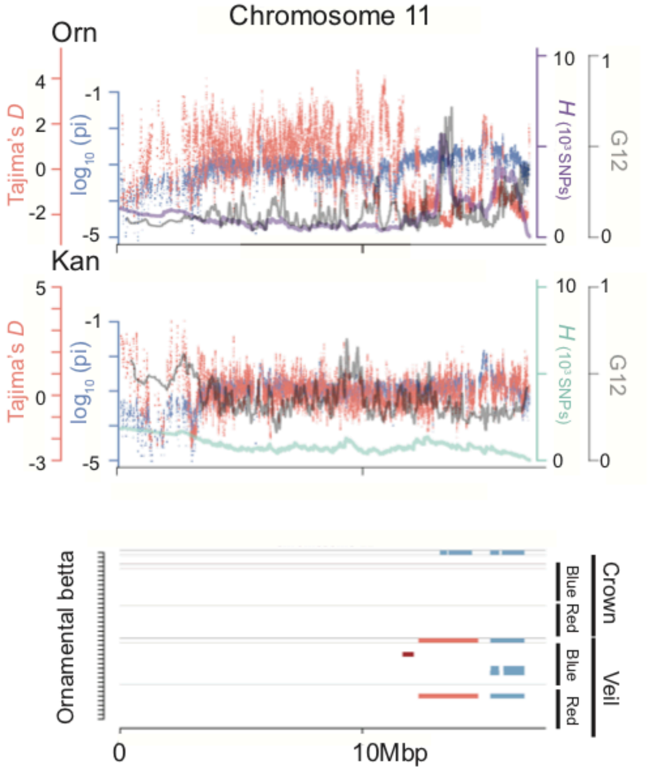
Chromosome 11
Orn
Kan
Tajima's D
log10 (pi)
H (10³SNPs)
G12
Ornamental betta
Crown
Veil
Blue
Red
0
10Mbp

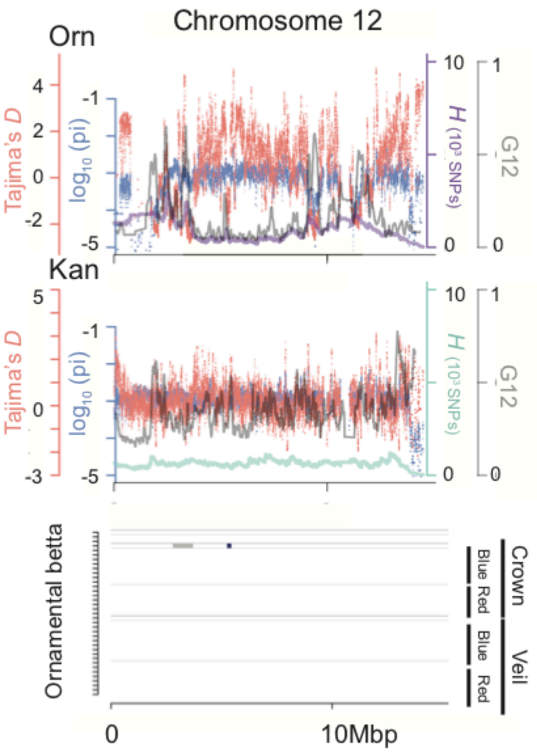

Chromosome 12
Orn
Kan
Tajima's D
log10 (pi)
H (10³ SNPs)
G12
Ornamental betta
Crown
Veil
Blue
Red
0
10Mbp

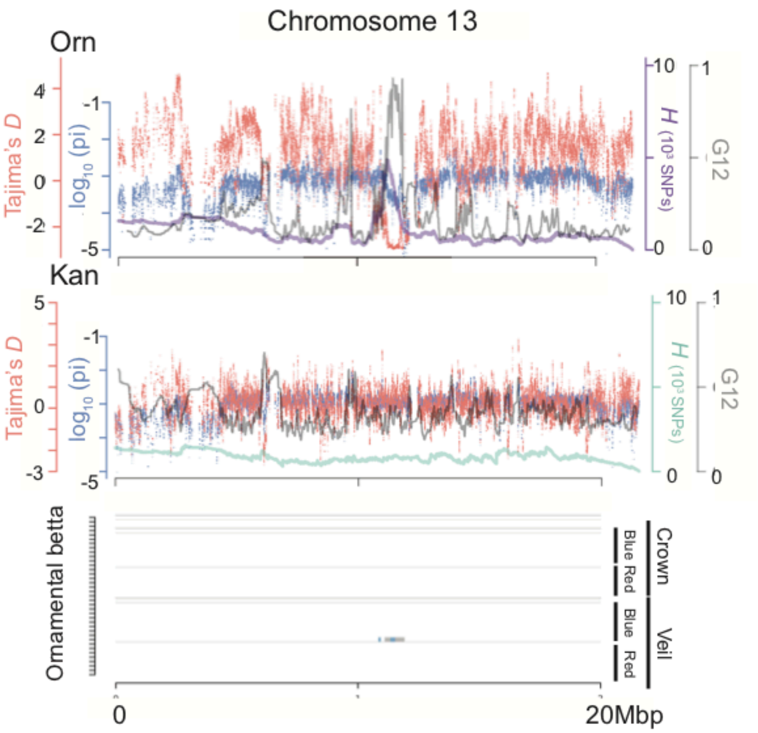

Chromosome 13
Orn
Kan
Tajima's D
log10 (pi)
H (10³ SNPs)
G12
Ornamental betta
Crown
Veil
Blue
Red
0
20Mbp

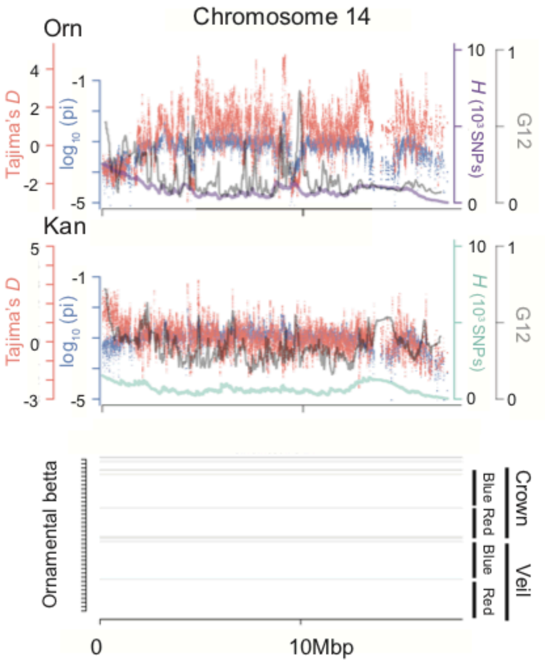

Chromosome 14
Orn
Kan
Tajima's D
log10 (pi)
H (10³ SNPs)
G12
Ornamental betta
Crown
Veil
Blue
Red
0
10Mbp

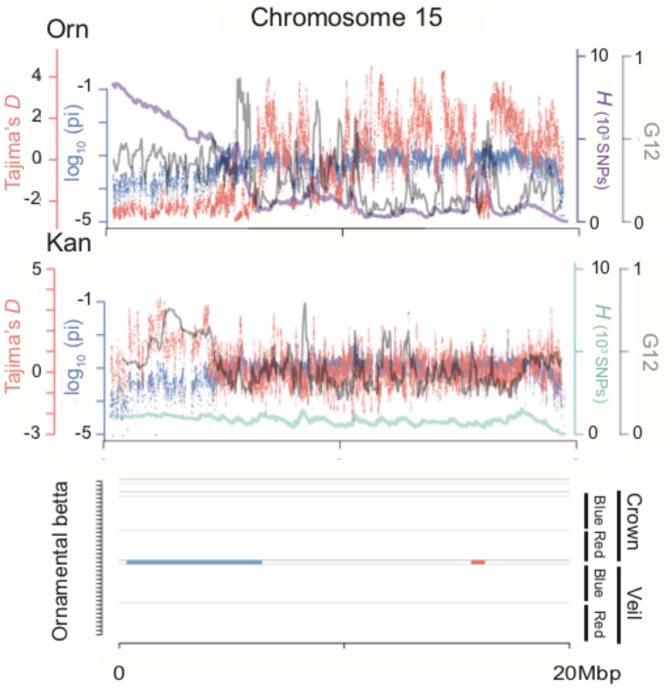

Chromosome 15
Orn
Kan
Tajima's D
log10 (pi)
H (10³ SNPs)
G12
Ornamental betta
Crown
Veil
Blue
Red
0
20Mbp

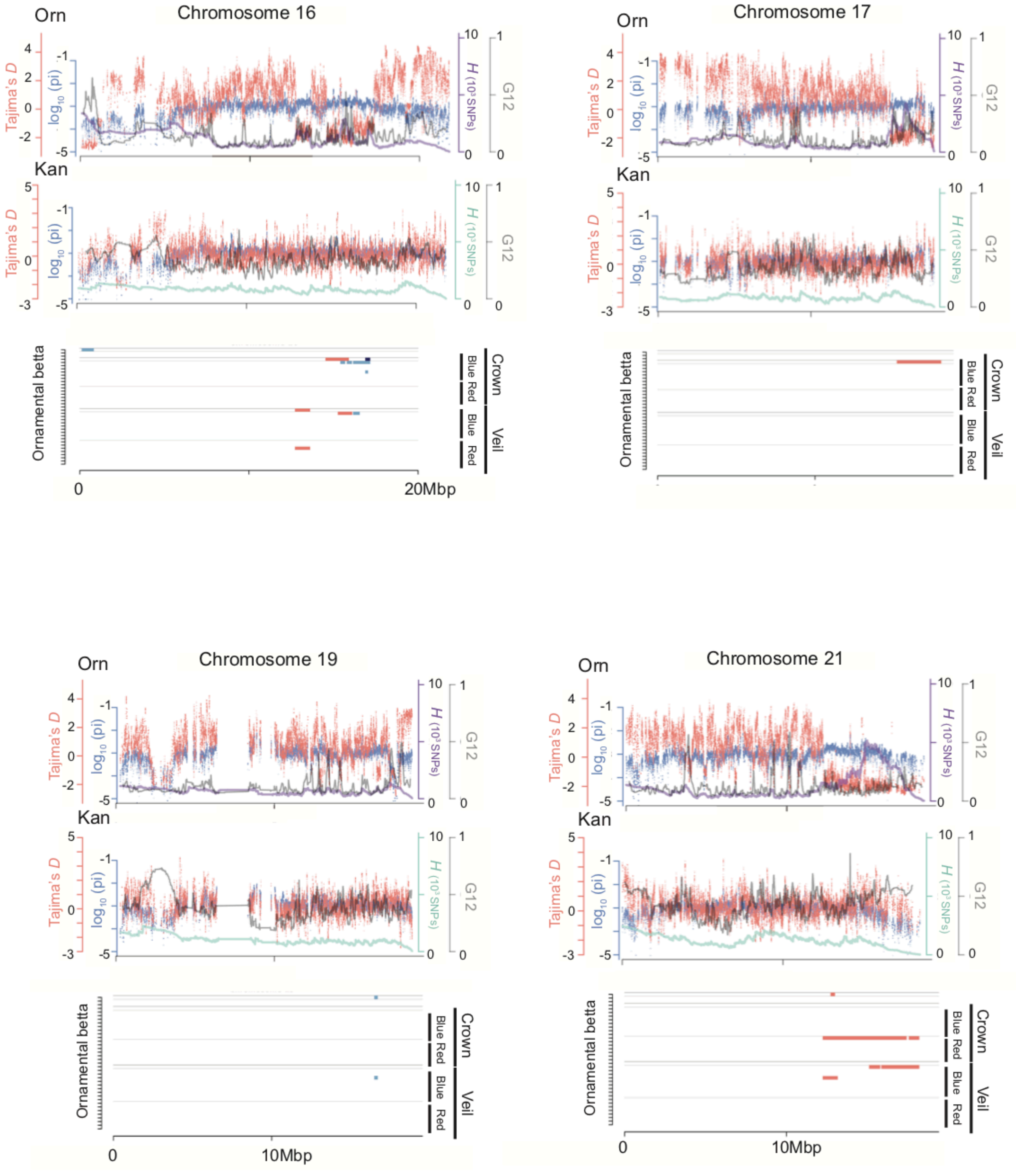

Chromosome 16
Chromosome 17
Chromosome 19
Chromosome 21
Orn
Kan
Tajima's D
log10 (pi)
H (10³SNPs)
G12
Ornamental betta
Crown
Veil
Blue
Red
0
20Mbp
10Mbp

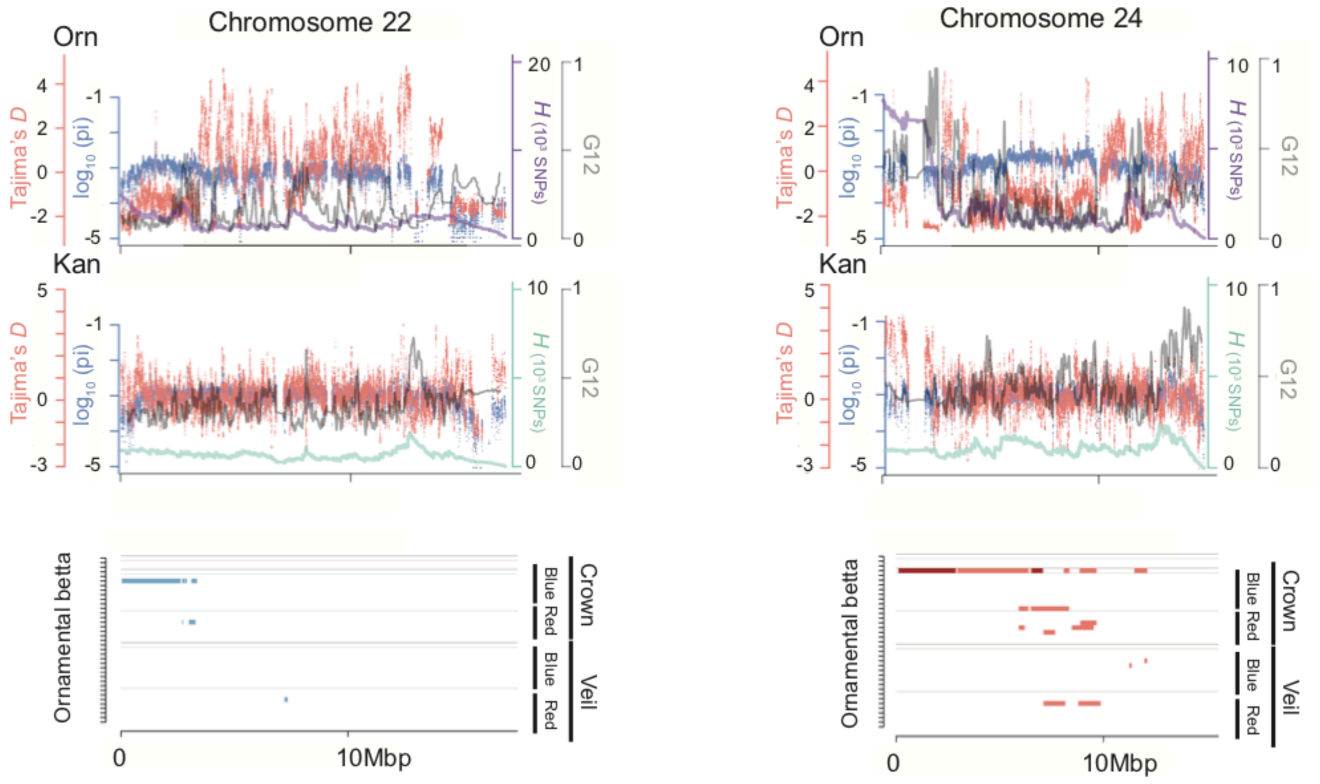

Chromosome 22
Orn
Kan
Tajima's D
log10 (pi)
H (10³ SNPs)
G12
Ornamental betta
Blue
Red
Crown
Veil
0
10Mbp
Chromosome 24

Fig. S12. Genome diversity and selection scans of ornamental betta and wild *B. splendens*. Per chromosome plots of nucleotide diversity (pi) and Tajima's *D* in 10-kb windows with 1-kb slide, as well as *H*-scan and G12 in ornamental (top panel of each chromosome) and wild *B. splendens* from Kanchanaburi (middle panel). Bottom panel: plot of regions with f_{dM}>0.2 per ornamental betta where p1 is the ornamental betta population and p3 is *imbellis* (red) or *mahachaiensis* (blue) populations ordered by fin morphology. Each row represents an ornamental individual. The three samples above crown tails are half-moon. Lighter shaded regions indicate introgression of one allele from either *B. imbellis* or *B. mahachaiensis*. Darker shading denotes introgression of both alleles. Grey denotes introgression from either *imbellis* or *siamorientalis*.

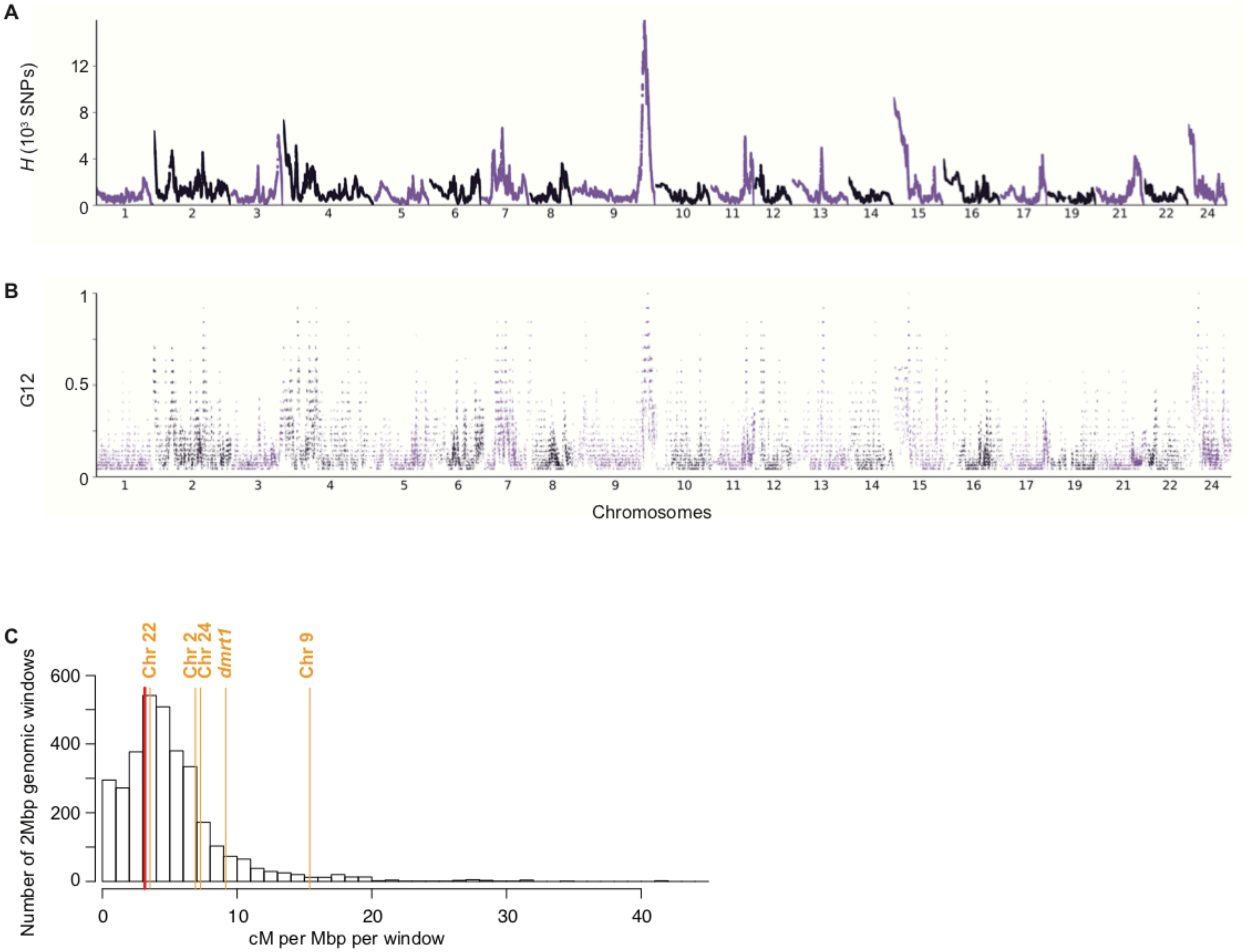

A
H (10³ SNPs)
0
4
8
12
1 2 3 4 5 6 7 8 9 10 11 12 13 14 15 16 17 19 21 22 24
B
G12
0
0.5
1
1 2 3 4 5 6 7 8 9 10 11 12 13 14 15 16 17 19 21 22 24
Chromosomes
C
Chr 22
Chr 2
Chr 24
dmrt1
Chr 9
Number of 2Mbp genomic windows
0
200
400
600
0
10
20
30
40
cM per Mbp per window

Fig. S13. *H*-scan and G12 selection scans across ornamental subsets.

A, Genome-wide *H*-scan of randomly sampled 24 independent ornamental bettas. **B**, Genome-wide G12 of randomly sampled 24 independent ornamental bettas. **C**, Distribution of centimorgans (cM) per megabase (Mbp) in 2 Mbp sliding windows using the intercross between a male (*dmrt1_XY*) and a female (*dmrt1_XX*) ornamental splendens (n=211 F2 individuals). Yellow lines denote the mean cM per Mbp in windows overlapping the selection peaks from Figure 2. Red line denotes the 30th percentile of the distribution.

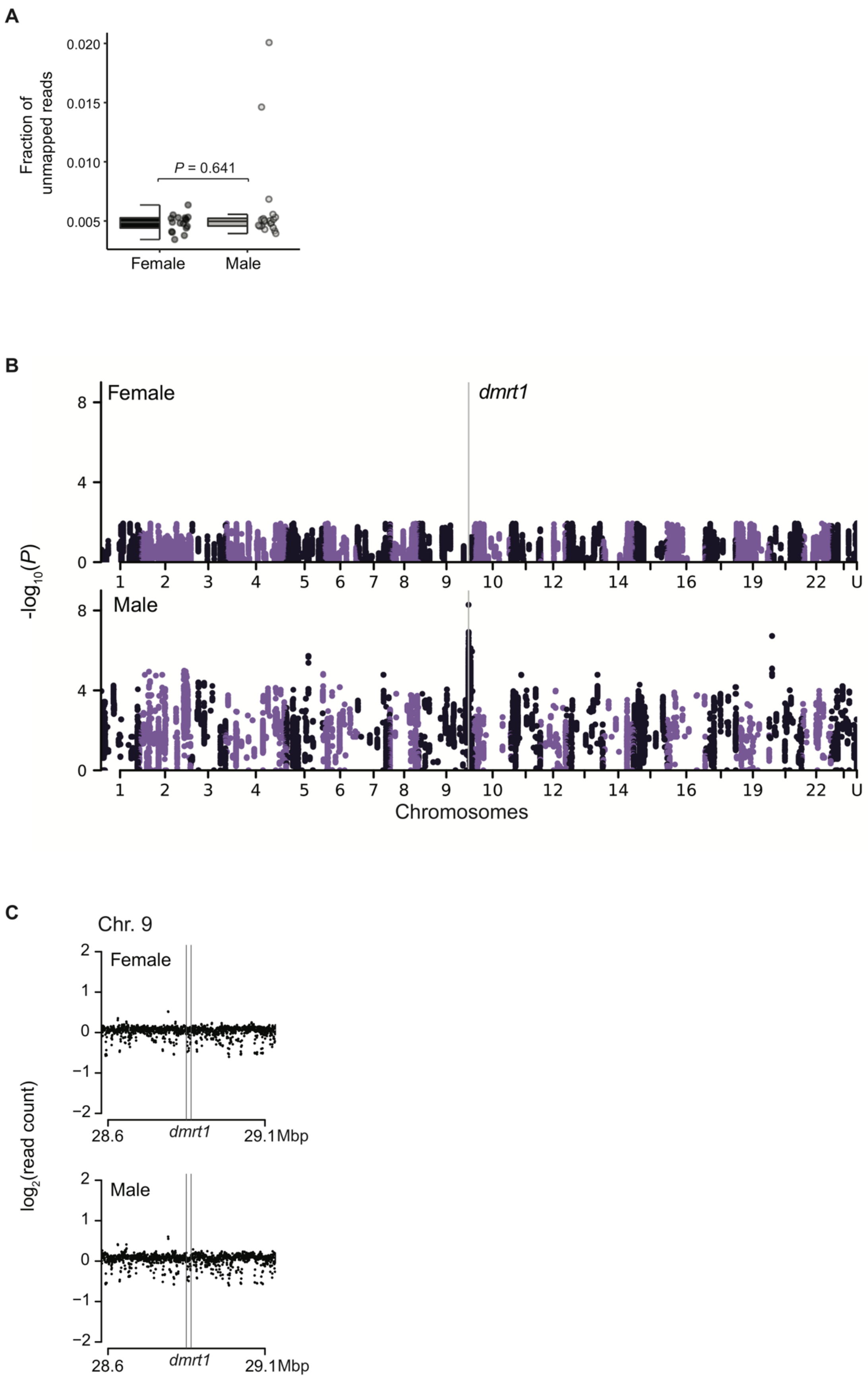

A
Fraction of unmapped reads
0.020
0.015
0.010
0.005
P = 0.641
Female
Male
B
Female
dmrt1
Male
-log10(P)
8
4
0
1
2
3
4
5
6
7
8
9
10
12
14
16
19
22
U
Chromosomes
C
Chr. 9
Female
Male
log2(read count)
2
1
0
−1
−2
28.6
dmrt1
29.1Mbp

Fig. S14. K-mer and sequencing read-depth differences across sexes.

A, Number of unmapped over total reads across sex. *P*-value by Mann-Whitney U test. **B**, k-mer genome-wide association plot with unmapped assembled contigs (U) placed at the after chromosome 24. **C**, Average log_2-normalized read depth in 1-kb bins with 500-bp slide across *dmrt1* on chromosome 9 for female and male ornamental betta.

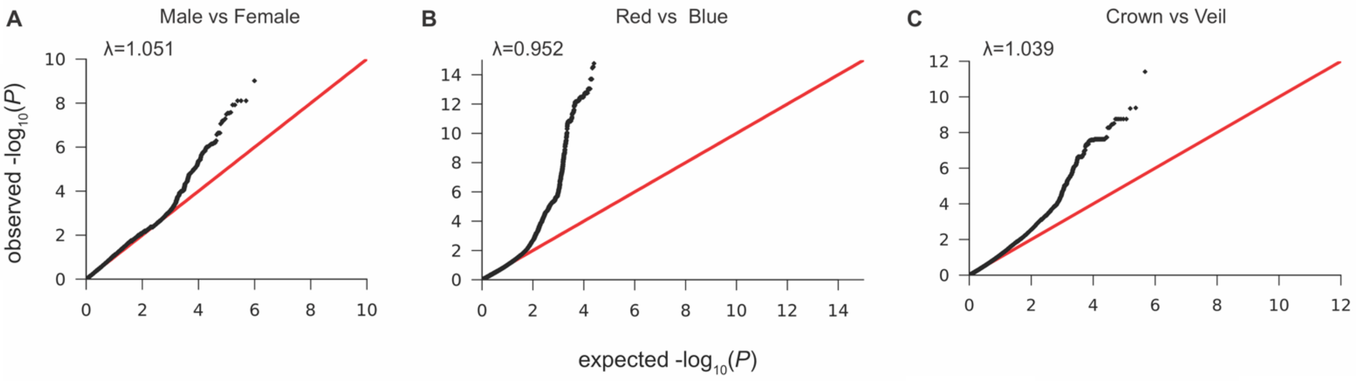

A
Male vs Female
λ=1.051
B
Red vs Blue
λ=0.952
C
Crown vs Veil
λ=1.039
observed -log10(P)
expected -log10(P)

Fig. S15. Q-Q plots for GWAS.

A, Q-Q plot for GWAS of Sex in Fig 3A. **B,** Q-Q plot for GWAS of red vs blue coloration in Fig. 4A. **C,** Q-Q plot for GWAS of crown vs veil fin types in Fig 5A. **A-C,** lambda represents the genomic inflation factor

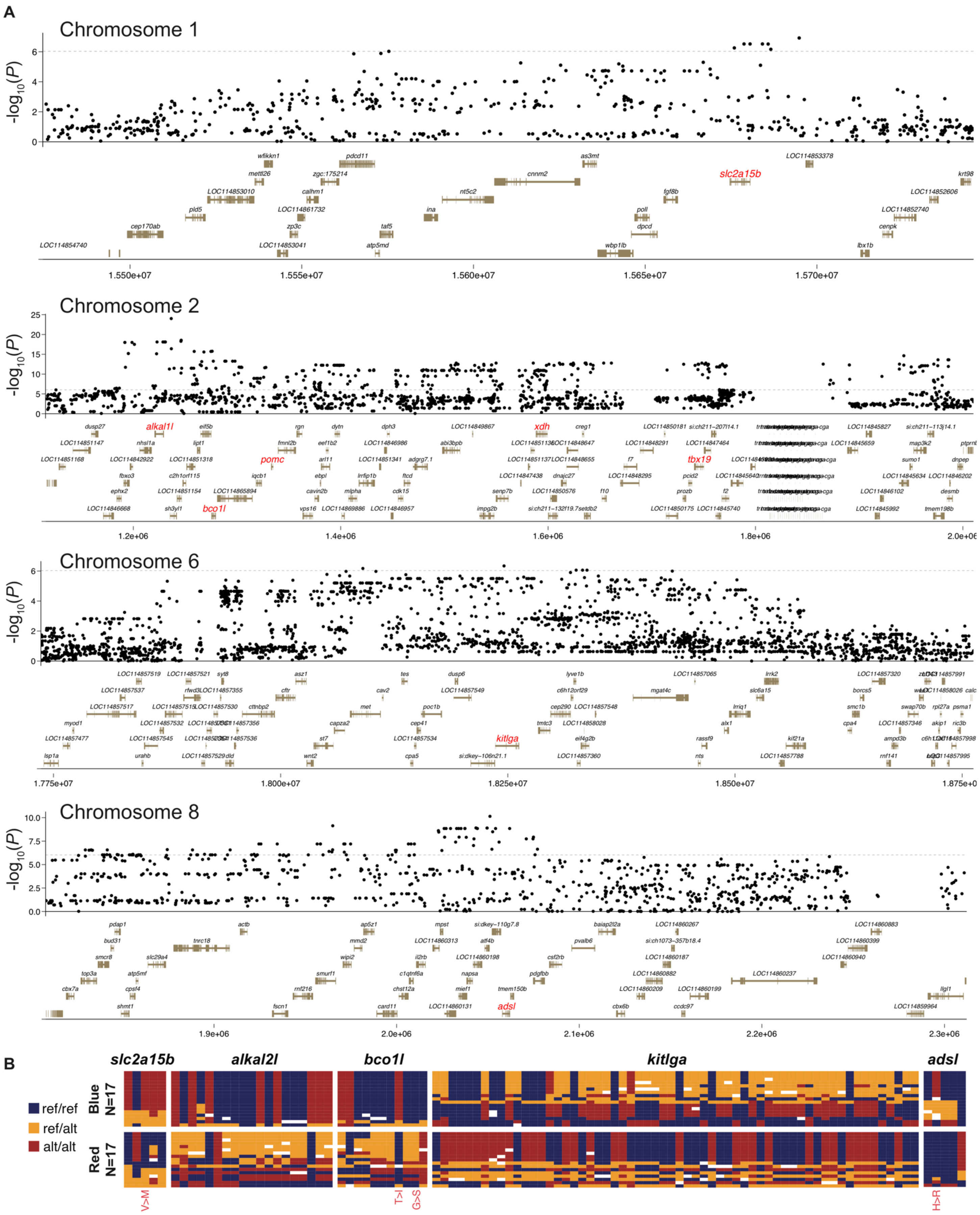
A
Chromosome 1
-log10(P)
slc2a15b
Chromosome 2
alkal1l
pomc
bco1l
xdh
tbx19
Chromosome 6
kitlga
Chromosome 8
adsl
B
slc2a15b
alkal2l
bco1l
kitlga
adsl
ref/ref
ref/alt
alt/alt
Blue N=17
Red N=17
V>M
T>I
G>S
H>R

Fig. S16. Extended red-blue GWAS peak plots.

A, Expanded view of the red-blue color GWAS peaks with gene annotations across chromosomes 1, 2, 6, and 8. Candidate genes with high -log10(*P*) values are highlighted in red. **B**, Genotypes of red and blue fish included in GWAS across genes of interest. Missense SNPs are annotated in red.

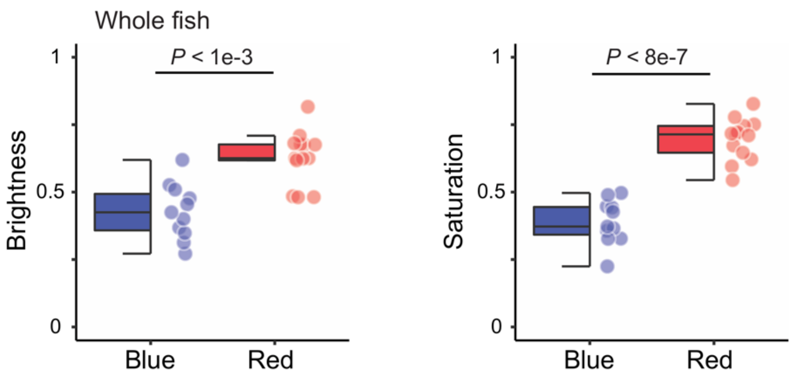
Whole fish
P < 1e-3
Brightness
1
0.5
0
Blue
Red
P < 8e-7
Saturation
1
0.5
0
Blue
Red

Fig. S17. Coloration of red and blue P0 Fish.

Average brightness and saturation in red and blue P0 (cross founders) fish. *P*-value by Mann-Whitney U test.

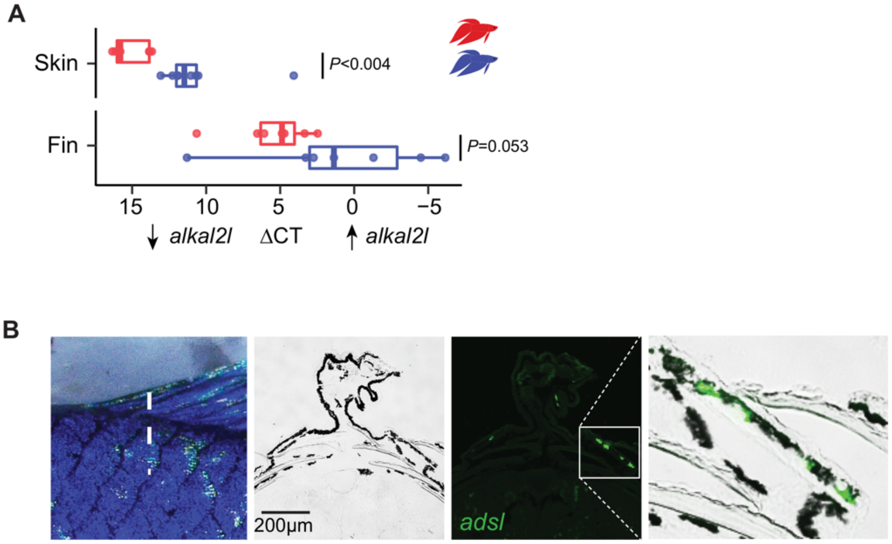
A
Skin
Fin
P<0.004
P=0.053
15
10
5
0
−5
↓ alkal2l
ΔCT
↑ alkal2l
B
200µm
adsl

Fig. S18. Expression of genes associated with variation in ornamental betta coloration.
A, *alkal2l* mRNA expression relative to *gapdh* of body skins and caudal fins of red and royal blue fish. *P*-value by Wilcoxon rank-sum test. **B**, Left to right: Photograph of skin of a royal blue fish, position of sectioned skin denoted by dashed line; skin section under bright-field illumination; fluorescence in situ hybridization for *adsl* in green; merged overlay of bright-field image and *adsl* in situ

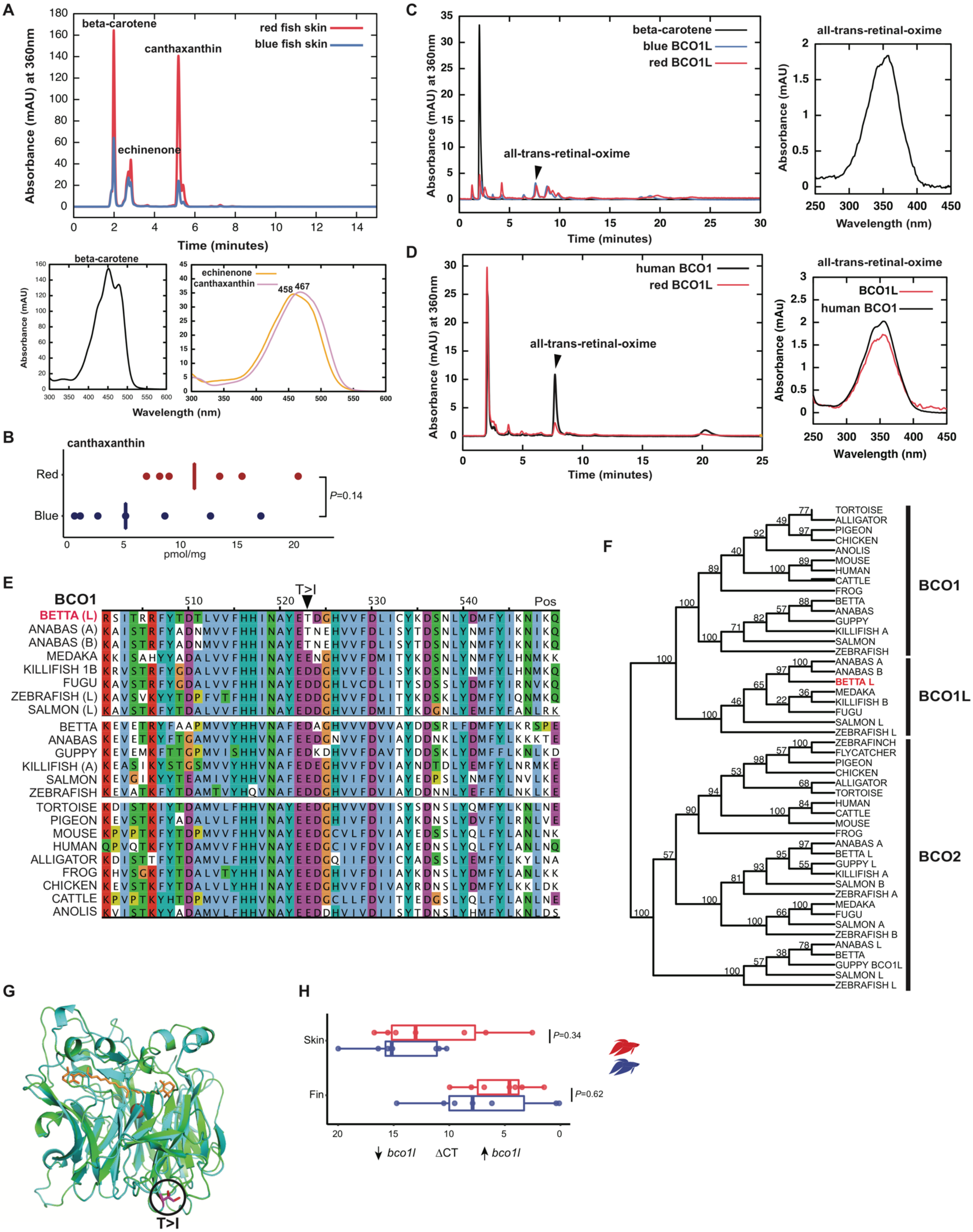

A
Absorbance (mAU) at 360nm
Time (minutes)
beta-carotene
echinenone
canthaxanthin
red fish skin
blue fish skin
beta-carotene
Absorbance (mAU)
echinenone
canthaxanthin
458 467
Wavelength (nm)
B
canthaxanthin
Red
Blue
P=0.14
pmol/mg
C
Absorbance (mAU) at 360nm
beta-carotene
blue BCO1L
red BCO1L
all-trans-retinal-oxime
Time (minutes)
all-trans-retinal-oxime
Absorbance (mAU)
Wavelength (nm)
D
Absorbance (mAU) at 360nm
human BCO1
red BCO1L
all-trans-retinal-oxime
Time (minutes)
all-trans-retinal-oxime
BCO1L
human BCO1
Absorbance (mAu)
Wavelength (nm)
E
BCO1
T>I
510
520
530
540
Pos
BETTA (L)
ANABAS (A)
ANABAS (B)
MEDAKA
KILLIFISH 1B
FUGU
ZEBRAFISH (L)
SALMON (L)
BETTA
ANABAS
GUPPY
KILLIFISH (A)
SALMON
ZEBRAFISH
TORTOISE
PIGEON
MOUSE
HUMAN
ALLIGATOR
FROG
CHICKEN
CATTLE
ANOLIS
F
TORTOISE
ALLIGATOR
PIGEON
CHICKEN
ANOLIS
MOUSE
HUMAN
CATTLE
FROG
BETTA
ANABAS
GUPPY
KILLIFISH A
SALMON
ZEBRAFISH
ANABAS A
ANABAS B
BETTA L
MEDAKA
KILLIFISH B
FUGU
SALMON L
ZEBRAFISH L
ZEBRAFINCH
FLYCATCHER
PIGEON
CHICKEN
ALLIGATOR
TORTOISE
HUMAN
CATTLE
MOUSE
FROG
ANABAS A
BETTA L
GUPPY L
KILLIFISH A
SALMON B
ZEBRAFISH A
MEDAKA
FUGU
SALMON A
ZEBRAFISH B
ANABAS L
BETTA
GUPPY BCO1L
SALMON L
ZEBRAFISH L
BCO1
BCO1L
BCO2
G
T>I
H
Skin
Fin
P=0.34
P=0.62
bco1l
ΔCT
bco1l

Fig. S19. Carotenoid and β-carotene oxygenase 1-like biochemistry.

A, HPLC trace of extracts from red and blue ornamental betta skins. **B**, Concentration of canthaxanthin in red (n=6) and blue (n=6) ornamental betta skin. *P*-value by Mann-Whitney U test. **C**, HPLC trace of lipid extracts from *E. coli* strain that accumulates β-carotene expressing the recombinant MBP-BCO1L red and blue fusion proteins. **D**, HPLC trace of products of the purified hBCO1 and red-allele BCO1L enzymes, showing the 15,15′-dioxygenase product all-trans-retinal-oxime. **E**, Multi-species amino acid alignment of BCO1L using MAFFT, color coded with the Clustal X color scheme with consensus sequence and frequency below. **F**, Neighbor-joining tree of 100 bootstraps of the BCO amino acid alignment across vertebrates. **G**, Predicted monomer model of BCO1L. Orange shows β-carotene in the catalytic site, magenta indicates the location of the T>I (red, wildtype allele to blue allele) mutation. **H**, *bco1l* mRNA expression relative to *gapdh* of body skins and caudal fins of red and royal blue fish. *P*-value by Wilcoxon rank-sum test.

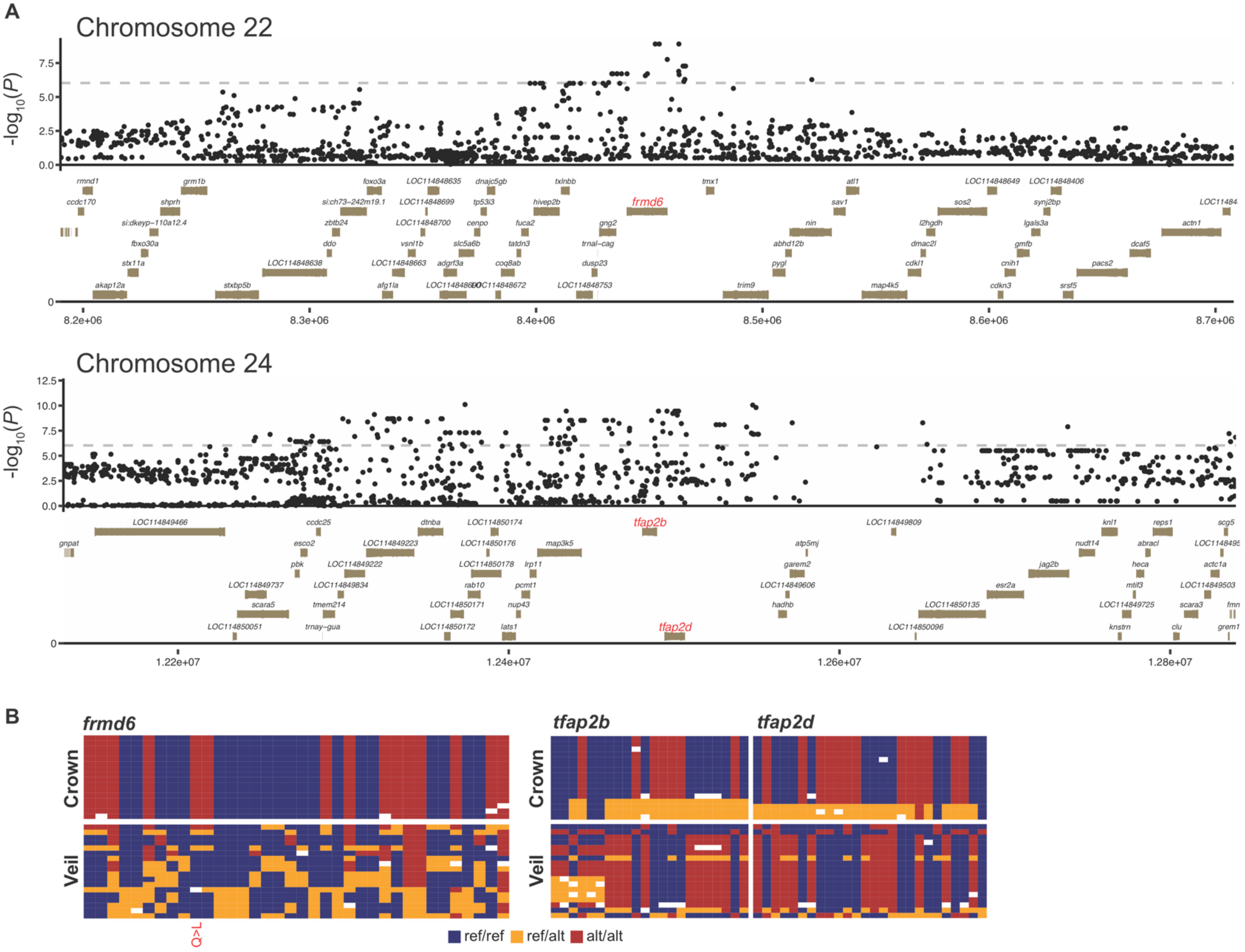

A
Chromosome 22
-log10(P)
frmd6
8.2e+06
8.3e+06
8.4e+06
8.5e+06
8.6e+06
8.7e+06
Chromosome 24
tfap2b
tfap2d
1.22e+07
1.24e+07
1.26e+07
1.28e+07
B
frmd6
tfap2b
tfap2d
Crown
Veil
Q>L
ref/ref
ref/alt
alt/alt

Fig. S20. Extended crown-veil fin GWAS and genotype plots.

A, Expanded view of the crown-veil fin GWAS peaks with gene annotations across Chromosomes 22 and 24. Candidate genes with high -log10(*P*) values are highlighted in red. **B**, Genotypes of crown and veil fish included in GWAS across genes of interest. Missense SNPs are annotated in red.

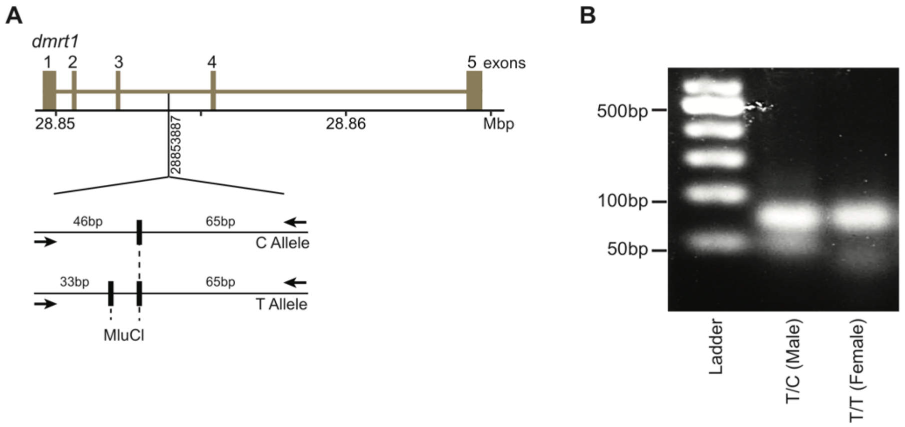
A
dmrt1
1
2
3
4
5
exons
28.85
28.86
Mbp
28853887
46bp
65bp
C Allele
33bp
65bp
T Allele
MluCI
B
500bp
100bp
50bp
Ladder
T/C (Male)
T/T (Female)

Fig. S21. Restriction fragment length polymorphism (RFLP) genotyping.

A, Schematic of the MluCl restriction enzyme cut sites (bolded vertical black bars) and the lengths of the digested PCR product for alleles carrying either the T or C variant at chromosome 9: 288533887. **B**, 2.5% agarose gel electrophoresis of digested fragments from a male *dmrt1_XY* carrying the T/C genotype and female *dmrt1_XX* carrying the T/T genotype.

Table S1. Sample metadata.

Metadata information of the whole-genome sequenced samples used in population genetic analyses. Provided as a supplementary excel file.

Table S2. Chromosomal locations of loci and candidate genes affecting phenotypic traits in betta.

fBetSpl5.3 genomic coordinates of genes and genomic loci associated with phenotypic traits described in this study and Wang et al. 2021 (*13*).

Phenotype	Candidate gene	Gene name	Chromosome	Start (bp)	End (bp)
sex determination	dmrt1	doublesex and mab-3 related transcription factor 1	Chromosome 9	28,849,542	28,864,704
iridiophore differentiation	alkal2l	LOC114851404; ALK and LTK ligand 1-like (renamed to alkal2l based on sequence similarity to alkal2)	Chromosome 2	1,220,413	1,229,520
red hue in body; proportion of red in body and fins	bco1l	beta-carotene oxygenase 1, like	Chromosome 2	1,275,344	1,279,650
iridesence; guanine snythesis	adsl	adenylosuccinate lyase	Chromosome 8	2,057,799	2,062,135
red saturation in fins	slc2a15b	solute carrier family 2 member 15b	Chromosome 1	15,674,584	15,680,614
black spread in head	kitlga	kit ligand a	Chromosome 6	18,236,164	18,262,486
crowning in anal fin	frmd6	FERM domain containing 6	Chromosome 22	8,439,990	8,458,042
crowning in dorsal fin	tfap2b	transcription factor AP-2 beta	Chromosome 24	12,480,700	12,489,712
crowning in dorsal fin	tfap2d	transcription factor AP-2 delta	Chromosome 24	12,494,194	12,506,588
albinism (*13*)	mitfa	melanocyte inducing transcription factor a	Chromosome 5	2,408,544	2,413,248
spotting in dorsal fin (QTL) (*13*)	Unknown	-	Chromosome 8	1,600,000	1,870,000
red pigmentation in caudal tail (QTL) (*13*)	Unknown	-	Chromosome 1	Not available	Not available
red pigmentation in head (QTL) (*13*)	Unknown	-	Chromosome 5	Not available	Not available
elephant ear in pectoral fin (*13*)	kcnh8	potassium voltage-gated channel, subfamily H, member 8	Chromosome 11	7,753,376	7,794,461
double tail in caudal fin (*13*)	zic1	zic family member 1	Chromosome 4	18,982,259	18,985,650
double tail in caudal fin (*13*)	zic4	zic family member 4	Chromosome 4	18,988,191	18,993,322

Table S3. Primer sequences.

Primer sequences used for genotyping, for qPCR, and for generation of in situ probes.

Type	Gene	SNP Chromosome: position ref>alt	Primer Sequences (5'-*Tn5ME* …-3')	Amplicon Length (bp)
Sequence-based genotyping	*dmrt1*	Chromosome 9: 28850248 G>T	*For: 5'- GTCTCGTGGGCTCGGAGATGTGTATAAGAGACAG* TAACGCCGTAGCGTTAGCTT-3'	
			Rev: 5'- TCGTCGGCAGCGTCAGATGTGTATAAGAGACAG CCTCATAATTCACAAAGGTGAAAG-3'	201
		Chromosome 9: 28858562 A>G	*For: 5'- GTCTCGTGGGCTCGGAGATGTGTATAAGAGACAG* CGATTTTCCTGCCTTAATCC-3'	
			Rev: 5'- TCGTCGGCAGCGTCAGATGTGTATAAGAGACAG CTTTCCGACGGCAGTGTG-3'	299
		Chromosome 9: 28863849 T>C	*For: 5'- TCGTCGGCAGCGTCAGATGTGTATAAGAGACAG* AAATCTGAGCCTTTATTTCTACACA-3'	
			Rev: 5'- GTCTCGTGGGCTCGGAGATGTGTATAAGAGACAG CGGAATCAGGCACAGTAAAA-3'	322
		Chromosome 9: 28864161 G>A	*For: 5'- GTCTCGTGGGCTCGGAGATGTGTATAAGAGACAG* AGCACCAACAGCTGTCCTTT-3'	
			Rev: 5'- TCGTCGGCAGCGTCAGATGTGTATAAGAGACAG CTGCAGGTCATGGTGGAGT-3'	299
		Chromosome 9: 28867677 G>A	*For: 5'- GTCTCGTGGGCTCGGAGATGTGTATAAGAGACAG* CAAATGCAGCCTAATTTTTCA-3'	
			Rev: 5'- TCGTCGGCAGCGTCAGATGTGTATAAGAGACAG GGCGTGACACCGAGAAAC-3'	262
RFLP-based genotyping		Chromosome 9: 288533887 T>C	*For: 5'-*ACTCATCATCTTCACAGGAAGCA *-3'*	
			*Rev: 5'-*AAAGTGGGGTTTAGGGCAGG*'-3'*	112
Allele-specific expression amplicon		Chromosome 9: 28864161 G>A	For: 5'-TGCTGCTGGAAACCTCCTAT -3'	
			Rev: 5'-CTGCAGGTCATGGTGGAGT -3'	316
qPCR	*alkal2l*	-	For-5'-GCGTGAAACCACTGGAGAGAG-3'	
			For-5'-CCCTGGTGCTGTGGTAGATTC-3'	159
	gapdh	-	For-5'-AGGACAGACTGAGGCCTTCTC-3'	
			For-5'-TCACCAGACGACCAATGCG-3'	101
	bco1l	-	For-5'-ACCTACAACATGGGCACCAC-3'	
			For-5'-CTCAGCGCTGGCTTCTTC-3'	107
in-situ	*adsl*	-	For-5'-TGAGGAGCCATTTGAGAAAGAAC-3'	
			For-5'-GACCTACAGTCGTAAGGTGGAC-3'	713

Table S4. Variant filtering.

B. splendens species complex species and population-specific regions filter criteria for Total genome size (chromosomes only) is 435314815 bp.

	**Filters**	**Genome (bp) removed (Chromosomes only)**	**% Genome removed (Chromosomes only)**
B. splendens species complex	SNPable filter	31564934	7.30%
	Transposable Element (TE) filter	89388187	20.50%
	Read-coverage filter	99305522	22.80%
	Read-coverage + SNPable filter + TE filter	127909927	29.4%; 98% overlap of SNPable to read-coverage; 74.1% overlap of TE filter to read-coverage
B. splendens	Read-coverage + SNPable filter + TE filter	122306715	28.1%; 93.5% overlap of SNPable to read-coverage; 47.6% overlap of TE filter to read-coverage
Ornamental betta	Read-coverage + SNPable filter + TE filter	120969387	27.8%; 86.3% overlap of SNPable to Read-coverage; 39.1% overlap of TE filter to read-coverage
Wild *B. splendens*	Read-coverage + SNPable filter	120059452	27.5%; 91.6% overlap of SNPable to Read-coverage; 44.6% overlap of TE filter to read-coverage

REFERENCES AND NOTES

1. L. A. F. Frantz, D. G. Bradley, G. Larson, L. Orlando, Animal domestication in the era of ancient genomics. *Nat. Rev. Genet.* **21**, 449–460 (2020).

2. C.-J. Rubin, M. C. Zody, J. Eriksson, J. R. S. Meadows, E. Sherwood, M. T. Webster, L. Jiang, M. Ingman, T. Sharpe, S. Ka, F. Hallböök, F. Besnier, O. Carlborg, B. Bed'hom, M. Tixier-Boichard, P. Jensen, P. Siegel, K. Lindblad-Toh, L. Andersson, Whole-genome resequencing reveals loci under selection during chicken domestication. *Nature* **464**, 587–591 (2010).

3. P. Xu, X. Zhang, X. Wang, J. Li, G. Liu, Y. Kuang, J. Xu, X. Zheng, L. Ren, G. Wang, Y. Zhang, L. Huo, Z. Zhao, D. Cao, C. Lu, C. Li, Y. Zhou, Z. Liu, Z. Fan, G. Shan, X. Li, S. Wu, L. Song, G. Hou, Y. Jiang, Z. Jeney, D. Yu, L. Wang, C. Shao, L. Song, J. Sun, P. Ji, J. Wang, Q. Li, L. Xu, F. Sun, J. Feng, C. Wang, S. Wang, B. Wang, Y. Li, Y. Zhu, W. Xue, L. Zhao, J. Wang, Y. Gu, W. Lv, K. Wu, J. Xiao, J. Wu, Z. Zhang, J. Yu, X. Sun, Genome sequence and genetic diversity of the common carp. *Cyprinus carpio*. *Nat. Genet.* **46**, 1212–1219 (2014).

4. Z. Chen, Y. Omori, S. Koren, T. Shirokiya, T. Kuroda, A. Miyamoto, H. Wada, A. Fujiyama, A. Toyoda, S. Zhang, T. G. Wolfsberg, K. Kawakami, A. M. Phillippy; NISC Comparative Sequencing Program, J. C. Mullikin, S. M. Burgess, De novo assembly of the goldfish (*Carassius auratus*) genome and the evolution of genes after whole-genome duplication. *Sci. Adv.* **5**, eaav0547 (2019).

5. D. Chen, Q. Zhang, W. Tang, Z. Huang, G. Wang, Y. Wang, J. Shi, H. Xu, L. Lin, Z. Li, W. Chi, L. Huang, J. Xia, X. Zhang, L. Guo, Y. Wang, P. Ma, J. Tang, G. Zhou, M. Liu, F. Liu, X. Hua, B. Wang, Q. Shen, Q. Jiang, J. Lin, X. Chen, H. Wang, M. Dou, L. Liu, H. Pan, Y. Qi, B. Wu, J. Fang, Y. Zhou, W. Cen, W. He, Q. Zhang, T. Xue, G. Lin, W. Zhang, Z. Liu, L. Qu, A. Wang, Q. Ye, J. Chen, Y. Zhang, R. Ming, M. Van Montagu, H. Tang, Y. Van de Peer, Y. Chen, J. Zhang, The evolutionary origin and domestication history of goldfish (*Carassius auratus*). *Proc. Natl. Acad. Sci. U.S.A.* **117**, 29775–29785 (2020).

6. M. Bekoff, *Encyclopedia of Human-Animal Relationships* (Greenwood Press, 2007).

7. M. Brammah, *The Betta Bible* (CreateSpace Independent Publishing Platform, 2015).

8. L. Rüber, R. Britz, R. Zardoya, Molecular phylogenetics and evolutionary diversification of labyrinth fishes (Perciformes: *Anabantoidei*). *Syst. Biol.* **55**, 374–397 (2006).

9. N. Sriwattanarothai, D. Steinke, P. Ruenwongsa, R. Hanner, B. Panijpan, Molecular and morphological evidence supports the species status of the Mahachai fighter *Betta* sp. Mahachai and reveals new species of *Betta* from Thailand. *J. Fish Biol.* **77**, 414–424 (2010).

10. International Betta Congress, About *Betta splendens*. *IBC* (2020); https://ibcbettas.org/about-betta-splendens/.

11. G. Fan, J. Chan, K. Ma, B. Yang, H. Zhang, X. Yang, C. Shi, H. Chun-Hin Law, Z. Ren, Q. Xu, Q. Liu, J. Wang, W. Chen, L. Shao, D. Gonçalves, A. Ramos, S. D. Cardoso, M. Guo, J. Cai, X. Xu, J. Wang, H. Yang, X. Liu, Y. Wang, Chromosome-level reference genome of the Siamese fighting fish *Betta splendens,* a model species for the study of aggression. *Gigascience* **7**, giy087 (2018).

12. S. Prost, M. Petersen, M. Grethlein, S. J. Hahn, N. Kuschik-Maczollek, M. E. Olesiuk, J.-O. Reschke, T. E. Schmey, C. Zimmer, D. K. Gupta, T. Schell, R. Coimbra, J. De Raad, F. Lammers, S. Winter, A. Janke, Improving the chromosome-level genome assembly of the Siamese fighting fish (*Betta splendens*) in a university master's course. *G3* **10**, 2179–2183 (2020).

13. L. Wang, F. Sun, Z. Y. Wan, B. Ye, Y. Wen, H. Liu, Z. Yang, H. Pang, Z. Meng, B. Fan, Y. Alfiko, Y. Shen, B. Bai, M. S. Q. Lee, F. Piferrer, M. Schartl, A. Meyer, G. H. Yue, Genomic basis of striking fin shapes and colors in the fighting fish. *Mol. Biol. Evol.* **38**, 3383–3396 (2021).

14. K. Howe, W. Chow, J. Collins, S. Pelan, D.-L. Pointon, Y. Sims, J. Torrance, A. Tracey, J. Wood, Significantly improving the quality of genome assemblies through curation. *Gigascience* **10**, giaa153 (2021).

15. F.-S. Grazyna, D. Fopp-Bayat, M. Jankun, S. Krejszeff, A. Mamcarz, Note on the karyotype and NOR location of Siamese fighting fish *Betta splendens* (Perciformes, Osphronemidae). *Caryologia* **61**, 349–353 (2008).

16. A. Rhie, S. A. McCarthy, O. Fedrigo, J. Damas, G. Formenti, S. Koren, M. Uliano-Silva, W. Chow, A. Fungtammasan, G. L. Gedman, L. J. Cantin, F. Thibaud-Nissen, L. Haggerty, C. Lee, B. J. Ko, J.

Kim, I. Bista, M. Smith, B. Haase, J. Mountcastle, S. Winkler, S. Paez, J. Howard, S. C. Vernes, T. M. Lama, F. Grutzner, W. C. Warren, C. Balakrishnan, D. Burt, J. M. George, M. Biegler, D. Iorns, A. Digby, D. Eason, T. Edwards, M. Wilkinson, G. Turner, A. Meyer, A. F. Kautt, P. Franchini, H. William Detrich, H. Svardal, M. Wagner, G. J. P. Naylor, M. Pippel, M. Malinsky, M. Mooney, M. Simbirsky, B. T. Hannigan, T. Pesout, M. Houck, A. Misuraca, S. B. Kingan, R. Hall, Z. Kronenberg, J. Korlach, I. Sović, C. Dunn, Z. Ning, A. Hastie, J. Lee, S. Selvaraj, R. E. Green, N. H. Putnam, J. Ghurye, E. Garrison, Y. Sims, J. Collins, S. Pelan, J. Torrance, A. Tracey, J. Wood, D. Guan, S. E. London, D. F. Clayton, C. V. Mello, S. R. Friedrich, P. V. Lovell, E. Osipova, F. O. Al-Ajli, S. Secomandi, H. Kim, C. Theofanopoulou, Y. Zhou, R. S. Harris, K. D. Makova, P. Medvedev, J. Hoffman, P. Masterson, K. Clark, F. Martin, K. Howe, P. Flicek, B. P. Walenz, W. Kwak, H. Clawson, M. Diekhans, L. Nassar, B. Paten, R. H. S. Kraus, H. Lewin, A. J. Crawford, M. T. P. Gilbert, G. Zhang, B. Venkatesh, R. W. Murphy, K.-P. Koepfli, B. Shapiro, W. E. Johnson, F. Di Palma, T. Margues-Bonet, E. C. Teeling, T. Warnow, J. M. Graves, O. A. Ryder, D. Hausler, S. J. O'Brien, K. Howe, E. W. Myers, R. Durbin, A. M. Phillippy, E. D. Jarvis, Towards complete and error-free genome assemblies of all vertebrate species. *Nature* **592**, 737–746 (2021).

17. N. Patterson, P. Moorjani, Y. Luo, S. Mallick, N. Rohland, Y. Zhan, T. Genschoreck, T. Webster, D. Reich, Ancient admixture in human history. *Genetics* **192**, 1065–1093 (2012).

18. S. H. Martin, J. W. Davey, C. D. Jiggins, Evaluating the use of ABBA–BABA statistics to locate introgressed loci. *Mol. Biol. Evol.* **32**, 244–257 (2014).

19. M. Malinsky, M. Matschiner, H. Svardal, Dsuite - Fast D-statistics and related admixture evidence from VCF files. *Mol. Ecol. Resour.* **21**, 584–595 (2021).

20. M. Malinsky, R. J. Challis, A. M. Tyers, S. Schiffels, Y. Terai, B. P. Ngatunga, E. A. Miska, R. Durbin, M. J. Genner, G. F. Turner, Genomic islands of speciation separate cichlid ecomorphs in an East African crater lake. *Science* **350**, 1493–1498 (2015).

21. M. Malinsky, H. Svardal, A. M. Tyers, E. A. Miska, M. J. Genner, G. F. Turner, R. Durbin, Whole-genome sequences of Malawi cichlids reveal multiple radiations interconnected by gene flow. *Nat. Ecol. Evol.* **2**, 1940–1955 (2018).

22. F. L. Wu, A. I. Strand, L. A. Cox, C. Ober, J. D. Wall, P. Moorjani, M. Przeworski, A comparison of humans and baboons suggests germline mutation rates do not track cell divisions. *PLoS Biol.* **18**, e3000838 (2020).

23. L. Speidel, M. Forest, S. Shi, S. R. Myers, A method for genome-wide genealogy estimation for thousands of samples. *Nat. Genet.* **51**, 1321–1329 (2019).

24. L. Excofffier, N. Marchi, D. A. Marques, R. Matthey-Doret, A. Gouy, V. C. Sousa, fastsimcoal2: Demographic inference under complex evolutionary scenarios. *Bioinformatics* **37**, 4882–4885 (2021).

25. L. Bunnefeld, L. A. F. Frantz, K. Lohse, Inferring bottlenecks from genome-wide samples of short sequence blocks. *Genetics* **201**, 1157–1169 (2015).

26. F. Schlamp, J. van der Made, R. Stambler, L. Chesebrough, A. R. Boyko, P. W. Messer, Evaluating the performance of selection scans to detect selective sweeps in domestic dogs. *Mol. Ecol.* **25**, 342–356 (2016).

27. A. M. Harris, N. R. Garud, M. DeGiorgio, Detection and classification of hard and soft sweeps from unphased genotypes by multilocus genotype identity. *Genetics* **210**, 1429–1452 (2018).

28. F. Tajima, Statistical method for testing the neutral mutation hypothesis by DNA polymorphism. *Genetics* **123**, 585–595 (1989).

29. Y. Tatsumi, M. Takeda, M. Matsuda, T. Suzuki, H. Yokoi, TALEN-mediated mutagenesis in zebrafish reveals a role for r-spondin 2 in fin ray and vertebral development. *FEBS Lett.* **588**, 4543–4550 (2014).

30. U. F. Mustapha, D.-N. Jiang, Z.-H. Liang, H.-T. Gu, W. Yang, H.-P. Chen, S.-P. Deng, T.-L. Wu, C.-X. Tian, C.-H. Zhu, G.-L. Li, Male-specific Dmrt1 is a candidate sex determination gene in spotted scat (*Scatophagus argus*). *Aquaculture* **495**, 351–358 (2018).

31. I. Nanda, M. Kondo, U. Hornung, S. Asakawa, C. Winkler, A. Shimizu, Z. Shan, T. Haaf, N. Shimizu, A. Shima, M. Schmid, M. Schartl, A duplicated copy of *DMRT1* in the sex-determining

region of the Y chromosome of the medaka, *Oryzias latipes*. *Proc. Natl. Acad. Sci. U.S.A.* **99**, 11778–11783 (2002).

32. Z. Cui, Y. Liu, W. Wang, Q. Wang, N. Zhang, F. Lin, N. Wang, C. Shao, Z. Dong, Y. Li, Y. Yang, M. Hu, H. Li, F. Gao, Z. Wei, L. Meng, Y. Liu, M. Wei, Y. Zhu, H. Guo, C. H. K. Cheng, M. Schartl, S. Chen, Genome editing reveals *dmrt1* as an essential male sex-determining gene in Chinese tongue sole (*Cynoglossus semilaevis*). *Sci. Rep.* **7**, 42213 (2017).

33. S. Yoshimoto, N. Ikeda, Y. Izutsu, T. Shiba, N. Takamatsu, M. Ito, Opposite roles of DMRT1 and its W-linked paralogue, DM-W, in sexual dimorphism of *Xenopus laevis*: Implications of a ZZ/ZW-type sex-determining system. *Development* **137**, 2519–2526 (2010).

34. C. A. Smith, K. N. Roeszler, T. Ohnesorg, D. M. Cummins, P. G. Farlie, T. J. Doran, A. H. Sinclair, The avian Z-linked gene DMRT1 is required for male sex determination in the chicken. *Nature* **461**, 267–271 (2009).

35. J. Liu, C. Lin, A. Gleiberman, K. A. Ohgi, T. Herman, H. P. Huang, M. J. Tsai, M. G. Rosenfeld, Tbx19, a tissue-selective regulator of POMC gene expression. *Proc. Natl. Acad. Sci. U.S.A.* **98**, 8674–8679 (2001).

36. I. Ziegler, The pteridine pathway in zebrafish: Regulation and specification during the determination of neural crest cell-fate. *Pigment Cell Res.* **16**, 172–182 (2003).

37. E. S. Mo, Q. Cheng, A. V. Reshetnyak, J. Schlessinger, S. Nicoli, Alk and Ltk ligands are essential for iridophore development in zebrafish mediated by the receptor tyrosine kinase Ltk. *Proc. Natl. Acad. Sci. U.S.A.* **114**, 12027–12032 (2017).

38. A. Fadeev, P. Mendoza-Garcia, U. Irion, J. Guan, K. Pfeifer, S. Wiessner, F. Serluca, A. P. Singh, C. Nüsslein-Volhard, R. H. Palmer, ALKALs are in vivo ligands for ALK family receptor tyrosine kinases in the neural crest and derived cells. *Proc. Natl. Acad. Sci. U.S.A.* **115**, E630–E638 (2018).

39. A. Daruwalla, P. D. Kiser, Structural and mechanistic aspects of carotenoid cleavage dioxygenases (CCDs). *Biochim. Biophys. Acta Mol. Cell Biol. Lipids* **1865**, 158590 (2020).

40. B. Charlesworth, D. Charlesworth, The degeneration of Y chromosomes. *Philos. Trans. R. Soc. Lond. B Biol. Sci.* **355**, 1563–1572 (2000).

41. M. A. Wilson Sayres, Genetic diversity on the sex chromosomes. *Genome Biol. Evol.* **10**, 1064–1078 (2018).

42. W. J. Gammerdinger, T. D. Kocher, Unusual diversity of sex chromosomes in African cichlid fishes. *Genes* **9**, 480 (2018).

43. I. Nanda, U. Hornung, M. Kondo, M. Schmid, M. Schartl, Common spontaneous sex-reversed XX males of the medaka *Oryzias latipes*. *Genetics* **163**, 245–251 (2003).

44. T. Myosho, H. Otake, H. Masuyama, M. Matsuda, Y. Kuroki, A. Fujiyama, K. Naruse, S. Hamaguchi, M. Sakaizumi, Tracing the emergence of a novel sex-determining gene in medaka, *Oryzias luzonensis*. *Genetics* **191**, 163–170 (2012).

45. R. S. Hattori, Y. Murai, M. Oura, S. Masuda, S. K. Majhi, T. Sakamoto, J. I. Fernandino, G. M. Somoza, M. Yokota, C. A. Strüssmann, A Y-linked anti-Müllerian hormone duplication takes over a critical role in sex determination. *Proc. Natl. Acad. Sci. U.S.A.* **109**, 2955–2959 (2012).

46. G. A. Lucas, thesis, Iowa State University, Ames, Iowa (1968).

47. H. Helgeland, M. Sodeland, N. Zoric, J. S. Torgersen, F. Grammes, J. von Lintig, T. Moen, S. Kjøglum, S. Lien, D. I. Våge, Genomic and functional gene studies suggest a key role of beta-carotene oxygenase 1 like (*bco1l*) gene in salmon flesh color. *Sci. Rep.* **9**, 20061 (2019).

48. M. A. Gazda, P. M. Araújo, R. J. Lopes, M. B. Toomey, P. Andrade, S. Afonso, C. Marques, L. Nunes, P. Pereira, S. Trigo, G. E. Hill, J. C. Corbo, M. Carneiro, A genetic mechanism for sexual dichromatism in birds. *Science* **368**, 1270–1274 (2020).

49. G. Khoo, T. M. Lim, V. P. E. Phang, Cellular basis of metallic iridescence in the Siamese fighting fish, *Betta splendens*, in *The Israeli Journal of Aquaculture-Bamidgeh* (2014), **65**; https://evols.library.manoa.hawaii.edu/handle/10524/49087.

50. A. Ng, R. A. Uribe, L. Yieh, R. Nuckels, J. M. Gross, Zebrafish mutations in *gart* and *paics* identify crucial roles for de novo purine synthesis in vertebrate pigmentation and ocular development. *Development* **136**, 2601–2611 (2009).

51. T. Kimura, Y. Nagao, H. Hashimoto, Y.-I. Yamamoto-Shiraishi, S. Yamamoto, T. Yabe, S. Takada, M. Kinoshita, A. Kuroiwa, K. Naruse, Leucophores are similar to xanthophores in their specification and differentiation processes in medaka. *Proc. Natl. Acad. Sci. U.S.A.* **111**, 7343–7348 (2014).

52. K. A. Hultman, E. H. Budi, D. C. Teasley, A. Y. Gottlieb, D. M. Parichy, S. L. Johnson, Defects in ErbB-dependent establishment of adult melanocyte stem cells reveal independent origins for embryonic and regeneration melanocytes. *PLOS Genet.* **5**, e1000544 (2009).

53. H. B. Goodrich, R. N. Mercer, Genetics and colors of the Siamese fighting fish, *Betta splendens*. *Science* **79**, 318–319 (1934).

54. H. M. Wallbrunn, Genetics of the Siamese fighting fish, *Betta splendens*. *Genetics* **43**, 289–298 (1958).

55. M. Sudol, K. F. Harvey, Modularity in the Hippo signaling pathway. *Trends Biochem. Sci.* **35**, 627–633 (2010).

56. T. L. Hoffman, A. L. Javier, S. A. Campeau, R. D. Knight, T. F. Schilling, Tfap2 transcription factors in zebrafish neural crest development and ectodermal evolution. *J. Exp. Zool. B Mol. Dev. Evol.* **308**, 679–691 (2007).

57. T. P. Lowe, J. R. Larkin, Sex reversal in *Betta splendens* Regan with emphasis on the problem of sex determination. *J. Exp. Zool.* **191**, 25–31 (1975).

58. C. A. Wilson, S. K. High, B. M. McCluskey, A. Amores, Y.-L. Yan, T. A. Titus, J. L. Anderson, P. Batzel, M. J. Carvan 3rd, M. Schartl, J. H. Postlethwait, Wild sex in zebrafish: Loss of the natural sex determinant in domesticated strains. *Genetics* **198**, 1291–1308 (2014).

59. A. Lindholm, F. Breden, Sex chromosomes and sexual selection in poeciliid fishes. *Am. Nat.* **160** Suppl 6, S214–S224 (2002).

60. C.-S. Chin, P. Peluso, F. J. Sedlazeck, M. Nattestad, G. T. Concepcion, A. Clum, C. Dunn, R. O'Malley, R. Figueroa-Balderas, A. Morales-Cruz, G. R. Cramer, M. Delledonne, C. Luo, J. R. Ecker, D. Cantu, D. R. Rank, M. C. Schatz, Phased diploid genome assembly with single-molecule real-time sequencing. *Nat. Methods* **13**, 1050–1054 (2016).

61. A. C. English, S. Richards, Y. Han, M. Wang, V. Vee, J. Qu, X. Qin, D. M. Muzny, J. G. Reid, K. C. Worley, R. A. Gibbs, Mind the gap: Upgrading genomes with Pacific Biosciences RS long-read sequencing technology. *PLOS ONE* **7**, e47768 (2012).

62. E. Garrison, G. Marth, Haplotype-based variant detection from short-read sequencing. arXiv [q-bio.GN] (2012); http://arxiv.org/abs/1207.3907.

63. M. Vasimuddin, S. Misra, H. Li, S. Aluru, in *2019 IEEE International Parallel and Distributed Processing Symposium (IPDPS)* (2019), pp. 314–324.

64. H. Li, A statistical framework for SNP calling, mutation discovery, association mapping and population genetical parameter estimation from sequencing data. *Bioinformatics* **27**, 2987–2993 (2011).

65. A. McKenna, M. Hanna, E. Banks, A. Sivachenko, K. Cibulskis, A. Kernytsky, K. Garimella, D. Altshuler, S. Gabriel, M. Daly, M. A. DePristo, The Genome Analysis Toolkit: A MapReduce framework for analyzing next-generation DNA sequencing data. *Genome Res.* **20**, 1297–1303 (2010).

66. J. T. Robinson, H. Thorvaldsdóttir, W. Winckler, M. Guttman, E. S. Lander, G. Getz, J. P. Mesirov, Integrative genomics viewer. *Nat. Biotechnol.* **29**, 24–26 (2011).

67. M. Martin, M. Patterson, S. Garg, S. O. Fischer, N. Pisanti, G. W. Klau, A. Schöenhuth, T. Marschall, WhatsHap: Fast and accurate read-based phasing. *bioRxiv* 085050 (2016). https://doi.org/10.1101/085050.

68. O. Delaneau, J.-F. Zagury, M. R. Robinson, J. L. Marchini, E. T. Dermitzakis, Accurate, scalable and integrative haplotype estimation. *Nat. Commun.* **10**, 5436 (2019).

69. S. Purcell, B. Neale, K. Todd-Brown, L. Thomas, M. A. R. Ferreira, D. Bender, J. Maller, P. Sklar, P. I. W. de Bakker, M. J. Daly, P. C. Sham, PLINK: A tool set for whole-genome association and population-based linkage analyses. *Am. J. Hum. Genet.* **81**, 559–575 (2007).

70. B. Q. Minh, H. A. Schmidt, O. Chernomor, D. Schrempf, M. D. Woodhams, A. von Haeseler, R. Lanfear, IQ-TREE 2: New models and efficient methods for phylogenetic inference in the genomic era. *Mol. Biol. Evol.* **37**, 1530–1534 (2020).

71. G. Nilsen, K. Liestøl, P. Van Loo, H. K. Moen Vollan, M. B. Eide, O. M. Rueda, S.-F. Chin, R. Russell, L. O. Baumbusch, C. Caldas, A.-L. Børresen-Dale, O. C. Lingjaerde, Copynumber: Efficient algorithms for single- and multi-track copy number segmentation. *BMC Genomics* **13**, 591 (2012).

72. J. Kelleher, A. M. Etheridge, G. McVean, Efficient coalescent simulation and genealogical analysis for large sample sizes. *PLoS Comput. Biol.* **12**, e1004842 (2016).

73. N. R. Garud, P. W. Messer, E. O. Buzbas, D. A. Petrov, Recent selective sweeps in North American *Drosophila melanogaster* show signatures of soft sweeps. *PLOS Genet.* **11**, e1005004 (2015).

74. A. Harpak, N. Garud, N. A. Rosenberg, D. A. Petrov, M. Combs, P. S. Pennings, J. Munshi-South, Genetic adaptation in New York city rats. *Genome Biol. Evol.* **13**, evaa247 (2021).

75. X. Zhou, M. Stephens, Genome-wide efficient mixed-model analysis for association studies. *Nat. Genet.* **44**, 821–824 (2012).

76. A. Rahman, I. Hallgrímsdóttir, M. Eisen, L. Pachter, Association mapping from sequencing reads using *k*-mers. *eLife* **7**, e32920 (2018).

77. S. D. Jackman, B. P. Vandervalk, H. Mohamadi, J. Chu, S. Yeo, S. A. Hammond, G. Jahesh, H. Khan, L. Coombe, R. L. Warren, I. Birol, ABySS 2.0: Resource-efficient assembly of large genomes using a Bloom filter. *Genome Res.* **27**, 768–777 (2017).

78. S. Picelli, A. K. Björklund, B. Reinius, S. Sagasser, G. Winberg, R. Sandberg, Tn5 transposase and tagmentation procedures for massively scaled sequencing projects. *Genome Res.* **24**, 2033–2040 (2014).

79. R. Corbett-Detig, R. Nielsen, A hidden markov model approach for simultaneously estimating local ancestry and admixture time using next generation sequence data in samples of arbitrary ploidy. *PLOS Genet.* **13**, e1006529 (2017).

80. K. W. Broman, H. Wu, S. Sen, G. A. Churchill, R/qtl: QTL mapping in experimental crosses. *Bioinformatics* **19**, 889–890 (2003).

81. K. Ichikawa, S. Tomioka, Y. Suzuki, R. Nakamura, K. Doi, J. Yoshimura, M. Kumagai, Y. Inoue, Y. Uchida, N. Irie, H. Takeda, S. Morishita, Centromere evolution and CpG methylation during vertebrate speciation. *Nat. Commun.* **8**, 1833 (2017).

82. C. Kowasupat, B. Panijpan, P. Ruenwongsa, T. Jeenthong, *Betta siamorientalis,* a new species of bubble-nest building fighting fish (Teleostei: Osphronemidae) from eastern Thailand. *Vertebr. Zool.* **62**, 387–397 (2012).

83. A. Monvises, B. Nuangsaeng, N. Sriwattanarothai, B. Panijpan, The Siamese fighting fish: Well-known generally but little-known scientifically. *Sci. Asia* **35**, 8–16 (2009).

84. J. M. Flynn, R. Hubley, C. Goubert, J. Rosen, A. G. Clark, C. Feschotte, A. F. Smit, RepeatModeler2 for automated genomic discovery of transposable element families. *Proc. Natl. Acad. Sci. U.S.A.* **117**, 9451–9457 (2020).

85. G. Marçais, A. L. Delcher, A. M. Phillippy, R. Coston, S. L. Salzberg, A. Zimin, MUMmer4: A fast and versatile genome alignment system. *PLoS Comput. Biol.* **14**, e1005944 (2018).

86. M. Chakraborty, N. W. VanKuren, R. Zhao, X. Zhang, S. Kalsow, J. J. Emerson, Hidden genetic variation shapes the structure of functional elements in *Drosophila*. *Nat. Genet.* **50**, 20–25 (2018).

87. Y. M. Kwon, K. Gori, N. Park, N. Potts, K. Swift, J. Wang, M. R. Stammnitz, N. Cannell, A. Baez-Ortega, S. Comte, S. Fox, C. Harmsen, S. Huxtable, M. Jones, A. Kreiss, C. Lawrence, B. Lazenby, S. Peck, R. Pye, G. Woods, M. Zimmermann, D. C. Wedge, D. Pemberton, M. R. Stratton, R. Hamede, E. P. Murchison, Evolution and lineage dynamics of a transmissible cancer in Tasmanian devils. *PLoS Biol.* **18**, e3000926 (2020).

88. D. H. Alexander, J. Novembre, K. Lange, Fast model-based estimation of ancestry in unrelated individuals. *Genome Res.* **19**, 1655–1664 (2009).

89. A. Bergström, S. A. McCarthy, R. Hui, M. A. Almarri, Q. Ayub, P. Danecek, Y. Chen, S. Felkel, P. Hallast, J. Kamm, H. Blanché, J.-F. Deleuze, H. Cann, S. Mallick, D. Reich, M. S. Sandhu, P. Skoglund, A. Scally, Y. Xue, R. Durbin, C. Tyler-Smith, Insights into human genetic variation and population history from 929 diverse genomes. *Science* **367**, eaay5012 (2020).

90. A. J. Dobson, K. Kuulasmaa, E. Eberle, J. Scherer, Confidence intervals for weighted sums of Poisson parameters. *Stat. Med.* **10**, 457–462 (1991).

91. A. Dobin, C. A. Davis, F. Schlesinger, J. Drenkow, C. Zaleski, S. Jha, P. Batut, M. Chaisson, T. R. Gingeras, STAR: Ultrafast universal RNA-seq aligner. *Bioinformatics* **29**, 15–21 (2013).

92. B. Li, V. Ruotti, R. M. Stewart, J. A. Thomson, C. N. Dewey, RNA-Seq gene expression estimation with read mapping uncertainty. *Bioinformatics* **26**, 493–500 (2010).

93. M. I. Love, W. Huber, S. Anders, Moderated estimation of fold change and dispersion for RNA-seq data with DESeq2. *Genome Biol.* **15**, 550 (2014).

94. L. D. Thomas, S. Bandara, V. M. Parmar, R. Srinivasagan, N. Khadka, M. Golczak, P. D. Kiser, J. von Lintig, The human mitochondrial enzyme BCO2 exhibits catalytic activity toward carotenoids and apocarotenoids. *J. Biol. Chem.* **295**, 15553–15565 (2020).

95. J. von Lintig, K. Vogt, Filling the gap in vitamin A research. Molecular identification of an enzyme cleaving beta-carotene to retinal. *J. Biol. Chem.* **275**, 11915–11920 (2000).

96. A. Daruwalla, J. Zhang, H. J. Lee, N. Khadka, E. R. Farquhar, W. Shi, J. von Lintig, P. D. Kiser, Structural basis for carotenoid cleavage by an archaeal carotenoid dioxygenase. *Proc. Natl. Acad. Sci. U.S.A.* **117**, 19914–19925 (2020).
